# Midbrain degeneration triggers astrocyte reactivity and tau pathology in experimental Alzheimer’s Disease

**DOI:** 10.1186/s13024-025-00893-2

**Published:** 2025-10-13

**Authors:** Livia La Barbera, Paraskevi Krashia, Gilda Loffredo, Emma Cauzzi, Maria Luisa De Paolis, Martina Montanari, Luana Saba, Elena Spoleti, Serena Ficchì, Claudio Zaccone, Marco De Bardi, Claudia Palazzo, Ramona Marino, Emanuele Claudio Latagliata, Stefano Puglisi-Allegra, Giovanna Borsellino, Flavio Keller, Luisa Lo Iacono, Maria Teresa Viscomi, Annalisa Nobili, Marcello D’Amelio

**Affiliations:** 1 Department of Medicine and Surgery, Università Campus Bio-Medico Di Roma, Via Alvaro del Portillo, 21–00128, Rome, Italy; 2 Department of Experimental Neurosciences, IRCCS Santa Lucia Foundation, Via del Fosso Di Fiorano, 64–00143, Rome, Italy; 3 Department of Sciences and Technologies for Sustainable Development and One Health, Università Campus Bio-Medico Di Roma, Via Alvaro del Portillo, 21–00128, Rome, Italy; 4 Wyss Center for Bio and Neuro Engineering, Geneva, 1202 Switzerland; 5 Department of Life Science and Public Health, Section of Histology and Embryology, Università Cattolica del Sacro Cuore, Largo Francesco Vito, 1–00168, Rome, Italy; 6 Department of Psychology, Università Telematica Internazionale Uninettuno, Corso Vittorio Emanuele II, Rome, Italy; 7 Istituto Di Ricovero E Cura a Carattere Scientifico (IRCCS) Neuromed, Via Atinense, 18–86077, Pozzilli, Italy; 8 Department of Translational Research and of New Surgical and Medical Technologies, Università Di Pisa Unipi, Via Risorgimento, 36–56126, Pisa, Italy; 9 Fondazione Policlinico Universitario “A. Gemelli”, IRCCS, Largo Francesco Vito, 1–00168, Rome, Italy

**Keywords:** Neurodegenerative disease, Cognitive decline, Mesolimbic system, Monoamines, Gene expression profiling, Transcriptome, Synaptic plasticity, SSRI

## Abstract

**Background:**

Smaller midbrain volumes predict Alzheimer’s Disease (AD) progression and faster conversion from Mild Cognitive Impairment (MCI) to dementia. Along with this, various midbrain-target areas are characterized by neuroinflammation since the MCI stage. The concomitance of neuroinflammation, Αβ and tau appears to be a strong predictor for conversion from MCI to dementia.

Yet, how midbrain degeneration could cause disease progression, and what mechanisms are involved in triggering neuroinflammation in midbrain-target areas such as the hippocampus remain unexplored.

**Methods:**

Using adult C57BL/6N mice we generated a new mouse model carrying lesions in three midbrain nuclei, the dopaminergic Ventral Tegmental Area (VTA) and Substantia Nigra *pars compacta* (SNpc) and the serotonergic Interpeduncular Nucleus (IPN), to evaluate the consequences of dopamine and serotonin deprivation in midbrain-target areas. We characterized this model by performing stereological cell counts, analysis of monoaminergic fibers, monoamine levels, electrophysiology and behavioral tests. We then assessed hippocampal neuroinflammation by analyzing glia cell count, changes in morphology, NLRP3 inflammasome activation and cytokine levels, and microglia transcriptional profiling. In a separate set of experiments, we induced experimental midbrain lesion in Tg2576 transgenic mice overexpressing the Swedish mutant amyloid precursor protein, to evaluate the effect of monoamine deprivation on the hippocampus in concomitance with amyloid-β (Aβ) accumulation. The lesion performed in Tg2576 mice, as opposed to that in C57BL/6N mice, provides valuable insights into how neuroinflammation is influenced by Aβ accumulation versus the exclusive impact of impaired monoaminergic signaling.

**Results:**

The concomitant depletion of dopaminergic and serotonergic inputs within the hippocampus of C57BL/6N mice provokes a pronounced activation of microglia *via* the NLRP3-inflammasome pathway, accompanied by increased IL-1β expression. Pharmacological intervention with either dopaminergic (L-DOPA or A68930) or serotonergic (fluoxetine) agents abrogates this neuroinflammatory response. In the Tg2576 transgenic mouse model of amyloid pathology, which exhibits progressive Aβ deposition, superimposed midbrain degeneration markedly amplifies AD-like neuropathology. This includes exacerbation of microglial reactivity, robust astrocyte response, precocious Aβ plaque burden, and induction of pathological tau hyperphosphorylation. Notably, administration of L-DOPA or fluoxetine significantly attenuates both the astrocyte reactivity and tau hyperphosphorylation in the lesioned Tg2576 cohort.

**Conclusions:**

These results highlight the pivotal role of midbrain damage for the amplification of neuroinflammatory cascades and AD pathology. Moreover, they offer mechanistic insight into the faster progression to dementia in patients with midbrain deficits. By translating these findings into clinical practice, we can advance towards a precision medicine approach in disease management.

**Supplementary Information:**

The online version contains supplementary material available at 10.1186/s13024-025-00893-2.

## Background

Alzheimer’s Disease (AD) is increasingly recognized as a pathophysiological process that begins up to 20 years before the onset of cognitive decline [1]. During this preclinical phase of the *AD continuum* there is a progressive accumulation of Amyloid-β (Αβ) in the brain, that precedes tau pathology [2–4]. In fact, individuals with Aβ pathology alone, in the absence of tau pathology, may not clinically convert to AD, thus remaining cognitively unimpaired. Instead, future cognitive decline is highly related to the appearance of secondary tauopathy.

Recent studies hint that neuroinflammation, mediated by microglia and/or astrocyte reactivity, is the driver for tau hyperphosphorylation [4–10]. Indeed, the co-occurrence of neuroinflammation with Αβ and tau appears to be the strongest predictor of cognitive impairment leading to dementia [7, 11, 12]. This intricate relationship suggests a hypothetical model of disease progression in which conversion to dementia occurs in Αβ-positive individuals who are positive both for neuroinflammatory markers and tauopathy. Within this scenario, identification of key biological players that induce or worsen the neuroinflammatory response would allow for the recognition of new potential targets for early intervention against cognitive decline.

Recent experimental and clinical studies point to early vulnerability of brainstem monoaminergic systems as a potential contributor to neuroinflammatory cascades in AD [13–19]. Neuromodulatory monoaminergic nuclei, such as the noradrenergic Locus Coeruleus (LC) and the serotonergic Raphe, have been extensively implicated in the pathophysiology of AD. In parallel, emerging evidence underscores that midbrain monoaminergic nuclei releasing dopamine (DA) and, to a lesser extent, serotonin (5-HT), are also precociously affected in the disease course. Specifically, midbrain dopaminergic pathways exhibit pronounced vulnerability, manifesting as atrophy of the Ventral Tegmental Area (VTA) and as reduced functional connectivity with cortical regions, alongside hypometabolism and neuroinflammatory alterations in mesocorticolimbic targets [20–29]. These deficits are detectable as early as the Mild Cognitive Impairment (MCI) stage and temporally coincide with the emergence of neuropsychiatric symptoms (e.g., apathy, depression, and anxiety), reflecting mesencephalic circuit disruption. Furthermore, longitudinal studies reveal that MCI subjects presenting smaller midbrain volumes or greater baseline functional disconnection, progress more rapidly to dementia [22, 24–27, 30–35]. Of note, in cognitively unimpaired individuals, hippocampal volume and memory performance positively correlate with VTA integrity, underscoring the importance of mesocorticolimbic connectivity in sustaining cognitive function [30]. These new findings prompted us to investigate the hypothesis that damage to specific midbrain nuclei producing DA and 5-HT may trigger neuroinflammation and, thus, promote AD-related pathology.

Thus, here we focused on the hippocampus, a pivotal hub for cognition and memory, strongly modulated by DA and 5-HT [36–40], and one of the first brain regions to be affected in AD patients. Following experimental midbrain lesion, we evaluated the effects of monoamine deprivation in the hippocampus of both non-AD mice (C57BL/6N) and pre-plaque-stage Tg2576 mice, a validated AD model overexpressing the human Aβ Precursor Protein (APP) carrying the Swedish mutation (KM670/671NL; APPSwe), to disentangle the potential neuroinflammation due to monoaminergic deprivation from that due to Aβ load.

We provide evidence that the combined drop of DA and 5-HT induces significant hippocampal neuroinflammation in C57BL/6N mice, that is characterized by microglia reactivity and interleukin-1β (IL-1β) release mediated by the NOD-, LRR- and Pyrin domain-containing protein 3 (NLRP3)-inflammasome pathway. Dopaminergic or serotonergic drugs can suppress this neuroinflammatory process. Importantly, we show that when the damage of the same dopaminergic and serotonergic midbrain nuclei is induced in Tg2576 mice, the AD-like phenotype is strongly accelerated with a hyper-inflammatory phenotype associated with exacerbated microglial reactivity, induction of astrocyte reactivity, increased levels of Aβ and appearance of a secondary tau pathology. Crucially, administration of L‑DOPA or fluoxetine substantially attenuates both astrocyte reactivity and tau hyperphosphorylation, underscoring the therapeutic potential of restoring the monoaminergic tone to mitigate AD progression.

## Methods

### Animals

Experiments complied with the ARRIVE procedures and the ethical guidelines of the European Council Directive (2010/63/EU). Experimental approval was obtained from the Italian Ministry of Health. Male and female C57BL/6N (Charles River, Italy) and female B6.129P2(Cg)-Cx3cr1^tm1Litt^/J (CX_3_CR-1^GFP^ knock-in/knock-out; Jackson Laboratory Strain #:005582) mice were used at 2–3 months of age. Heterozygous male Tg2576 mice (Taconic #APPSWE—Model 1349 tg/wt; [41]) and their Wild-Type (WT) littermates (mice with the same strain and genetic background of Tg2576, negative for APPSWE overexpression—Taconic #APPSWE—Model 1349 wt/wt) were used at 6–7 months of age.

All mice were housed with *ad* *libitum* food and water, with a 12 h light/dark cycle. Mice were housed in cages of 3–4 individuals and all cages were equipped with the same environmental enrichment.

### Stereotaxic injections

Mice were anaesthetized with Rompun (20 mg/mL, 0.5 mL/kg; Bayer) and Zoletil (100 mg/mL, 0.5 mL/kg; Virbac; intraperitoneally i.p.) and positioned in a stereotaxic apparatus.

For Caspase-3 (Casp3) mice (C57BL/6N, CX_3_CR-1^GFP^ or Tg2576 mice), we infused a mix of Adeno-Associated Viruses (AAVs; 0.5 µL each, flux 80 nL/min [42]: (i) AAV1-THp-iCre (5 × 10^12^ viral particles/mL; Vector Biolabs) and (ii) AAV5-flex-taCasp3-TEVp (4.2 × 10^12^ viral particles/mL; UNC Vector core, gift from Nirao Shah) in the left VTA (AP: −3.2, ML: −0.35, DV: −4.4; 43), to lesion Tyrosine Hydroxylase-positive (TH^+^) neurons in the VTA and Substantia Nigra *pars compacta* (SNpc) and 5-HT^+^ neurons in the interpeduncular nucleus (IPN). Sham mice were injected only with the AAV1-THp-iCre virus.

In a separate cohort of C57BL/6N mice, the AAV1-THp-iCre + AAV5-flex-taCasp3-TEVp mix (0.125 µL each, flux 25 nL/min) were injected in the IPN (thereafter: Casp3^IPN^; AP: −3.5, ML: 0, DV: −4.7; [43]) to lesion the 5-HT^+^ neurons of the IPN while leaving intact the TH^+^ neurons of the VTA/SNpc. Sham mice were injected only with the AAV1-THp-iCre virus (Sham^IPN^).

To confirm the ectopic expression of TH promoter-driven AAVs, a set of C57BL/6N mice were infused in the VTA with a mix (0.5 µL each, flux 80 nL/min) of (i) AAV1-THp-iCre and (ii) AAV5-EF1α-DIO-eYFP (4.0 × 10^12^ viral particles/mL; UNC Vector core, gift from Karl Deisseroth). eYFP expression was then analyzed with confocal microscopy (see below).

For 6-hydroxy-dopamine (6OHDA) lesion (C57BL/6N mice), 6OHDA (Sigma-Aldrich; 7.6 mg/mL, calculated as free-base) was dissolved in 0.2 mg/mL ascorbic-acid (Tocris) prepared in 0.9% saline, and continuously kept on ice. Each mouse was injected with 2.5 μg in 0.4 µL (flux 40 nL/min) unilaterally in the left VTA. Thirty minutes before surgery, mice received 10 mL/kg (2.85 mg/mL as free base, i.p.) of the norepinephrine (NE) reuptake-inhibitor desipramine-hydrochloride (Sigma-Aldrich), to prevent NE fiber degeneration. Control mice were injected with Saline.

C57BL/6N and CX_3_CR-1^GFP^ mice received the intracerebral injection at 2 months of age. Tg2576 mice were injected at 6 months of age. All mice were used 30 days following injection to ensure steady-state lesion. A separate set of Casp3 and Sham were used at 72 h following lesion; in these mice we examined the levels of Casp3 protein in the midbrain (see below).

For retrograde labelling, red retrobeads (120 nL; 12 nL/min; Lumafluor) were injected bilaterally in the *Nucleus Accumbens* (NAc) medial shell (AP: 1.8, ML: ± 0.4, DV: 4.1), NAc core (AP: 1.5, ML: ± 0.9, DV: 4.1) and dorsal striatum (AP: 1.0, ML: ± 1.6, DV: 2.3). Mice were examined 1 month after surgery.

For all infusions we used 1 μL Hamilton syringes (Model Neuros7001) mounted on a Pump-11 Elite Nanomite (Harvard Apparatus). When possible, the accuracy of the injection site was controlled by immunofluorescence analysis; mice with misplaced injection were excluded.

### Drug treatments

Animals were injected with L-DOPA (i.p., 10 mg/kg; Sigma-Aldrich) plus benserazide (12 mg/kg; Sigma-Aldrich) [44], or with A68930-hydrochloride (5 mg/kg; Santa Cruz), once a day for 4 or 7 days, respectively; 0.9% saline was used as control (vehicle); experiments were performed 1 h after the last injection.

Fluoxetine-hydrochloride (30 mg/kg; Tocris) was dissolved in water and delivered *ad* *libitum* in drinking bottles wrapped in tin-foil for 30 days, starting immediately after surgery. Normal drinking water was used as control.

### Immunofluorescence

Anaesthetized mice (Rompun/Zoletil) were transcardially-perfused with Phosphate Buffer (PB; 0.1 M, pH 7.4) followed by 4% paraformaldehyde in PB. Brains were postfixed in 4% paraformaldehyde for at least 4 h, dehydrated and cryoprotected in 30% sucrose in PB at 4 °C until sinking. Thirty μm-thick coronal sections were cut with a cryostat, and slices were collected in PB-sodium azide 0.02%. All analyses were performed in the left hemisphere, ipsilateral to the lesion.

Slices were incubated with primary antibodies in PB containing 0.3% Triton X-100 overnight at 4 °C for TH, Iba1, GFAP, hippocampal 5-HT transporter (SERT) and striatal TH/DA transporter (DAT)/SERT staining, or 3 nights for IL-1β/Iba1, IL-18/Iba1, IL-18 Receptor (IL-18R)/GFAP, p-Nuclear Factor kappa B (p-NFκB)/S100β and Microtubule-Associated Protein 2 (MAP2).

For 5-HT, slices were incubated with primary antibodies in PB containing 0.5% Triton X-100 and 10% donkey-serum for 2 nights at 4 °C.

For hippocampal TH^+^/NE transporter-positive (NET^+^) fibers, sections were incubated in citrate buffer (10 mM sodium-citrate, pH 6.0 containing 0.05% Tween-20; 20 min, 75 °C), rinsed in PB, immersed in blocking solution (5% donkey serum, 0.2% Triton X-100 in PB; 1 h, RT), and incubated with primary antibody in the same solution (overnight at 4 °C; [45]).

For NLRP3/Iba1/GFAP and NLRP3/Iba1 staining, sections were pretreated with 50% methanol (15 min, RT) immersed in blocking solution (3% bovine serum albumin, 0.1% Triton X-100 in PB, 30 min, RT) and incubated with primary antibodies in PB with 0.1% Triton X-100 (2 nights, 4 °C).

For Iba1/CD68, slices were permeabilized using 0.5% Triton X-100 in PB (45 min, RT), incubated in blocking solution (2% bovine-serum albumin, 0.5% Triton X-100 in PB; 1 h, RT) and exposed to primary antibodies in blocking solution (2 nights, 4 °C).

For C3/GFAP, slices were immersed in blocking solution (5% donkey serum, 0.1% Triton X-100 in PB; 1 h, RT) and then incubated with primary antibodies in 1% donkey serum, 0.1% Triton X-100 in PB (overnight, 4 °C).

For C3aR/Iba1/NeuroTrace analysis, slices were incubated in blocking solution (3% bovine-serum albumin, 5% goat serum, 0.5% Triton X-100 in PB; 1 h, RT) and then with primary antibodies in blocking solution (overnight, 4 °C).

For tau staining (AT8), slices were pretreated with 50% methanol (15 min, RT), immersed in blocking solution (3% bovine serum albumin, 0.1% Triton X-100 in PB) with M.O.M.® (Mouse on Mouse) Blocking Reagent (1:1000; Vector laboratories, #MKB-2213–1; 1 h, RT) and then incubated with primary antibodies in 0.1% Triton X-100 in PB (2 nights, 4 °C).

For Aβ staining (6E10), sections were pretreated with M.O.M.® (1:1000; 2 h, RT) diluted in permeabilization solution (PB with 0.3% Triton X-100) and incubated with primary antibodies overnight at 4 °C in permeabilization solution.

For eYFP expression, coronal sections containing the midbrain, Raphe and LC were stained with TH/5-HT, 5-HT and TH, respectively as described above.

For every immunofluorescence protocol, after primary antibody, slices were incubated with secondary antibodies in the same solution of primary antibody (2 h, RT) and counterstained with Blue-Fluorescent Nissl-Stain (NeuroTrace 1:200; Invitrogen) or DAPI (1:1000, Serva).

After mounted, slices were examined using a Nikon Eclipse Ti2 confocal microscope. The labeling specificity was confirmed by omission of primary antibodies and use of normal serum instead (negative controls). For quantitative analysis, images were processed simultaneously and analyzed with Fiji-ImageJ (http://imagej.nih.gov/ij/): after 8-bit conversion and background subtraction, the signal was quantified by measuring the relative fluorescence intensity. The F/A ratio defines mean fluorescence intensity (F) over surface area (A).

For analysis of fiber density and intensity, and protein levels, images were acquired with a 20x-objective by Z-stacks, then processed by maximum-intensity projection. All samples were captured with identical Z-stack thickness and laser settings within each analysis.

TH^+^/DAT^+^/SERT^+^ fiber intensity was quantified by setting 16 frames (100 × 100 pixel). The total fiber number per 250 µm was counted manually [45, 46].

CD68, NLRP3, IL-1β and IL-18 protein levels were measured within Iba1^+^ cells.

Automated total cell counts and analysis of overlapping regions between markers to assess colocalization were performed in Imaris XT software (Bitplane AG, Oxford Instruments, Abingdon-on-Thames, UK). For all double-positive cell counting analyses, the Surfaces function was used. Briefly, after applying background subtraction and automatic thresholding, to retain only objects within lower and upper threshold Limits, and splitting of touching objects to separate individual cells, colocalization between markers was quantified using the Overlapped Volume Surfaces option. Complement component 3 (C3) levels were quantified within GFAP^+^ cells, while MAP2 intensity was quantified by setting 12 frames (90 × 90 pixel) over the *stratum radiatum*. Intracellular Aβ levels were quantified by setting 10 randomly-distributed frames on the hippocampal CA1 pyramidal layer (70 × 70 pixel).

To analyze the number of Aβ plaques and the AT8^+^ area in the hippocampus, we acquired Z-stack large images with a 20x-objective. The mean number of 6E10^+^ plaques in the hippocampus from at least 3 slices/animal was quantified. To calculate AT8^+^ area, the hippocampus was manually defined, and automatic brightness thresholding was used to delineate the positive area. Hippocampal AT8^+^ area was expressed as ratio of total area analyzed in each slice/animal (modified from [47]). Intracellular AT8 levels were quantified by Z-stack with 60x-oil objective and by setting 1 frame (20 × 20 pixel).

For all analyses, quantification was done on 4 slices/mouse. Data were then averaged per mouse for figures and statistical analysis.

Primary antibodies: 5-HT (1:500; ImmunoStar #20080; RRID:AB_572263), AT8 Ser202/Thr205 (1:200; Invitrogen #1020; RRID:AB_223647), C3 (1:300; Novus Biologicals #NB200-540; RRID:AB_2744548), C3aR (1:200; HycultBiotech #HM3028; RRID:AB_2131309), CD68 (1:400; Biorad #MCA1957; RRID:AB_322219), DAT (1:400; Chemicon #MAB369; RRID:AB_2190413), GFAP (1:1000; Millipore #AB5804; RRID:AB_2109645), GFAP (1:1000; DAKO #Z0334; RRID:AB_2314535), hAPP695 (6E10) (1:500; BioLegend #803001; RRID: AB_2564653), Iba1 (1:600; Wako #019–19741; RRID:AB_839504), AIF-1/Iba1 (1:600; Novus Biologicals #NB100-1028; RRID:AB_3148646), IL-1β (1:200; R&D #AF-401-NA; RRID:AB_416684), IL-18 (1:300; MBL #D047-3; RRID:AB_592016), IL-18R⍺/IL-1 r5 (1:100; R&D #AF856; RRID:AB_355664), MAP2 (1:500; Invitrogen #MA5-12,826; RRID:AB_10976831), NET (1:500; Atlas Antibodies #AMAb91116; RRID:AB_2665806), NLRP3 (1:200; Adipogen #AG-20B-0014; RRID:AB_2490202), p-NFκB (Ser536) (1:200; Cell Signaling #3033; RRID:AB_331284), S100β (1:500; SYSY #287 006; RRID:AB_2713986), SERT (1:500; Millipore #PC177L; RRID:AB_2122553), TH (1:1000; Millipore #MAB318; RRID:AB_2201528), TH (1:500; Millipore #AB152; RRID: AB_390204).

Secondary Antibodies (Thermo Fisher): Alexa Fluor-488 donkey anti-rabbit (1:200; #A-21206; RRID:AB_2535792), Alexa Fluor-555 donkey anti-rabbit (1:200; #A31572; RRID:AB_162543), Alexa Fluor-647 goat anti-rabbit (1:200; #A-31573; RRID:AB_2536183), Alexa Fluor-488 donkey anti-mouse (1:200; #R37114; RRID:AB_2556542), Alexa Fluor-555 donkey anti-mouse (1:200; #A31570; RRID:AB_2536180), Alexa Fluor-488 donkey anti-rat (1:200; #A21208; RRID:AB_2535794), Alexa Fluor-647 goat anti-rat (1:200; #A-21247; RRID:AB_141778), Alexa Fluor-488 donkey anti-goat (1:200; #A-11055; RRID:AB_2534102), Alexa Fluor-647 donkey anti-goat (1:200; #A-21447; RRID:AB_141844), Alexa Fluor-555 goat anti-chicken (1:200; # A-21437; RRID:AB_2535858).

Exclusively for the representative confocal images, after the quantitative analysis, LUTs were equally increased at the same level for all groups of a given experiment. Quantitative analyses were performed on raw images.

### Stereology

Immunofluorescence sections were used to estimate: TH^+^ neurons in the left SNpc, VTA and LC, 5-HT^+^ neurons in the entire IPN; 5-HT^+^ neurons in the dorsal (dRaphe) and medial Raphe (mRaphe); Iba1^+^ cells in the hippocampus, dorsal and ventral striatum and GFAP^+^ cells in the hippocampus. The area boundaries for VTA/SNpc/LC were defined by TH, the IPN/dRaphe/mRaphe by 5-HT, the dorsal and ventral striatum and hippocampus by DAPI staining, using the 5x-objective, in accordance to Paxinos guidelines [43].

We applied an optical fractionator stereological design using the Stereo Investigator System (MBF Bioscience). A stack of MAC5000 controller modules (Ludl Electronic Products, Ltd) was interfaced with a Zeiss Microscope Axio Imager KMAT with a motorized stage and a Zeiss Axiocam 506 mono with Working High End PC. A 3D-optical fractionator counting probe (x, y, z dimension 50 × 50 × 25 μm for TH^+^ neurons, 70 × 70 × 25 μm for 5-HT^+^ neurons and 100 × 100 × 25 μm for glia cells) was applied. Cells were marked with a 100x-oil-(VTA/SNpc) or a 40x-objective (neurons in LC/IPN/dRaphe/mRaphe; glia cells in hippocampus and striatum).

The total cell number was estimated according to the formula (Eq. 1):

1 $$\text{N }=\text{ SQ }\times (1/\text{ssf}) \times (1/\text{asf}) \times (1/\text{tsf})$$

where SQ represents the cell number counted in all optically sampled fields of the ROI, ssf is the section sampling fraction, asf is the area sampling fraction and tsf is the thickness sampling fraction.

### Morphological analysis

Microglia were imaged with a Zeiss Microscope Axio Imager KMAT with motorized stage and a camera connected to Neurolucida software (7.5v; MBF Bioscience) for quantitative 3D-analysis of the entire cell [48, 49]. Only non-overlapping cells that showed clear soma and branching were analyzed. Soma area and perimeter were measured; Sholl analysis included counting the number of dendritic intersections, nodes and endings, and dendritic lengths at fixed distances from the soma in 10 μm-spaced concentric circles originating from the soma. Analysis was done with 100x-oil objective. Nine representative cells/animal were analyzed randomly and data were averaged for each mouse.

### Total protein extraction and Western-blot analysis

The ipsilateral hippocampus was dissected from the entire brain and stored at −80 °C until the day of the experiment. The ipsilateral midbrain was isolated from 500 μm-frozen slices using a biopsy punch. Tissue was homogenized in RIPA buffer containing (in mM): 50 Tris–HCl pH 7.5, 150 NaCl, 5 MgCl_2_, 1 EDTA, 1% Triton X-100, 0.25% sodium deoxycholate, 0.1% SDS, 1 sodium-orthovanadate, 5 b-glycerophosphate, 5 NaF and protease inhibitor cocktail; samples were then sonicated four times (five strokes of 0.5 pulse/s) and incubated on ice for 30 min [50]. Samples were centrifuged (13000 g, 20 min, + 4 °C) and the supernatant’s protein concentration was determined by the Bradford method. Proteins were applied to SDS-PAGE and electroblotted on a polyvinylidene-difluoride membrane. Blotting analysis was performed using a chemiluminescence detection kit. The relative levels of immunoreactivity were determined by densitometry using ImageJ.

Primary antibodies: Caspase-3 (1:500; Cell Signaling Technology; #9662s; RRID:AB_331439), Glycogen Synthase Kinase 3β (GSK3β; 1:1000; Cell Signaling Technology; #9832s; RRID:AB_10839406); p-GSK3β (Ser9) (1:1000; Cell Signaling Technology; #9336s; RRID:AB_331405); JNK (1:1000; Cell Signaling Technology; #9252s; RRID:AB_2250373); p-JNK (Thr183/Tyr185) (1:1000; Cell Signaling Technology; #9251s; RRID:AB_331659); p-p38 (Thr180/Tyr182) (1:1000; Cell Signaling Technology; #4511s; RRID:AB_2139682); p38 (1:1000; Cell Signaling Technology, #8690s; RRID:AB_10999090); TH (1:500; Abcam, #ab112; RRID:AB_297840); β-Tubulin (1:1000; Biolegend; #801201; RRID:AB_2313773). All primary antibodies were incubated overnight except for β-Tubulin (2 h).

Secondary antibodies: goat anti-mouse IgG (1:3000; Bio-Rad; #1706516; RRID:AB_11125547), goat anti-rabbit IgG (1:3000; Bio-Rad; #1706515; RRID:AB_2617112).

Membranes were stripped using Re-Blot Plus Strong Solution (Millipore; 15 min, RT). Both groups (Shan *vs* Casp3; Tg Sham *vs* Tg Casp3) were analyzed simultaneously.

### Microglia sorting, RNA isolation and RNA-sequencing

#### Microglia sorting

Hippocampus from Sham and Casp3 CX_3_CR-1^GFP^ mice (Sham: *n* = 3; Casp3: *n* = 5 mice) was dissociated into single-cell suspensions using Adult Brain Dissociation kit (Miltenyi Biotec, #130–107-677) according to the manufacturer’s instructions. Briefly, each mouse was transcardially perfused with 1 × D-PBS^+/+^. The hippocampus of each Sham or Casp3 CX_3_CR-1^GFP^ mouse was isolated and added in half brain of a C57BL/6N mouse, to increase the quantity of tissues to dissociate. Tissues were then cut into small pieces and enzymatically and mechanically dissociated on the gentle MACS Octo Dissociator with Heaters (Miltenyi Biotec, #130–096-427) using the 37C_ABDK_01 program. Following dissociation, samples were resuspended and passed through a prewet 70 μm MACS SmartStrainer (Miltenyi Biotec, #130–110-916,) and centrifuged at 300 g for 10 min at 4 °C. Following centrifugation, the supernatant was discarded, and the debris were removed using the Debris Removal solution (Miltenyi Biotec, #130–109-398) provided by the kit. Finally, cells were resuspended in 100 μL of Running Buffer and stained for 30 min at 4 °C protected from light with the following antibodies: CD11b Monoclonal Antibody (M1/70) PE-Cyanine7 (1:100; Invitrogen, #25-0112-82; RRID: AB_469588) and PerCP anti-mouse CD45 Antibody (1:100; BioLegend #103130; RRID: AB_893339). To exclude dead cells, LIVE/DEAD Fixable Aqua Dead Cell Stain Kit (1:100; Invitrogen, #L34957) was used. Cells were washed with Running Buffer and pelleted at 1000 g for 10 min at 4 °C. Then, cells were resuspended in 400 µL of Running Buffer. Afterward, the samples were taken to FACS and the gating strategy was set to CD11b^+^CD45^+^eGFP^+^ for isolation of microglia from Sham or Casp3 CX_3_CR-1^GFP^ hippocampi. The sorting purity was to be approximately 97%.

#### RNA extraction and mRNA-Seq

Total RNA from isolated microglia was purified *via* the spin-colum based “Total RNA purification Plus Micro Kit” (Norgen Biotek, #48500), following the manufacturer instructions.

Low-input mRNA-Seq was performed by Igatech Service (Udine, Italy). Briefly, RNA samples were quantified, and quality tested by TapeStation RNA assay (Agilent Technologies). Libraries were generated using the Ovation SoLo RNA-seq Library Preparation kit (Tecan Genomics) following the manufacturer’s instructions. Final Libraries were checked with Qubit 3.0 Fluorometer (Invitrogen) and Agilent Bioanalyzer DNA assay. Libraries were then barcoded for multiplexing and sequenced on paired-end 150 bp mode on NovaSeq 6000 (Illumina) at a depth of 30 million reads. Base calling and demultiplexing was performed with Illumina BCL Convert v3.9.31. Lower quality bases and adapters were removed by Cutadapt v1.11 software [51]. Reads were aligned on reference GRCm39.112 genome with STAR [52] using default parameters. Assembling and quantitation of full-length transcripts representing multiple spliced variants for each gene locus was performed by Stringtie [53] using default parameters.

Differentially expressed genes (DEGS) were identified with DESeq2 [54]. The ToppGene suite was used with default settings to identify functional enrichment in the DEG lists (http://toppgene.cchmc.org).

### High Performance Liquid Chromatography (HPLC)

Quantification of hippocampal monoamines (DA, 5-HT, NE), and relative metabolites (DOPAC and HVA for DA, 5-HIAA for 5-HT, MOPEG for NE) was performed using an HPLC system (UltiMate® 3000, Thermo Fisher) coupled with Coulochem electrochemical detection (6011RS Ultra Coulometric Analytical Cell, Thermo Fisher). After animal sacrifice, the left hippocampus was rapidly dissected on ice and immediately stored at −80 °C until analysis. On the day of analysis frozen samples were homogenized on ice with 0.05 M HClO_4_ and antioxidant solution (containing in mM: 0.27 Na_2_EDTA, 100 acetic acid, 0.0125 ascorbic acid) in a 4:1 ratio. The homogenate was mechanically lysed, sonicated on ice and centrifuged (10000 rpm, 20 min, 4 °C). The supernatant was transferred into a new tube and the pellet was weighed. 20 µL of each sample were injected onto the HPLC-ECD (run-time: 60 min; flow-rate: 0.6 mL/min). Standards of each metabolite were prepared fresh on the day with same solutions and quantities as for tissue samples. The chromatographic separation was performed on a Hypersil GOLD aQ-C18 column (150 × 3 mm, 5 µm) fitted with an aQ-C18 drop-in guard pre-column (10 × 3 mm, 5 µm) maintained at 37 °C. The mobile phase consisted of 5% methanol and buffer solution (0.1 M Na-Phosphate, 0.1 mM Titriplex® III and 0.5 μM 1-Octanesulfonic-Acid Na-salt, pH 3.6 adjusted with 85% Ortho-Phosphoric-acid), filtered through a 0.22 µm cellulose-ester membrane (Millipore). The potential applied to both dual-inline flow-through micro-porous graphitic carbon-working electrodes was set at + 450 mV, with 1 nA-gain for ECRS1 and 10 nA-gain for ECRS2. The chromatograms were integrated with Chromeleon™ Software (7.0v; Thermo Fisher). The sample concentration of metabolites was calculated from the corresponding peak height, normalized to the pellet weight.

### Brain slicing and electrophysiology

Following head dislocation, mice were decapitated and the brain was rapidly removed; 300 μm-thick parasagittal slices containing the left dorsal hippocampus were cut (Leica VT1200S vibratome) in oxygenated (95% O_2_, 5% CO_2_) ice-cold sucrose-based solution (in mM: 3 KCl, 1.25 NaH_2_PO_4_, 26 NaHCO_3_, 10 MgSO_4_, 0.5 CaCl_2_, 25 glucose, 185 sucrose; ~ 300 mOsm, pH 7.4). Brain slices were left to recover in artificial Cerebro-Spinal Fluid (aCSF; containing in mM: 124 NaCl, 1.25 NaH_2_PO_4_-H_2_O, 26 NaHCO_3_, 3 KCl, 10 Glucose, 1 MgSO_4_, 2 CaCl_2_, ~ 300 mOsm, pH 7.4) at 32–34 °C for 40 min, and moved at RT for at least 30 min before recording [55].

For recording, each slice was placed under an Olympus BX51WI microscope and perfused with oxygenated aCSF (3-4 mL/min, 30-32 °C). The hippocampus was visualized with infrared differential-interference-contrast at 4x.

Recordings were performed using a MultiClamp700B amplifier, digitized with Digidata1550B and computer-saved with pClamp11 (Molecular Devices). Patch-pipettes (3–5 MΩ), filled with aCSF, were pulled from TW150F-4 glass tubes (WPI). Field excitatory postsynaptic potentials (fEPSPs), recorded in the stratum radiatum, were induced with Schaffer collateral stimulation (100 μs), acquired at 20 kHz and filtered off-line with a 10 kHz low-pass Bessel filter. Input–Output (I/O) curves of fEPSP slopes were obtained at 10 μA-stepped increasing stimulation every 30 s. The Paired-Pulse Ratio (PPR) was evaluated with pairs of stimuli (20–1000 ms interval), at half-maximal stimulation. For Long-Term Potentiation (LTP), after 20 min of test stimulation (half-maximal intensity, every 30 s) to assess slope stability, the slice was challenged with two trains at 100 Hz (1 s duration, 20 s interval) followed by test stimulation for 1 h. The LTP magnitude was evaluated as the fEPSP mean slope at 60 min after the conditioning trains, normalized to the mean baseline slope [45, 56, 57]. To assess the effect of the IL-1β receptor antagonist (IL-1β-Ra) on LTP, slices were pre-incubated with IL-1β-Ra (100 ng/mL, from freshly-thawed stock in PBS/bovine-serum albumin 0.1%; R&D Systems) in oxygenated aCSF for 1 h and recorded as above.

Coronal slices (240 μm-thickness) were used for spine density analysis. CA1 pyramidal neurons were filled with biocytin during whole-cell patch clamp. Freshly weighted biocytin (0.2%; Tocris) was added to intracellular solution containing (in mM): Cs-methanesulfonate 120, CsCl 15, NaCl 8, HEPES 10, EGTA 0.20, TEA-Cl 10, QX314-Cl 5, Mg-ATP 2, Na-GTP 0.3 (~ 275–285 mOsm, pH 7.4). Patch-pipettes used for cell filling had a tip resistance of 4-10 MΩ and a minimum filling time of 10 min was used for each cell. Access resistance was monitored throughout this time to check for accessibility to the cytoplasm. To avoid cell damage after filling, the electrode was removed from the slice by establishment of an outside-out patch.

### Spine density analysis

For analysis of biocytin-filled neurons, slices bearing neurons loaded with biocytin were fixed by immersion in 4% paraformaldehyde in PB (overnight, 4 °C). Then, slices were washed three times in PB and incubated with Streptavidin (1:750; Alexa Fluor 555-conjugated #S32355, Thermo Fisher; RRID:AB_2571525) and Green-Fluorescent Nissl-Stain (NeuroTrace 1:600; Invitrogen #N21480) in PB containing 0.3% Triton X-100 (overnight, 4 °C).

The Z-stack confocal images were captured using Nikon Eclipse Ti2 confocal microscope with 60x-oil objective and 4.5 digital zoom. We analyzed spines in the distal (terminal) apical dendrites.

Spine density was assessed by counting the number of spines in at least 18 segments per neuron (4 cells, 5 animals per experimental group) chosen in branch orders 2–4 of apical dendrites extending for an additional 30–60 μm away from the starting point [58]. Imaris software (9.8.2) was utilized to create 3D reconstructions of dendritic segments using the Filaments tool. The settings used for all reconstructions is the same as in [59]. An observer blinded to the experimental groups manually edited the reconstructions to include or exclude misidentified spines.

### Behavioral Testing

All behavioral tests were conducted between 09:00 a.m. and 04:00 p.m.. Mice were habituated to the experimenter through daily handling sessions for one week prior to the onset of behavioral testing. In addition, animals were acclimated to the testing room for one hour immediately before the start of each experimental session.

To minimize potential olfactory interference, the chambers and objects were cleaned between animals and/or sessions using a 5% ethanol solution.

#### Open Field and Novel Object Recognition (NOR) Tests

Testing was performed in a dimly-lit (25 lx) plexiglass open field arena (60 × 60 × 30 cm), with dark-grey walls and white floor. On Day1 (D1), each mouse was placed in the arena center and allowed to freely-explore for 10 min, during which movements were recorded. We analyzed the time spent (s) in the arena center and periphery, and the total distance traveled. Thereafter, we conducted the NOR test, consisting of habituation, training and testing [60]. During habituation, mice were familiarized with the empty arena for 10 min (D2). 24 h later (D3), mice were trained for 10 min by exposure to two identical objects (yellow wooden spheres) placed in the arena center. Mice were then returned to their home cage. Following 24 h (D4), mice were returned to the arena for the test session (10 min), during which one object was replaced by a novel object (a light-grey wooden cone). In both the training and testing sessions the animals were left to freely-explore the objects, and the exploration time was recorded, calculated as the time when they touched or climbed on an object or sniffed it at a distance of at least 2 cm. The Object Discrimination Ratio (ODR) was calculated using the following formula (Eq. 2):

2 $$ODR= \frac{\text{Time exploring the novel object}}{\text{Time exploring novel object }+\text{ Time exploring familiar object}}\text{ x }100$$

Objects were randomized and counterbalanced across groups.

#### Spatial Object Recognition (SOR) Test

The SOR test was conducted in the same circular arena previously described for the open field and NOR tasks. For this test, a high-contrast black-and-yellow striped cue was affixed to the inner wall of the arena to provide a visual local cue and enhance spatial orientation. Each mouse was tested individually across five consecutive 6-min sessions, with each session separated by a 3-min inter-trial interval in the home cage. In all sessions, animals were placed into the arena from the same starting location (protocol adapted from [61, 62]).

During session 1 (S1), mice were allowed to freely explore the empty apparatus, enabling familiarization with the environmental context and distal cues in the absence of objects. During sessions 2 to 4 (S2-S4), mice were exposed to four distinct objects (A-D) placed in fixed positions to allow the animals to encode the spatial configuration (habituation phase). The objects differed in shape, size, and material to promote discrimination and reduce object bias: (A) a yellow wooden ball, (B) a gray metal column, (C) a light-grey wooden cone, and (D) a glass jar with a perforated red cap.

Habituation to the spatial configuration was assessed by averaging the duration of contact with the four objects during sessions 2, 3, and 4 for each experimental group to provide an index of general exploratory activity. Object exploration was defined as the total time (s) the animal spent with its snout either in direct contact with the object or within approximately 2 cm of it.

Session 5 (S5) involved a spatial rearrangement: object A was moved to the previous location of object B, object B was placed in a novel, unoccupied position, while objects C and D remained in their original locations. This configuration was used to assess spatial novelty detection based on differential exploration of Displaced Objects (DO) *versus* Non-Displaced Objects (NDO), using the following formula (Eqs. 3 and 4):

3 $$\text{DO }\left[\text{S}5\right]-\text{DO }\left[\text{S}4\right]=\text{DO}$$

4 $$\text{NDO }\left[\text{S}5\right]-\text{NDO }\left[\text{S}4\right]=\text{NDO}$$

These values were used to quantify the change in exploratory behavior elicited by the spatial rearrangement.

All sessions were video-recorded using a ceiling-mounted camera, and behavioral tracking was performed offline with EthoVision XT v.17.0 (Noldus).

#### Accelerated Rotarod Test

Motor coordination and motor learning were assessed using the accelerated rotarod test. The experimental procedure, performed over three consecutive days, consisted of two days of training followed by one test session on the third day (protocol adapted from [63]). The test was conducted using a computerized four-lane rotarod apparatus (Panlab, Harvard Apparatus), featuring a rotating cylinder (3 cm diameter) with independently controlled lanes, such that up to four mice could be tested simultaneously.

On the first day, mice underwent an acclimation phase consisting of a single 30 s trial on the rod rotating at a constant speed of 4 rpm. Subsequently, in each session, the rod acceleration was programmed to increase Linearly from 4 to 40 rpm over a 300 s period. Each session included four trials per mouse, separated by 5 min inter-trial intervals. The following behavioral parameters were measured for each trial: latency to fall (time in seconds spent on the rod) and the best performance across sessions, defined as the longest latency to fall on the final test day.

#### Power analysis, sample size, randomization, blinding

The sample number per group and experiment was determined by power analysis (G*Power software, 3.1.9.7v) using a power of 0.8 and errors of 0.05; standard deviations of all groups were obtained from previous publications with similar experiments.

Randomization was done with a random-number table to decide how mice from the same litter would be randomly assigned to the different groups.

Researchers were blinded to the animal group; un-blinding occurred after analysis.

The experimental units for each experiment are described in detail in Figure legends.

#### Statistical analysis

Analyses were performed using Prism8.01 (GraphPad). Data were checked for normality using the Shapiro–Wilk and D’Agostino-Pearson tests. Data from two groups (i.e., Sham/Casp3, Saline/6OHDA, WT/Tg2576, Tg Sham/Tg Casp3) were analyzed with Two-tailed parametric (unpaired *t*-test or Welch’s *t*-test) or non-parametric Mann–Whitney tests according to normality.

Sholl analysis data, PPR, I/O curves, mean exploration time during the training and test phases of the NOR test, mean exploration time during the familiarization and test phases of the SOR test, and mean latency to fall across sessions in the rotarod test were analyzed by Two-way Repeated-Measures (RM) ANOVA, using distance from soma, interval, stimulus intensity, object category (left *vs* right and novel *vs* familiar), test sessions (S1-4), object category (DO *vs* NDO), and number of sessions (S1-12) as repeated values, respectively. Post-hoc tests were performed using Sidak’s or Tukey’s multiple comparison tests.

Data from three or four groups were analyzed by One-Way ANOVA followed by Tukey’s multiple comparison test, or with Kruskal–Wallis followed by Dunn’s multiple comparison test.

For the differential expression analysis of transcriptomics expression, statistical significance was determined using the Wald test [64]. Cut offs (*p* < 0.050) were used for *p* values and for *p* values adjusted with the False Discovery Rate (FDR) methods of Benjamini–Hochberg and Benjamini-Yekutieli (FDR B&H and FDR B&Y, respectively).

See Figure legends for more details. *p* ≤ 0.05 indicates statistical significance. In box-and-whisker plots, the central line denotes the median, edges are upper and lower quartiles, whiskers show minimum and maximum values and points are individual experiments. In the violin plots used for spine density, the width of the plot at any given point corresponds to the frequency of data points at that value. The volcano plot for the transcriptomic analysis shows scattered individual data points. All other data are presented as mean ± s.e.m..

## Results

### Lesion of midbrain DA and 5-HT nuclei induces hippocampal NLRP3-mediated microglia response in C57BL/6N mice

**Fig. 1:**
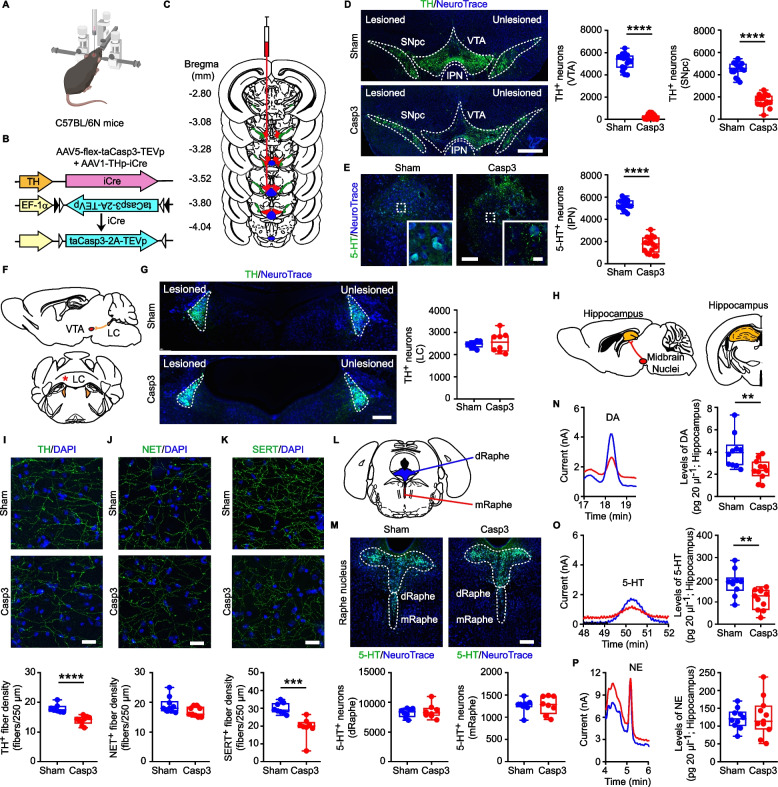
Validation of midbrain lesion in Casp3 mice. **A-B** C57BL/6N mice were unilaterally infused in the midbrain with a dual-AAV approach. The double-floxed AAV5-flex-taCasp3-TEVp, driven by the EF-1α promoter, comprises the inverted taCasp3-TEVp. Expression of iCre-recombinase under the TH promoter (AAV1-THp-iCre) determines recombination resulting in the active orientation of taCasp3-TEVp. **C** Rostro-caudal extent of regions for cell counts. The evaluated midbrain nuclei are VTA (red), SNpc (green), IPN (blue). **D** Immunofluorescence of TH^+^ neurons, counterstained with NeuroTrace, in Sham and Casp3 mice (scale: 500 µm) and stereological TH^+^ cell count in the ipsilateral VTA (*n* = 16 mice/group. Mann–Whitney test: ****p < 0.0001) and SNpc (*n* = 16 mice/group. Unpaired *t*-test: ****p < 0.0001). **E** Images of IPN stained with 5-HT and NeuroTrace (scale: 100 μm; inset: 10 µm), and stereological 5-HT^+^ cell count (*n* = 16 mice/group. Unpaired *t*-test: ****p < 0.0001). **F** Scheme of LC-VTA connectivity (sagittal section) and of LC (coronal section). The * indicates the analyzed hemisphere. **G** Confocal images of LC TH^+^ neurons (counterstained with NeuroTrace, scale: 200 µm) and plot of TH^+^ cell counts (*n* = 8 mice/group). **H** Scheme of VTA-hippocampus connectivity (sagittal section) and of hippocampus (coronal section). **I**-**K** Confocal images and plots showing **I** TH^+^ (Sham: *n* = 7, Casp3: *n* = 9 mice. Unpaired *t*-test: ****p < 0.0001), **J** NET^+^ (Sham: *n* = 10; Casp3: *n* = 9 mice) and **K** SERT^+^ (*n* = 8 mice/group. Unpaired *t*-test: ***p = 0.0003) hippocampal fiber density (expressed as fibers per 250 μm; scale: 20 µm). Nuclei are counterstained with DAPI. **L** Schematic representation of dorsal (dRaphe; blue) and medial raphe (mRaphe; red; coronal section). **M** Confocal images of dRaphe and mRaphe stained with 5-HT in Sham and Casp3 mice (counterstained with NeuroTrace; scale: 200 µm) and plots showing 5-HT^+^ neuron counts (*n* = 8 mice/group). **N-P** Representative chromatogram segments and plots of monoamine hippocampal levels (Sham: *n* = 10, Casp3: *n* = 11 mice; *DA*: Unpaired *t*-test: **p = 0.007; *5-HT*: Unpaired *t*-test: **p = 0.004). [Figure created using BioRender.com]

To investigate the involvement of DA and 5-HT in hippocampal function, we induced a midbrain lesion in the C57BL/6N mouse strain. Specifically, we lesioned the dopaminergic VTA and the medial portion of the SNpc, and the serotonergic IPN, known to send monoaminergic inputs to the hippocampus [38, 39, 65–67]. To target these nuclei simultaneously with a single tool, we took advantage of the ectopic expression of the TH promoter. Indeed, although the TH protein is selectively expressed in dopaminergic neurons of the VTA and SNpc, the TH promoter is also ectopically expressed in the IPN [68–70]. Therefore, we used a combined viral approach to unilaterally co-infuse in the midbrain (i) an AAV expressing an improved Cre-recombinase under the TH promoter (AAV1-THp-iCre), together with (ii) an AAV containing the Cre-recombinase-inducible floxed Caspase-3 (AAV5-flex-taCasp3-TEVp) gene, in order to overexpress Casp3, an executioner caspase in apoptosis [71], following recombination driven by the TH promoter [68, 72]; thereafter, Casp3 mice; Fig. 1A,B). As controls, mice were injected only with the AAV1-THp-iCre virus (thereafter, Sham mice). We confirmed the efficacy of the Cre-mediated recombination by detecting increased levels of full-length Casp3 protein in the ipsilateral midbrain of Casp3 mice compared to Sham controls, 72 h after injection (Supplemental Fig. 1A). Additionally, the ectopic TH promoter-driven AAV expression was validated by co-infusing the AAV1-THp-iCre virus with the reporter virus AAV5-EF1α-DIO-eYFP into the midbrain, resulting in the expression of the eYFP fluorophore in the VTA, SNpc and IPN (Supplemental Fig. 1B, C). To exclude the potential off-target effect of the TH promoter-driven AAV, we also verified the absence of eYFP expression in non-midbrain regions relevant to AD, such as the Raphe nuclei and LC (Supplemental Fig. 1D, E).

In Casp3 and Sham mice we next examined the midbrain rostro-caudally to confirm the lesion in the VTA, SNpc and IPN (Fig. 1C). The stereological counting of TH^+^ neurons in the ipsilateral VTA and SNpc revealed a significant decrease in Casp3 compared to Sham mice 1 month after the lesion (Fig. 1D). This was accompanied by reduced total TH protein levels in the midbrain (Supplemental Fig. 1F). Consistent with the ectopic activity of the TH promoter in the IPN, 5-HT^+^ neurons were also significantly reduced in Casp3 mice (Fig. 1E). Since the midbrain receives TH^+^ fibers from the LC, we also investigated whether the viral infusion could lead to the retrograde loss of TH^+^ LC neurons (Fig. 1F). Yet, no changes were observed in the number of LC TH^+^ neurons in Casp3 mice (Fig. 1G).

To confirm the reduction of midbrain monoamine inputs to the hippocampus of Casp3 mice, 1 month after surgery we analyzed the dopaminergic and serotonergic innervations in the dorsal hippocampus (Fig. 1H). The density of hippocampal TH^+^ fibers was significantly reduced in Casp3 mice (Fig. 1I). This was not due to damage to the TH^+^ innervation from the LC, as the density of NET^+^ fibers was unaltered (Fig. 1J). Consistent with the significant loss of IPN 5-HT^+^ neurons, in Casp3 mice there was also a reduction in the density of hippocampal serotonergic fibers, labelled for the 5-HT transporter (SERT; Fig. 1K). Of note, the stereological cell counting of 5-HT^+^ neurons in both the dorsal and medial Raphe showed no changes in Casp3 mice (Fig. 1L,M), confirming that the reduction of hippocampal SERT^+^ fibers is solely due to the IPN lesion.

In Line with the loss of dopaminergic and serotonergic innervation in the hippocampus, HPLC analysis of total hippocampal tissue confirmed a drop of both DA and 5-HT and their relative metabolites (DOPAC, HVA and 5-HIAA) in Casp3 mice (Fig. 1N,O; Supplemental Table 1). No changes in NE and MOPEG levels were observed (Fig. 1P; Supplemental Table 1). Thus, the midbrain lesion in Casp3 mice results in the combined depletion of both DA and 5-HT in the hippocampus.

Consistent with the known role of DA and 5-HT in modulating hippocampal-dependent memory [36, 73, 74], Casp3 mice exhibited significant impairments in object recognition and spatial memory, as assessed by the NOR and SOR tests, respectively (Supplemental Fig. 2A-C). Casp3 mice also displayed anxiety-like behavior—evidenced by reduced time spent in the center and increased time in the periphery of the open field arena—as well as hyperlocomotion, reflected by a greater total distance traveled (Supplemental Fig. 2D, E). Notably, Casp3 mice showed no motor coordination deficits, in the accelerated rotarod test compared to Sham mice (Supplemental Fig. 2F).

**Fig. 2:**
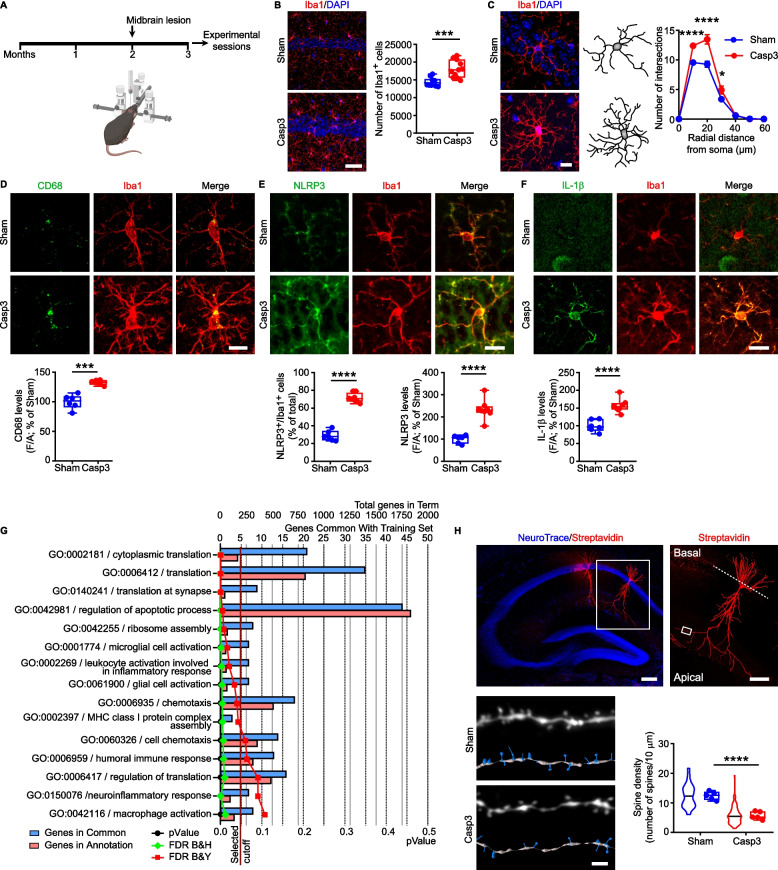
Midbrain lesion induces NLRP3-dependent microglia response in the hippocampus of Casp3 mice. **A** Experimental workflow: mice were examined 1 month after lesion. **B** Images and plot showing hippocampal Iba1^+^ cell count in male Sham and Casp3 mice (Sham: *n* = 10; Casp3: *n* = 11 mice; Unpaired *t*-test: ***p = 0.0003). Nuclei are counterstained with DAPI (scale: 50 µm). **C** Representative images and 3D-reconstruction of microglia (scale: 10 μm) in male mice. The graph shows microglia Sholl analysis, depicting number of intersections at different distances from soma (Sham: *n* = 10; Casp3: *n* = 7 mice; Two-way RM-ANOVA: *Intersections*: interaction F_6,90_ = 15.58, *p* < 0.0001; distance F_6,90_ = 579.6, *p* < 0.0001; lesion F_1,15_ = 16.68, *p* = 0.001; Sham *vs* Casp3: ****p < 0.0001 at 10–20 µm; *p = 0.017 at 30 µm, analyzed with Sidak’s multiple comparisons test). **D** Representative images and plot showing CD68 immunostaining intensity in microglia of male mice (scale: 10 µm; *n* = 6 mice/group; Welch’s *t*-test: ***p = 0.0007). **E** Confocal images of NLRP3 and Iba1 immunostaining in male mice (scale: 15 µm). The plots show: % of NLRP3^+/^Iba1^+^ cells (Sham: *n* = 6; Casp3: *n* = 8 mice. Unpaired *t*-test: ****p < 0.0001) and NLRP3 levels (Unpaired *t*-test: ****p < 0.0001). **F** Confocal images and plot of hippocampal IL-1β levels (scale: 15 µm) in microglia from male mice (Sham: *n* = 6; Casp3: *n* = 8 mice; Unpaired *t*-test: ****p < 0.0001). **G** Functional enrichment analysis of DEGs (*p* < 0.05) identified in hippocampal microglia gene expression profile of Casp3 compared to Sham CX_3_CR-1^GFP^ female mice. The chart displays the most significant functional enrichment terms for the gene ontology (GO) biological process. The analysis was performed with the ToppGene suite. **H** *Top*, confocal images of biocytin-filled CA1 pyramidal neurons (scale: *left* 250 µm; *right* 100 µm). *Bottom left*, representative images and 3D reconstruction of apical dendritic fragments in the CA1 of Sham and Casp3 mice (scale: *left* 2 µm). *Bottom right,* analysis of spine density in hippocampal pyramidal neurons. Violin plots represent quantification per dendrite and dots represent average data per animal (at least 18 dendritic fragments from 4 cells/5 mice were used per group; Unpaired *t*-test: ****p < 0.0001). [Figure created using BioRender.com]

To investigate whether the midbrain lesion could induce the insurgence of inflammatory events in the hippocampus, we examined morphological and functional changes in microglia and astrocytes, 1 month after the midbrain lesion (Fig. 2A). The stereological cell count in the dorsal hippocampus revealed an increase in the number of Iba1^+^ microglia in Casp3 mice (Fig. 2B). In contrast, the number of GFAP^+^ astrocytes remained unchanged following lesion (Supplemental Fig. 3A). To further investigate phenotypical changes in microglia, we analyzed their morphology using Sholl analysis. We observed a greater complexity of microglia processes in Casp3 mice, including increased number of intersections, nodes and endings, and increased length of processes (Fig. 2C, Supplemental Fig. 3B). The somatic perimeter and area of microglia were similar between groups (Supplemental Fig. 3C). Functional marker analysis showed a higher CD68 immunoreactivity in the cell body of Iba1^+^ cells from Casp3 mice (Fig. 2D), overall suggesting altered microglia.

**Fig. 3:**
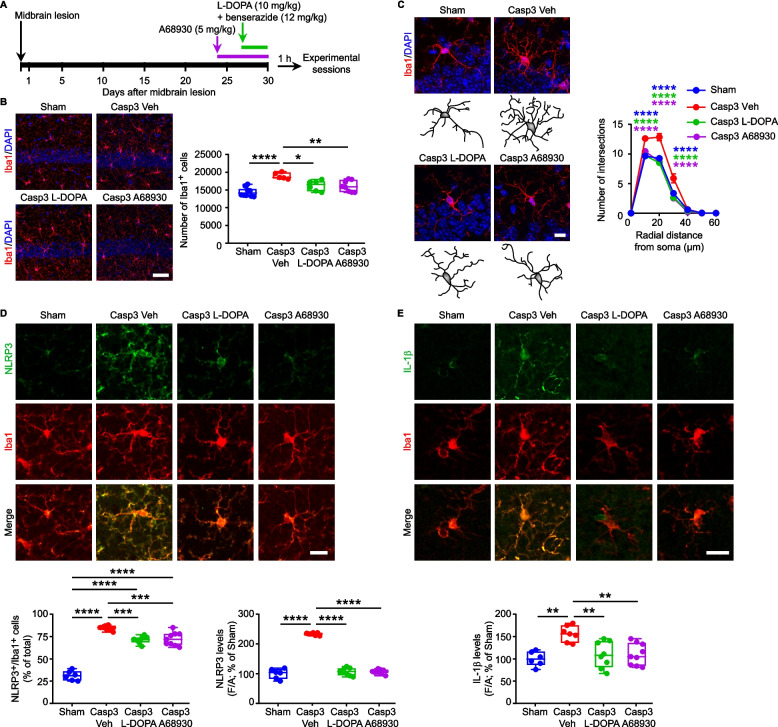
Dopaminergic treatments suppress microglia-mediated neuroinflammation in the Casp3 hippocampus. **A** Experimental procedure for the sub-chronic treatments of saline (Veh), L-DOPA (10 mg/kg + benserazide 12 mg/kg; 4 days; i.p) or A68930 (5 mg/kg; 7 days; i.p.) in Casp3 mice. Animals were sacrificed 1 h after the last treatment. **B** Confocal images and plot showing Iba1^+^ numbers in the hippocampus (scale: 50 µm) of Sham mice and of Casp3 mice treated with Veh or L-DOPA or A68930 (Sham: *n* = 10, Casp3 Veh: *n* = 5, Casp3 L-DOPA: *n* = 6, Casp3 A68930: *n* = 8 mice; One-way ANOVA: F_3, 25_ = 12.92; *p* < 0.0001; Sham *vs* Casp Veh: ****p < 0.0001; Casp3 Veh *vs* Casp3 L-DOPA: *p = 0.012, Casp3 Veh *vs* Casp3 A68930: **p = 0.004, with Tukey’s multiple comparisons test). Nuclei are counterstained with DAPI. **C** Representative confocal images and 3D-reconstruction of microglia in Sham and Casp3 mice treated with Veh, L-DOPA or A68930 (scale: 10 μm) and intersection analysis (Sham: *n* = 10; Casp3 Veh: *n* = 5, Casp3 L-DOPA: *n* = 7, Casp3 A68930: *n* = 6 mice. Two-way RM-ANOVA: *Intersections*: interaction F_18,144_ = 11.59, *p* < 0.0001; distance F_6,144_ = 1530, *p* < 0.0001; treatment F_3,24_ = 20.31, *p* < 0.0001; Sham *vs* Casp3 Veh (blue): ****p < 0.0001 at 10–30 µm; Casp3 Veh *vs* Casp3 L-DOPA (green): ****p < 0.0001 at 10–30 µm; Casp3 Veh *vs* Casp3 A68930 (purple): ****p < 0.0001 at 10–30 µm, analyzed with Tukey’s multiple comparisons test). **D** Confocal images of NLRP3 and Iba1 immunostaining (scale: 15 µm). The plots show: the % of NLRP3^+^/Iba1^+^ cells (Sham: *n* = 6; Casp3 Veh: *n* = 7, Casp3 L-DOPA: *n* = 8, Casp3 A68930: *n* = 9 mice; One-Way ANOVA: F_3,26_ = 121.4; *p* < 0.0001. Sham *vs* Casp3 Veh: ****p < 0.0001, Sham *vs* Casp3 L-DOPA: ****p < 0.0001, Sham *vs* Casp3 A68930: ****p < 0.0001, Casp3 Veh *vs* Casp3 L-DOPA: ***p = 0.0003, Casp3 Veh *vs* Casp3 A68930: ***p = 0.0006, with Tukey’s) and NLRP3 levels (One-Way ANOVA: F_3,26_ = 254; *p* < 0.0001. Sham *vs* Casp3 Veh: ****p < 0.0001, Casp3 Veh *vs* Casp3 L-DOPA: ****p < 0.0001, Casp3 Veh *vs* Casp3 A68930: ****p < 0.0001, with Tukey’s). **E** Confocal images of IL-1β and Iba1 (scale: 15 µm) and plot of IL-1β levels (Sham: *n* = 6; Casp3 Veh: *n* = 7, Casp3 L-DOPA: *n* = 8, Casp3 A68930: *n* = 9 mice; One-Way ANOVA: F_3,26_ = 8.224; *p* = 0.0005. Sham *vs* Casp3 Veh: **p = 0.001, Casp3 Veh *vs* Casp3 L-DOPA: **p = 0.003, Casp3 Veh *vs* Casp3 A68930: **p = 0.003, with Tukey’s)

One of the key contributors in the development of neuroinflammation is the NLRP3 inflammasome, responsible for the secretion of pro-inflammatory cytokines such as IL-1β and IL-18 [75, 76]. Therefore, we examined the number of Iba1^+^ cells expressing NLRP3 (NLRP3^+^/Iba1^+^) and the levels of NLRP3 and IL-1β. Cell counting revealed a significant increase in the percentage of NLRP3^+^/Iba1^+^ cells in Casp3 compared to Sham mice (Fig. 2E, *left*). Moreover, we found increased NLRP3 immunoreactivity (Fig. 2E, *right*), associated with higher IL-1β levels (Fig. 2F), proving a pro-inflammatory profile. Notably, similar microglial alterations were also observed in female Casp3 compared to sex-matched Sham mice (Supplemental Fig. 3D-J), indicating that the midbrain lesion induces microglia-driven hippocampal neuroinflammation irrespective of sex.

To assess the transcriptional signatures associated with microglia reactivity, we performed mRNA sequencing on FACS-sorted hippocampal microglia from Sham and Casp3-lesioned CX_3_CR-1^GFP^ mice at 1 month post-lesion (Supplementary Fig. 4A-D). Among the 19117 genes detected, six Differentially Expressed Genes (DEGs) reached significance after FDR correction (FDR < 0.05), and 381 met a nominal threshold (*p* < 0,05), as illustrated in the volcano plot (Supplementary Fig. 4E). These included 138 up- and 243 down-regulated genes in Casp3 mice relative to Sham microglia, with fold changes and associated statistics detailed in Supplementary Table 2.

We next examined the full set of DEGs using ToppGene functional annotation to identify perturbed biological processes. The top enriched Gene Ontology (GO) terms within the “Biological Process” category are shown in Fig. 2G, with the complete list in Supplementary Table 3. Consistent with a shift toward a reactive phenotype, Casp3 microglia exhibited significant enrichment for terms related to microglial cell activation (GO:0001774), chemotaxis (GO:0006935), and neuroinflammatory response (GO:0150076). Additional enriched processes included apoptotic regulation (GO:0042981) and translational machinery dynamics such as cytoplasmic translation (GO:0002181), synaptic translation (GO:0140241) and ribosome assembly (GO:0042255).

Collectively, these results prove that the combined depletion of both midbrain DA and 5-HT in Casp3 mice triggers hippocampal neuroinflammation *via* microglia activation and NLRP3 pathway induction.

Given the well-established role of monoamines in modulating hippocampal function, we next asked whether this neuroinflammatory environment compromises neuronal dendritic structure. Morphological analysis of biocytin‑filled CA1 pyramidal neurons revealed a significant reduction in distal apical spine density in Casp3 mice relative to Sham controls (Fig. 2H). To determine whether this structural alteration translated into a functional deficit, we assessed LTP at CA3–CA1 synapses. LTP appeared unaltered in Casp3 slices (Supplemental Fig. 5A), suggesting that elevated IL‑1β levels might mask a synaptic deficit, an interpretation consistent with prior reports indicating that IL‑1β can enhance LTP [77]. Indeed, pre‑incubation with the IL‑1β receptor antagonist (IL‑1β‑Ra) unmasked a clear LTP impairment in Casp3 mice (Supplemental Fig. 5A), despite unchanged glutamate release probability (PPR) and Input/Output (I/O) curves (Supplemental Fig. 5B).

To prove that the hippocampal microglia response requires the loss of both DA and 5-HT, we examined the striatum, which receives robust dopaminergic but negligible IPN‑derived serotonergic innervation [78]. Retrograde labelling confirmed that the dorsal and ventral striatum (NAc core/shell) do not receive inputs from IPN neurons (Supplemental Fig. 6A-D). The analysis of TH, DAT and SERT markers confirmed that the dopaminergic innervation in the dorsal striatum and NAc core/shell is impaired in Casp3 mice (Supplemental Fig. 6E,F), while the serotonergic fibers are intact (Supplemental Fig. 6G,H). In contrast to what we observed in the hippocampus, there were no changes in Iba1^+^ cell number and morphology in the dorsal striatum (Supplemental Fig. 7A-E), NAc core (Supplemental Fig. 7F-J) or NAc shell (Supplemental Fig. 7K-O), suggesting that the loss of DA alone is insufficient to induce microglia reactivity in our model.

As an additional confirmation, we examined the hippocampus of mice infused in the midbrain with the catecholaminergic neurotoxin 6OHDA that induces selective loss of VTA and SNpc dopaminergic neurons but does not affect IPN 5-HT^+^ neurons (Supplemental Fig. 8A-C). Analysis of microglia in the hippocampus of 6OHDA mice showed no differences in cell number and morphology compared to saline-injected mice (Supplemental Fig. 8D-G). In line with these results, no changes were detected in the levels of CD68, NLRP3 or IL-1β in Iba1^+^ cells of 6OHDA mice (Supplemental Fig. 8H-J).

Similarly, to assess whether the selective loss of the serotonergic component alone is sufficient to elicit hippocampal neuroinflammation, a separate cohort of mice received targeted IPN injections (Casp3^IPN^ mice) of the AAV1-THp-iCre + AAV5-flex-taCasp3-TEVp mix, selectively lesioning 5-HT^+^ neurons while sparing midbrain dopaminergic neurons (Supplemental Fig. 9A-C). Compared with Sham-injected controls (Sham^IPN^), Casp3^IPN^ mice showed a selective reduction of hippocampal SERT^+^ fibers and preserved TH^+^ innervation (Supplemental Fig. 9D, E). Notably, serotonergic denervation alone failed to induce microglia reactivity, as evidenced by unchanged microglia number, morphology (Supplemental Fig. 9F-I) and levels of CD68, NLRP3, or IL-1β immunoreactivity in Iba1^+^ cells (Supplemental Fig. 9J-L), arguing against a pro-inflammatory microglia response.

Taken together, these findings demonstrate that only the combined loss of midbrain DA and 5‑HT innervation synergistically drives hippocampal neuroinflammation, dendritic spine loss, and associated deficits in synaptic plasticity, key pathological features contributing to memory impairment in AD.

### Boosting of DA or 5-HT signaling rescues hippocampal NLRP3-mediated neuroinflammation in Casp3 mice

DA and 5-HT receptors are expressed in both microglia and astrocytes. Indeed, both monoamines negatively regulate glial cell reactivity by inhibiting the NLRP3 inflammasome and the subsequent release of pro-inflammatory cytokines [14, 15, 79, 80].

**Fig. 4:**
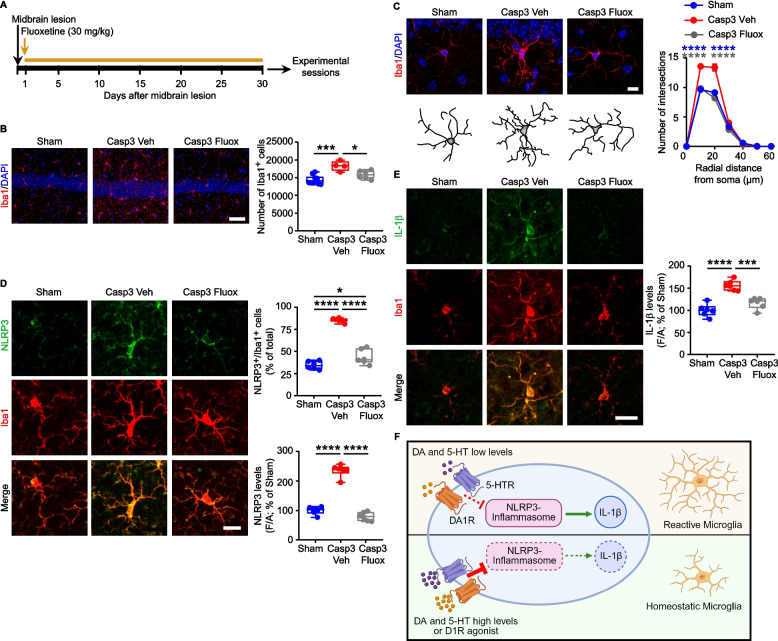
Fluoxetine prevents microglia-mediated neuroinflammation in the Casp3 hippocampus. **A** Experimental procedure for chronic treatment with or without fluoxetine in drinking water (30 mg/kg; 30 days) in Casp3 mice. **B** Confocal images and plot showing stereological cell count of hippocampal Iba1^+^ cells (scale: 50 µm) in Sham mice and in Casp3 mice drinking normal water (Veh) or water containing fluoxetine (Fluox; Sham: *n* = 10, Casp3 Veh: *n* = 4, Casp3 Fluox: *n* = 6 mice; One-way ANOVA: F_2, 17_ = 14.05; *p* = 0.0003; Sham *vs* Casp Veh: ***p = 0.0002; Casp3 Veh *vs* Casp3 Fluox: *p = 0.018, with Tukey’s multiple comparisons test). Nuclei are counterstained with DAPI. **C** Representative images and 3D-reconstruction of microglia in Sham and Casp3 mice treated with Veh or Fluox (scale: 10 μm). The graph shows number of intersections (Sham: *n* = 10, Casp3 Veh: *n* = 4, Casp3 Fluox: *n* = 5 mice. Two-way RM-ANOVA: interaction F_12,96_ = 25.84, *p* < 0.0001; distance F_6,96_ = 1559, *p* < 0.0001; treatment F_2,16_ = 33.80, *p* < 0.0001; Sham *vs* Casp3 Veh (blue): ****p < 0.0001 at 10–20 µm; Casp3 Veh *vs* Casp3 Fluox (grey): ****p < 0.0001 at 10–20 µm, analyzed with Tukey’s multiple comparisons test). **D** Confocal images of NLRP3 and Iba1 in the hippocampus (scale: 15 µm). The plot shows: the % of NLRP3^+^/Iba1^+^ cells (*n* = 6 mice/group; One-way ANOVA: F_2,15_ = 137; *p* < 0.0001; Sham *vs* Casp Veh: ****p < 0.0001; Casp3 Veh *vs* Casp3 Fluox: ****p < 0.0001, Sham *vs* Casp3 Fluox: *p = 0.034; with Tukey’s multiple comparisons test) and NLRP3 levels (One-way ANOVA: F_2,15_ = 157.3; *p* < 0.0001; Sham *vs* Casp Veh: ****p < 0.0001; Casp3 Veh *vs* Casp3 Fluox: ****p < 0.0001; with Tukey’s multiple comparisons test). **E** Confocal images of IL-1β and Iba1 (scale: 15 µm) and plot of IL-1β levels (*n* = 6 mice/group; One-way ANOVA: F_2,15_ = 30.09; *p* < 0.0001; Sham *vs* Casp Veh: ****p < 0.0001; Casp3 Veh *vs* Casp3 Fluox: ***p = 0.0002; with Tukey’s multiple comparisons test). **F** Schematic representation of DA and 5-HT role in microglia. Deprivation of both monoamines triggers NLRP3 inflammasome activation in microglia, promoting IL-1β release. Intervention with dopaminergic (L-DOPA or A68930) or serotonergic (Fluox) drugs inhibits NLRP3-mediated microglia reactivity, reducing IL-1β release. [Figure created using BioRender.com]

We thus treated Casp3 mice with dopaminergic or serotonergic drugs to attenuate hippocampal inflammation (Figs. 3 and 4). Sub-chronic administration of the DA precursor L-DOPA (4 days) or the selective D1 receptor agonist A68930 (7 days) reduced the microglial pro-inflammatory response in Casp3 mice compared to vehicle-treated controls, fully restoring both microglia numbers and morphology to levels comparable to Sham mice (Fig. 3A-C; Supplemental Fig. 10A,B). Both drugs also reduced the percentage of NLRP3^+^/Iba1^+^ cells and the levels of NLRP3 and IL-1β compared to vehicle-treated Casp3 mice (Fig. 3D,E).

Similarly, the chronic treatment of Casp3 mice with the selective 5-HT reuptake inhibitor (SSRI) fluoxetine, started one day after lesioning and continued for 30 days (Fig. 4A), prevented microglia reactivity as shown by the reduced number and morphological complexity of Iba1^+^ cells compared to vehicle-treated Casp3 mice, reaching those of Sham mice (Fig. 4B,C; Supplemental Fig. 10C,D). Fluoxetine also attenuated the activation of the NLRP3 pathway, as demonstrated by the lower percentage of NLRP3^+^/Iba1^+^ cells and the lower levels of NLRP3 and IL-1β compared to vehicle-treated Casp3 mice (Fig. 4D,E). Collectively, these results indicate that both dopaminergic and serotonergic treatments can protect Casp3 mice from NLRP3-mediated neuroinflammation (Fig. 4F).

### Midbrain lesion in the Tg2576 mouse model worsens hippocampal microglia reactivity

**Fig. 5:**
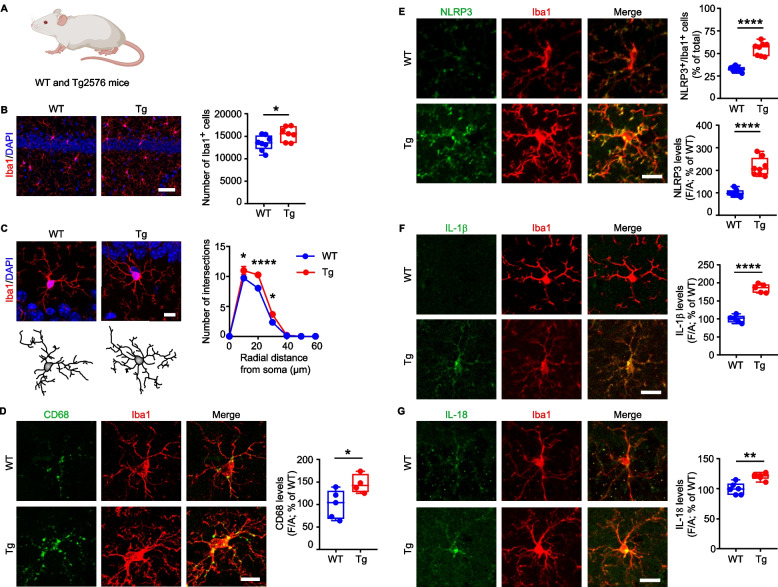
Mild hippocampal microglia reactivity in 7-month-old naïve Tg2576 mice. **A** Evaluation of hippocampal neuroinflammatory processes in wild-type (WT) mice and in naïve pre-plaque Tg2576 (Tg) mice at 7 months of age. **B** Confocal images and plot showing cell count of Iba1^+^ cells in WT and Tg hippocampi (scale: 50 µm. WT: *n* = 8, Tg: *n* = 7 mice. Unpaired *t*-test: *p = 0.031). Nuclei are counterstained with DAPI. **C** Representative images and 3D-reconstruction of microglia (scale: 10 μm). The graph shows Sholl analysis of intersections (*n* = 5 mice/group; Two-way RM-ANOVA: interaction F_6,48_ = 4.832, *p* = 0.0006; distance F_6,48_ = 516.1, *p* < 0.0001; genotype F_1,8_ = 11.20, *p* = 0.010; WT *vs* Tg2576: *p = 0.041 at 10 µm; ****p < 0.0001 at 20 µm; *p = 0.023 at 30 µm, analyzed with Sidak’s multiple comparisons test). Nuclei are counterstained with DAPI. **D** Confocal images of CD68 and Iba1 immunostaining (scale: 10 µm), and plot showing CD68 immunostaining intensity in microglia cell body (WT: *n* = 5; Tg2576: *n* = 4 mice. Unpaired *t*-test: *p = 0.042). **E** Confocal images of NLRP3 and Iba1 immunostaining (scale: 15 µm). The plots show: the % of NLRP3^+^/Iba1^+^ cells (*n* = 8 mice/group; Welch’s *t*-test: ****p < 0.0001) and NLRP3 levels (Welch’s *t*-test: ****p < 0.0001). **F** Confocal images of IL-1β and Iba1 immunostaining (scale: 15 µm), and plot showing IL-1β levels in the hippocampus (*n* = 8 mice/group. Unpaired *t*-test: ****p < 0.0001). **G** Confocal images of IL-18 and Iba1 immunostaining (scale: 15 µm), and plot showing hippocampal IL-18 levels (*n* = 6 mice/group. Unpaired *t*-test: **p = 0.001). [Figure created using BioRender.com]

**Fig. 6:**
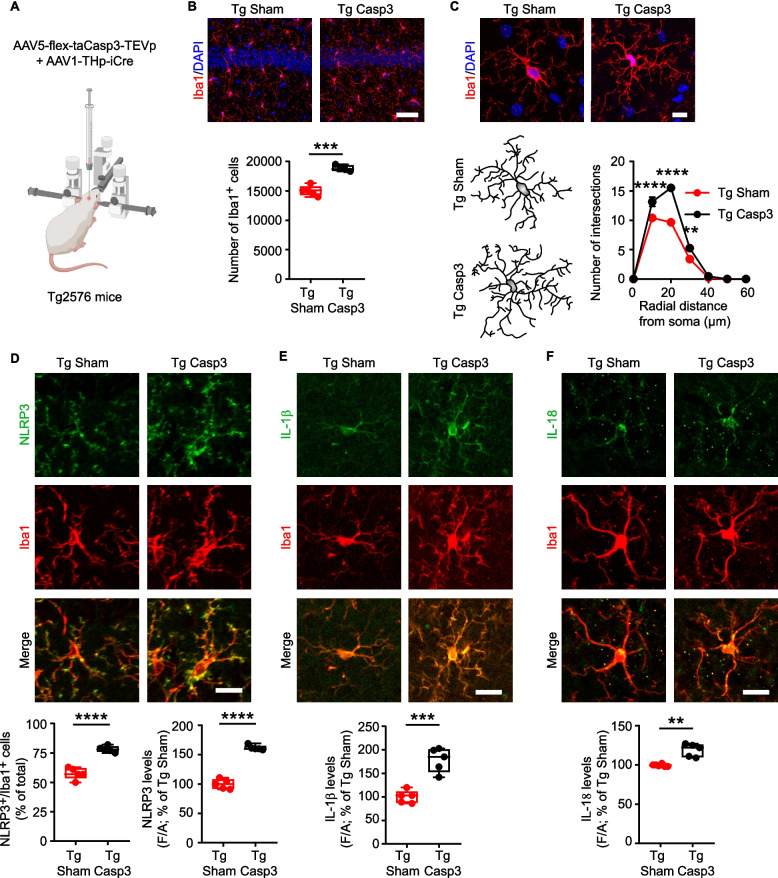
Midbrain lesion exacerbates microglia reactivity in the hippocampus of Tg Casp3 mice. **A** Schematic representation of the midbrain lesion in 6-month-old male Tg mice, using the dual AAV approach. Mice were analyzed 1 month after lesion. **B** Confocal images and plot showing cell count of Iba1^+^ cells in Tg Sham and Tg Casp3 hippocampi (scale: 50 µm. Tg Sham: *n* = 5 and Tg Casp3: *n* = 4 mice; Unpaired *t*-test: ***p = 0.0001). Nuclei are counterstained with DAPI. **C** Representative images and 3D-reconstruction of microglia (scale: 10 μm). The graph shows Sholl analysis of microglia intersections (*n* = 5 mice/group; Two-way RM-ANOVA: interaction F_6,48_ = 16.34, *p* < 0.0001; distance F_6,48_ = 437, *p* < 0.0001; lesion F_1,8_ = 47.90, *p* = 0.0001; Tg Sham *vs* Tg Casp3: ****p < 0.0001 at 10–20 µm; **p = 0.008 at 30 µm, analyzed with Sidak’s multiple comparisons test). **D** Confocal images of NLRP3 and Iba1 (scale: 15 µm). The plots show: % of NLRP3^+^/Iba1^+^ cells (*n* = 5 mice/group; Unpaired *t*-test: ****p < 0.0001) and NLRP3 levels (Unpaired *t*-test: ****p < 0.0001). **E)** Confocal images of IL-1β and Iba1 (scale: 15 µm), and plot showing hippocampal IL-1β levels (*n* = 5 mice/group; Unpaired *t*-test: ***p = 0.0003). **F** Confocal images of IL-18 and Iba1 (scale: 15 µm) and IL-18 levels (Tg Sham: *n* = 6, Tg Casp3: *n* = 5 mice; Unpaired *t*-test: **p = 0.006). [Figure created using BioRender.com]

To evaluate the impact of DA and 5-HT deprivation on hippocampi that are accumulating soluble Aβ, a condition that could mirror the preclinical stage of human AD, we examined the microglial response in naïve Tg2576 mice (Fig. 5) and in Tg2576 mice with midbrain lesion (Tg Casp3, Fig. 6), induced by the same experimental approach as for C57BL/6N mice. We used Tg2576 mice at 7 months of age, an age-point in which we previously described a partial loss of DA neurons in the VTA, leading to reduced hippocampal DA outflow associated with neuronal loss, as well as synaptic and behavioral deficits [45, 50, 56, 60, 81]. Here, we confirmed our previous results showing reduced density of TH^+^ fibers in the dorsal hippocampus of naïve Tg2576 mice (Supplemental Fig. 11A) that cannot be attributed to the loss of TH^+^ neurons in the LC (Supplemental Fig. 11B). In addition, the analysis of hippocampal SERT^+^ fibers and the stereological counting of 5-HT^+^ cells in the IPN revealed that the serotonergic innervation in the dorsal hippocampus of Tg2576 mice remains intact (Supplemental Fig. 11C,D).

To assess whether the hippocampus of naïve Tg2576 mice (Fig. 5A) is affected by microglia reactivity, we examined microglia density and morphological changes. We found a greater number of Iba1^+^ cells in Tg2576 mice (Fig. 5B). These cells had also an increased number of intersections, nodes, endings, and length of their processes, as well as a significant soma enlargement (Fig. 5C, Supplemental Fig. 12A,B) accompanied by enhanced CD68 immunoreactivity (Fig. 5D). Furthermore, we observed a significant increase in the percentage of NLRP3^+^/Iba1^+^ cells in Tg2576 compared to WT mice, along with increased microglia NLRP3, IL-1β and IL-18 immunoreactivity (Fig. 5E-G). Collectively, these data indicate that Tg2576 mice exhibit hippocampal neuroinflammation mediated by microglia through the activation of the NLRP3 inflammasome pathway, which coincides with the loss of DA signaling from the VTA.

To validate our hypothesis that the enhanced loss of midbrain monoamines could aggravate the microglia response, we next examined Tg Casp3 mice, obtained by unilateral co-infusion of the AAV1-THp-iCre and AAV5-flex-taCasp3-TEVp viruses into the left midbrain of 6-month-old Tg2576 mice (Fig. 6A). Consistent with the evidence that DA and 5-HT deficiency triggers microglia-mediated neuroinflammation in the hippocampus of C57BL/6N mice (see Fig. 2), the lesion significantly exacerbated the microglia response in Tg Casp3 compared to Tg Sham mice. Specifically, stereological cell counting and Sholl analysis of Iba1^+^ cells in Tg Casp3 mice 1 month post-lesion revealed an increase in cell numbers and hyper-ramification of microglia processes (Fig. 6B,C; Supplemental Fig. 12C,D). Additionally, Iba1^+^ cells of Tg Casp3 mice showed significantly higher NLRP3, IL-1β and IL-18 immunoreactivity compared to Tg Sham mice (Fig. 6D-F), proving a worsened pro-inflammatory microglial state.

### Midbrain lesion in Tg mice triggers astrocyte reactivity, tau hyperphosphorylation and Aβ plaque deposition

Previous works have shown that reactive microglia can activate astrocytes which, in turn, participate in the inflammatory response by activating the NFκB pathway, leading to the release of Complement 3 (C3). This cascade can further enhance the microglia reactivity impairing Aβ phagocytosis and can promote Aβ and tau pathology in neurons [82–86]. Here, we explored the hypothesis that in Tg Casp3 mice the severe microglia reactivity induced by the midbrain lesion can lead to astrocytic response and, thus, trigger the pathogenic cycle described above.

Before doing so, we examined astrocyte reactivity in the hippocampus of naïve Tg2576 mice. Unlike microglia (see Fig. 5), astrocytes do not appear to be responsive in naïve Tg2576 at 7 months of age, as shown by count of GFAP^+^ cells and the absence of NLRP3 immunoreactivity (Supplemental Fig. 12E,F). Additionally, we examined the microglia-astrocyte crosstalk pathway involving the activation of the IL-18R in astrocytes and the subsequent phosphorylation of NFκB [87]. Consistently with the absence of astrocyte reactivity in the naïve Tg2576 hippocampus, confocal analysis of IL-18R and p-NFκB in astrocytes (immunolabelled by GFAP or S100β) revealed comparable numbers of astrocytes expressing IL-18R and p-NFκB between Tg2576 and WT mice (Supplemental Fig. 12G,H).

**Fig. 7:**
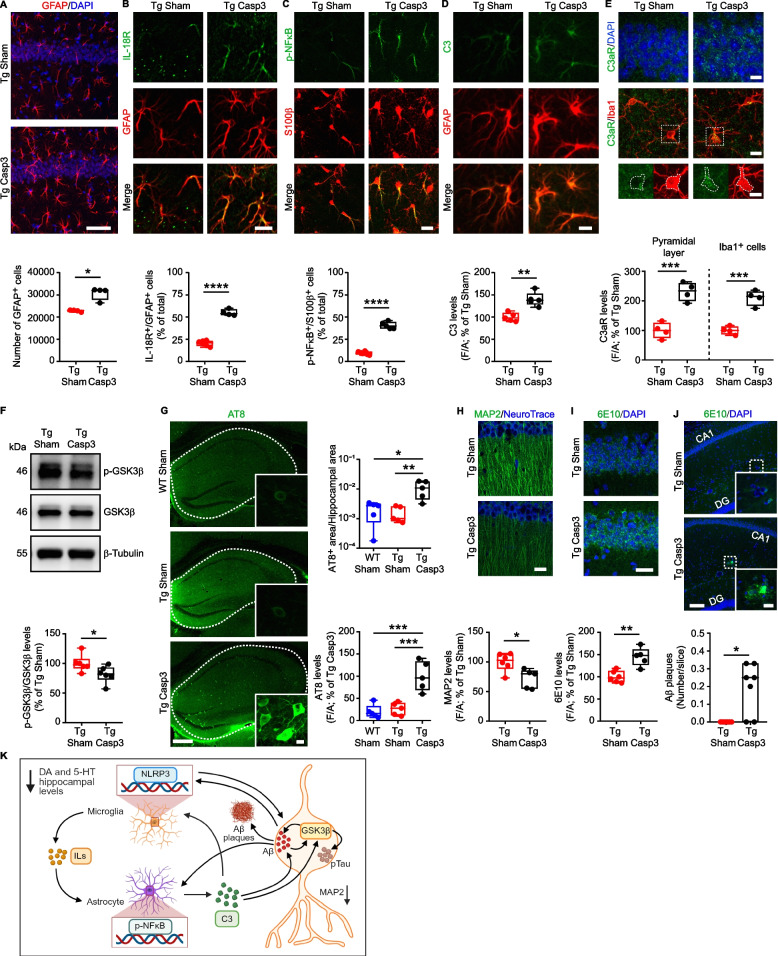
Reactive astrocytes, tau and Aβ pathology in Tg Casp3 mice. **A** Stereological cell count of GFAP^+^ cells in the hippocampus (scale: 50 µm) of Tg Sham and Tg Casp3 male mice (*n* = 4 mice/group; Mann–Whitney test: *p = 0.029). Nuclei are counterstained with DAPI. **B** IL-18R and GFAP immunostaining (scale: 15 µm), and plot showing % of IL-18R^+^/GFAP^+^ cells (Tg Sham: *n* = 6, Tg Casp3: *n* = 4 mice; Unpaired t-test: ****p < 0.0001). **C** p-NFκB and S100β immunostaining (scale: 15 µm) and plot showing % of p-NFκB^+^/S100β^+^ cells in hippocampus (Tg Sham: *n* = 6, Tg Casp3: *n* = 5 mice; Unpaired t-test: ****p < 0.0001). **D** Complement C3 and GFAP immunostaining (scale: 10 µm), and plot showing C3 levels (*n* = 5 mice/group; Unpaired *t*-test: **p = 0.001). **E** Complement C3aR and DAPI (scale: 20 µm) or Iba1 immunostaining (scale: 20 µm, inset 10 µm), and plots showing C3aR levels on neurons (*n* = 4 mice/group; Unpaired *t*-test: ***p = 0.0007) and microglia (Unpaired *t*-test: ***p = 0.0003). **F** Representative western blots and plot showing levels of p-GSK3β/GSK3β (as % of Tg Sham). β-Tubulin was used as loading control (*n* = 6 mice/group; Unpaired *t*-test: *p = 0.048). **G** AT8 immunostaining in WT, Tg Sham and Tg Casp3 hippocampi and plot showing AT8^+^ area on total hippocampal area (scale bar: 250 µm; *n* = 5 mice/group. One-Way ANOVA: F_2,12_ = 8.817; *p* = 0.004. WT Sham *vs* Tg Casp3: *p = 0.011, Tg Sham *vs* Tg Casp3: **p = 0.007, with Tukey’s). The insets and bottom plot show AT8-reactive cells and AT8 levels (scale: 20 µm; *n* = 5 mice/group. One-Way ANOVA: F_2,12_ = 19.27; *p* = 0.0002. WT *vs* Tg Casp3: ***p = 0.0003, Tg Sham *vs* Tg Casp3: ***p = 0.0007, with Tukey’s). **H** MAP2 immunostaining and plot showing MAP2 levels (counterstained with NeuroTrace; scale: 25 µm; Tg Sham: *n* = 6, Tg Casp3: *n* = 5 mice; Unpaired *t*-test: *p = 0.017). **I** 6E10 immunostaining and plot of intracellular Aβ levels (Tg Sham: *n* = 6, Tg Casp3: *n* = 5 mice; Unpaired *t*-test: **p = 0.002). Nuclei are counterstained with DAPI (scale: 50 µm). **J)** Immunostaining for Aβ plaques and Iba1 (scale: 100 µm, inset 20 µm), and plot reporting hippocampal Aβ plaque load (*n* = 7 mice/group; Mann–Whitney test: *p = 0.021). **K** DA and 5-HT loss due to midbrain lesion triggers microglia-astrocyte-neuron crosstalk fostering neuroinflammation and, thus, exacerbating AD pathology in the hippocampus of Tg Casp3 mice. [Figure created using BioRender.com]

We observed a different scenario in Tg Casp3 mice. Specifically, the enhanced NLRP3 inflammasome activity in microglia of Tg Casp3 mice was associated with an increased number of astrocytes and a higher portion of astrocytes expressing IL-18R and p-NFκB (Fig. 7A-C). Furthermore, astrocytes from Tg Casp3 mice showed increased C3 immunoreactivity (Fig. 7D), suggesting a pro-inflammatory activation. Interestingly, the astrocyte response in Tg Casp3 mice was not mediated by the NLRP3-inflammasome pathway (Supplemental Fig. 12I).

To further investigate the astrocyte-neuron and astrocyte-microglia crosstalk in Tg Casp3 mice in response to elevated astrocytic C3, we assessed the C3a receptor (C3aR) levels in hippocampal CA1 pyramidal neurons and microglia. Both cell types exhibited an approximately 2-fold increase in C3aR immunoreactivity (Fig. 7E), which, in the case of microglia, may explain the exacerbated microglial response observed in Tg Casp3 compared to Tg Sham mice (see Fig. 6). The astrocyte-neuron communication *via* the intracellular C3 pathway has been shown to drive Aβ and tau pathology as well as synaptic dysfunction in neurons, potentially through modulation of different kinases, including GSK3β and the Mitogen-Activated Protein Kinases (MAPKs) such as JNK and p38 [82, 83, 88, 89]. We therefore quantified the phosphorylated levels of GSK3β, JNK and p38 and observed a significant reduction of p-GSK3β, indicating an increased GSK3β activity in Tg Casp3 compared to Tg Sham mice (Fig. 7F). Instead, the levels of p-JNK and p-p38 were unchanged in Tg Casp3 mice (Supplemental Fig. 12J). Given the known role of GSK3β in the progression of tau pathology [90, 91], we next evaluated tau hyperphosphorylation (immunolabelled with the AT8 antibody). We found a stronger AT8 immunoreactivity in the hippocampus of Tg Casp3 mice, associated with enhanced intracellular levels of hyperphosphorylated tau (Fig. 7G). Of note, the levels of phosphorylated tau in the hippocampus of 7-month-old Tg Sham mice are similar to those of WT littermates (Fig. 7G), suggesting that midbrain lesion in Tg2576 mice drives secondary tau pathology. The increased activity of GSK3β has also been associated with reduced microtubule stabilization and aggravated Aβ pathology [92–94]. Accordingly, we found a diffuse reduction of the dendritic MAP2 in pyramidal neurons of Tg Casp3 mice, suggestive of pathological changes in dendritic integrity (Fig. 7H), and increased intracellular Aβ levels (Fig. 7I), paralleled by the appearance of precocious Aβ plaque accumulation (Fig. 7J).

### Astrocyte reactivity and tau pathology in Tg Casp3 mice are mitigated by dopaminergic or serotonergic treatment

**Fig. 8:**
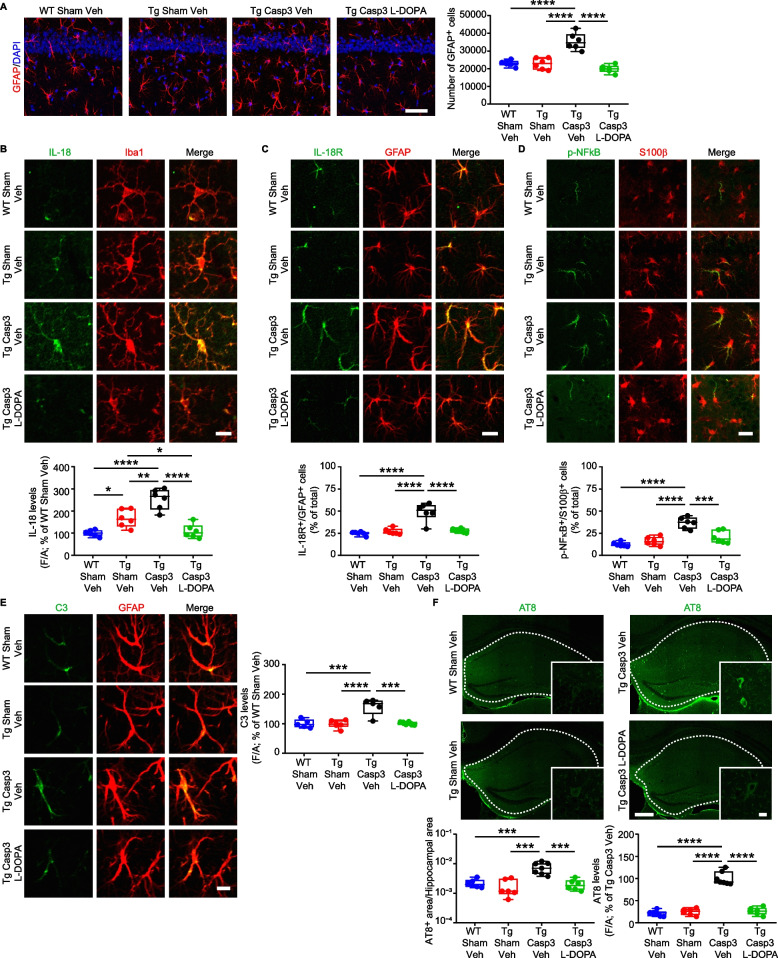
L-DOPA treatment attenuates astrocyte reactivity and tau pathology in Tg Casp3 mice. **A** Representative figures (scale: 50 µm) and plot showing stereological count of GFAP^+^ cells in the hippocampus of WT Sham, Tg Sham and Tg Casp3 mice treated with vehicle (Veh) and Tg Casp3 mice treated with L-DOPA (*n* = 6 mice/group; One-Way ANOVA: F_3,20_ = 28.83; *p* < 0.0001. WT Sham Veh *vs* Tg Casp3 Veh ****p < 0.0001, Tg Sham Veh *vs* Tg Casp3 Veh: ****p < 0.0001, Tg Casp3 Veh *vs* Tg Casp3 L-DOPA: ****p < 0.0001; with Tukey’s). Nuclei are counterstained with DAPI. **B** Confocal images of IL-18 and Iba1 (scale: 15 µm) and plot showing IL-18 levels following Veh or L-DOPA treatment (*n* = 6 mice/group; One-Way ANOVA: F_3,20_ = 24.71; *p* < 0.0001. WT Sham Veh *vs* Tg Sham Veh: *p = 0.018, WT Sham Veh *vs* Tg Casp3 Veh ****p < 0.0001, Tg Sham Veh *vs* Tg Casp3 Veh: **p = 0.002, Tg Sham Veh *vs* Tg Casp3 L-DOPA *p = 0.044, Tg Casp3 Veh *vs* Tg Casp3 L-DOPA: ****p < 0.0001; with Tukey’s). Nuclei are counterstained with DAPI. **C** IL-18R and GFAP immunostaining (scale: 10 µm), and plot showing % of IL-18R^+^/GFAP^+^ cells following Veh or L-DOPA treatment (*n* = 6 mice/group; One-Way ANOVA: F_3,20_ = 23.92; *p* < 0.0001. WT Sham Veh *vs* Tg Casp3 Veh ****p < 0.0001, Tg Sham Veh *vs* Tg Casp3 Veh: ****p < 0.0001, Tg Casp3 Veh *vs* Tg Casp3 L-DOPA: ****p < 0.0001; with Tukey’s). Nuclei are counterstained with DAPI. **D** p-NFκB and S100β immunostaining (scale: 20 µm) and plot showing % of p-NFκB^+^/S100β^+^ cells in the hippocampus following Veh or L-DOPA treatment (*n* = 6 mice/group; One-Way ANOVA: F_3,20_ = 22.92; *p* < 0.0001. WT Sham Veh *vs* Tg Casp3 Veh: ****p < 0.0001, Tg Sham Veh *vs* Tg Casp3 Veh: ****p < 0.0001, Tg Casp3 Veh *vs* Tg Casp3 L-DOPA: ***p = 0.0003; with Tukey’s). **E** Complement C3 and GFAP immunostaining (scale: 10 µm), and plot showing C3 levels following Veh or L-DOPA treatment (WT Sham Veh: *n* = 5, Tg Sham Veh: *n* = 6, Tg Casp3 Veh: *n* = 5, Tg Casp3 L-DOPA: *n* = 6 mice; One-Way ANOVA: F_3,18_ = 15.29; *p* < 0.0001. WT Sham Veh *vs* Tg Casp3 Veh: ***p = 0.0002, Tg Sham Veh *vs* Tg Casp3 Veh: ****p < 0.0001, Tg Casp3 Veh *vs* Tg Casp3 L-DOPA: ***p = 0.0002; with Tukey’s). **F** AT8 immunostaining in WT Sham Veh, Tg Sham Veh, Tg Casp3 Veh and Tg Casp L-DOPA hippocampi and plot showing AT8^+^ area on total hippocampal area (scale bar: 250 µm; WT Sham Veh: *n* = 6, Tg Sham Veh: *n* = 6, Tg Casp3 Veh: *n* = 7, Tg Casp L-DOPA: *n* = 6 mice/group. One-Way ANOVA: F_3,21_ = 14.37; *p* < 0.0001. WT Sham Veh *vs* Tg Casp3 Veh: ***p = 0.0003, Tg Sham Veh *vs* Tg Casp3 Veh: ***p = 0.0001, Tg Casp3 Veh *vs* Tg Casp L-DOPA: ***p = 0.0002, with Tukey’s). The insets and bottom plot show AT8-reactive cells and AT8 levels (scale: 20 µm; One-Way ANOVA: F_3,21_ = 94.83; *p* < 0.0001. WT Sham Veh *vs* Tg Casp3 Veh: ****p < 0.0001, Tg Sham Veh *vs* Tg Casp3 Veh: ****p < 0.0001, Tg Casp3 Veh *vs* Tg Casp L-DOPA: ****p < 0.0001, with Tukey’s)

**Fig. 9:**
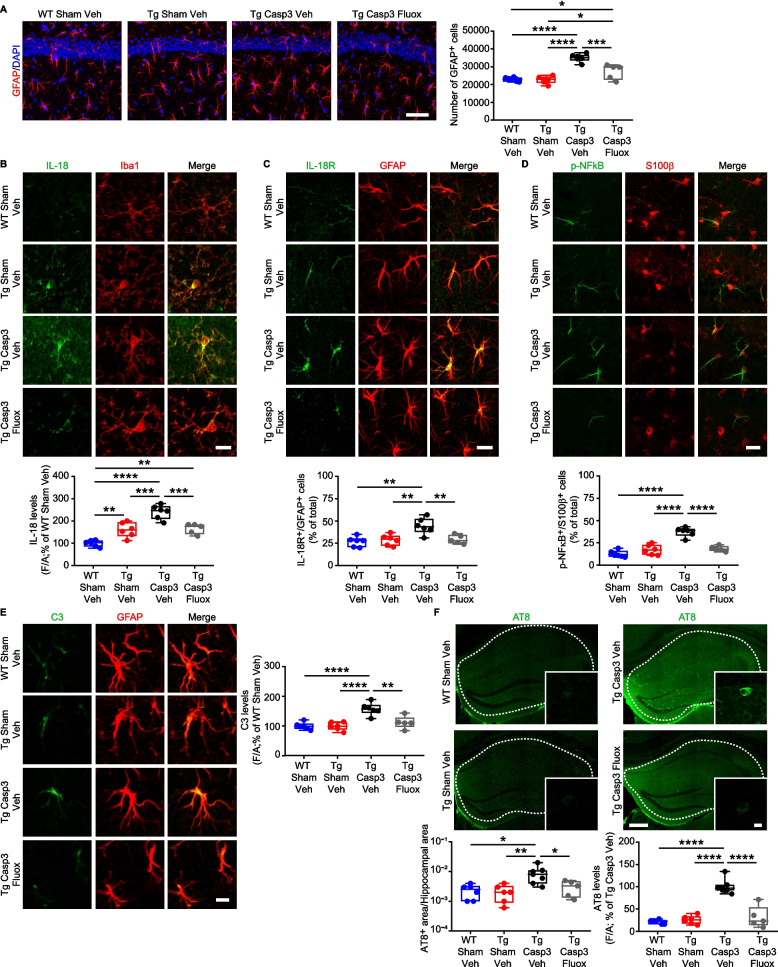
Fluoxetine treatment prevents astrocyte reactivity and tau pathology in Tg Casp3 mice. **A** Representative figures (scale: 50 µm) and plot showing stereological count of GFAP^+^ cells in the hippocampus of WT Sham, Tg Sham and Tg Casp3 mice drinking normal water (Veh) and Tg Casp3 mice drinking water containing fluoxetine (Fluox; WT Sham Veh: *n* = 6, Tg Sham Veh: *n* = 6, Tg Casp3 Veh: *n* = 6, Tg Casp3 Fluox: *n* = 5 mice; One-Way ANOVA: F_3,19_ = 30.85; *p* < 0.0001. WT Sham Veh *vs* Tg Casp3 Veh: ****p < 0.0001, WT Sham Veh *vs* Tg Casp3 Fluox: *p = 0.050, Tg Sham Veh *vs* Tg Casp3 Veh: ****p < 0.0001, Tg Sham Veh *vs* Tg Casp3 Fluox: *p = 0.036, Tg Casp3 Veh *vs* Tg Casp3 Fluox: ***p = 0.0004; with Tukey’s). Nuclei are counterstained with DAPI. **B** Confocal images of IL-18 and Iba1 (scale: 15 µm) and plot showing IL-18 levels following Veh or Fluox treatment (WT Sham Veh: *n* = 6, Tg Sham Veh: *n* = 6, Tg Casp3 Veh: *n* = 6, Tg Casp3 Fluox: *n* = 5 mice; One-Way ANOVA: F_3,19_ = 27.96; *p* < 0.0001. WT Sham Veh *vs* Tg Sham Veh: **p = 0.005, WT Sham Veh *vs* Tg Casp3 Veh: ****p < 0.0001, WT Sham Veh *vs* Tg Casp3 Fluox: **p = 0.004, Tg Sham Veh *vs* Tg Casp3 Veh: ***p = 0.0003, Tg Casp3 Veh *vs* Tg Casp3 Fluox: ***p = 0.009; with Tukey’s). **C** IL-18R and GFAP immunostaining (scale: 10 µm), and plot showing % of IL-18R^+^/GFAP^+^ cells following Veh or Fluox treatment (WT Sham Veh: *n* = 6, Tg Sham Veh: *n* = 6, Tg Casp3 Veh: *n* = 6, Tg Casp3 Fluox: *n* = 5 mice; One-Way ANOVA: F_3,19_ = 8.827; *p* = 0.0007. WT Sham Veh *vs* Tg Casp3 Veh: **p = 0.001, Tg Sham Veh *vs* Tg Casp3 Veh: **p = 0.003, Tg Casp3 Veh *vs* Tg Casp3 Fluox: **p = 0.007; with Tukey’s). **D** p-NFκB and S100β immunostaining (scale: 20 µm) and plot showing % of p-NFκB^+^/S100β^+^ cells following Veh or Fluox treatment (WT Sham Veh: *n* = 6, Tg Sham Veh: *n* = 6, Tg Casp3 Veh: *n* = 6, Tg Casp3 Fluox: *n* = 5 mice; One-Way ANOVA: F_3,19_ = 33.70; *p* < 0.0001. WT Sham Veh *vs* Tg Casp3 Veh: ****p < 0.0001, Tg Sham Veh *vs* Tg Casp3 Veh: ****p < 0.0001, Tg Casp3 Veh *vs* Tg Casp3 Fluox: ****p < 0.0001; with Tukey’s). **E** Complement C3 and GFAP immunostaining (scale: 10 µm), and plot showing C3 levels following Fluox treatment (WT Sham Veh: *n* = 6, Tg Sham Veh: *n* = 6, Tg Casp3 Veh: *n* = 6, Tg Casp3 Fluox: *n* = 5 mice; One-Way ANOVA: F_3,19_ = 15.50; *p* < 0.0001. WT Sham Veh *vs* Tg Casp3 Veh: ****p < 0.0001, Tg Sham Veh *vs* Tg Casp3 Veh: ****p < 0.0001, Tg Casp3 Veh *vs* Tg Casp3 Fluox: **p = 0.002; with Tukey’s). **F** AT8 immunostaining in WT Sham Veh, Tg Sham Veh, Tg Casp3 Veh and Tg Casp Fluox hippocampi and plot showing AT8^+^ area on total hippocampal area (scale bar: 250 µm; WT Sham Veh: *n* = 6, Tg Sham Veh: *n* = 6, Tg Casp3 Veh: *n* = 7, Tg Casp Fluox: *n* = 5 mice/group. One-Way ANOVA: F_3,20_ = 6.114; *p* = 0.004. WT Sham Veh *vs* Tg Casp3 Veh: *p = 0.011, Tg Sham Veh *vs* Tg Casp3 Veh: **p = 0.008, Tg Casp3 Veh *vs* Tg Casp Fluox: *p = 0.036). The insets and right plot show AT8-reactive cells and AT8 levels (scale: 20 µm; One-Way ANOVA: F_3,20_ = 41.32; *p* < 0.0001. WT Sham Veh *vs* Tg Casp3 Veh: ****p < 0.0001, Tg Sham Veh *vs* Tg Casp3 Veh: ****p < 0.0001, Tg Casp3 Veh *vs* Tg Casp Fluox: ****p < 0.0001, with Tukey’s)

To evaluate whether restoring the monoaminergic tone could reduce the astrocyte response and tau hyperphosphorylation in the context of AD, we treated Tg Casp3 mice with L-DOPA (4-day treatment) or fluoxetine (initiated one day post-lesion). Both interventions markedly attenuated the hippocampal astrocytic response, as evidenced by a significant reduction in GFAP^+^ cell number compared to Tg Casp3 mice treated with vehicle (Fig. 8A and Fig. 9A). Concomitantly, IL-18 expression in microglia was suppressed (Fig. 8B and Fig. 9B) and this was accompanied by a reduced portion of astrocytes expressing IL-18R and p-NFκB (Fig. 8C-D and Fig. 9C-D). Astrocytic C3 immunoreactivity was also markedly decreased after treatments (Fig. 8E and Fig. 9E). Crucially, AT8 immunostaining revealed a significant reduction in hippocampal hyperphosphorylated tau in treated Tg Casp3 mice (Fig. 8F and Fig. 9F).

Collectively, these results demonstrate that both dopaminergic and serotonergic treatments effectively suppress microglial pro-inflammatory cytokine release, astrocyte reactivity, and the downstream tau hyperphosphorylation in the hippocampus of Tg Casp3 mice.

## Discussion

Here, we provide two main results: i) in C57BL/6N mice, the combined loss of DA and 5-HT in the hippocampus induces microglia reactivity characterized by NLRP3 inflammasome activation and IL-1β release, that is considerably dampened by dopaminergic or serotonergic drugs; ii) in Tg2576 mice, the combined loss of DA and 5-HT triggers a greater microglia response, together with an abnormal astrocyte reactivity. In the Tg Casp3 hippocampus, reactive astrocytes foster the appearance of tau pathology and premature Aβ plaque formation (Fig. 7K). Crucially, pro-inflammatory cytokine release by microglia, astrocyte reactivity and tau hyperphosphorylation can all be significantly attenuated by L-DOPA or fluoxetine treatments.

Importantly, monoamine‑driven neuroinflammation in Casp3 mice occurs independently of Aβ pathology. While in classic AD models (e.g., Tg2576, 3xTg‑AD, 5xFAD) hippocampal microglia reactivity is typically attributed to Aβ plaque accumulation [95–99], these AD mice also exhibit early midbrain degeneration and concomitant monoamine loss [50, 56, 60, 95, 100]. Nonetheless, the contribution of the monoamine depletion to neuroinflammation remains debatable if we consider the microglia response to Aβ [101, 102]. Here, by selectively inducing monoaminergic loss in Aβ‑naïve C57BL/6N mice, we demonstrate that combined DA/5‑HT depletion is both necessary and sufficient to trigger NLRP3‑dependent IL‑1β release. Furthermore, our data distinguish microglia reactivity from downstream astrocyte activation: the microglia response emerges as an upstream event in the neuroinflammatory cascade and correlates with the onset of neuropsychiatric symptoms [13, 103], whereas astrocyte reactivity appears more closely associated with Aβ-tau interactions [13, 104–106]. Notably, this monoamine‐driven microglia response in Casp3 mice is sex‑independent, manifesting similarly in males and females.

Selective lesioning of either DA (6OHDA mice) or 5-HT (Casp3^IPN^ mice) alone, however, is insufficient to induce microglia-mediated neuroinflammation. Nonetheless, we found that fluoxetine entirely prevented the increase in NLRP3 and IL-1β levels in the hippocampus of Casp3 mice, in line with growing evidence that SSRI treatment can dampen neuroinflammatory responses [107–109]. Similarly, both L-DOPA and the selective D1 receptor agonist A68930 attenuated the microglia-mediated pro-inflammatory phenotype in Casp3 mice, consistent with reports highlighting that DA promotes NLRP3 ubiquitination and degradation, thereby curtailing IL‑1β synthesis [19].

When dual monoamine deprivation was induced at the pre‑plaque stage in Tg2576 mice, we observed an exacerbated microglial response accompanied by aberrant astrocyte reactivity. This key finding indicates that, in the context of ongoing Aβ pathology, the astrocyte response— and the resulting tau hyperphosphorylation in the hippocampus—critically depend on the reduced monoaminergic tone. Supporting this, treatment with either L‑DOPA or fluoxetine significantly attenuated both astrocyte reactivity and hippocampal tau pathology in Tg Casp3 mice.

From a clinical perspective, these results resonate with three main observations. First, CSF and plasma GFAP levels are elevated in cognitively unimpaired individuals with positivity to brain amyloid pathology, establishing astrocyte response as an early mediator linking Aβ accumulation to the downstream tauopathy [110]. Second, reduced midbrain volumes observed on Magnetic Resonance Imaging are strongly correlated with faster progression from MCI to dementia due-to-AD, while early functional disconnection of mesocorticolimbic regions reliably predicts subsequent cognitive decline [21, 24, 27, 29, 31]. Third, DA‑based therapies, such as L‑DOPA/carbidopa, not only delay the progression from MCI to dementia, but also lower CSF levels of Aβ and tau in patients [111, 112]. These effects are consistent with preclinical evidence that DA inhibits GSK-3β [113], up-regulates neprilysin [114], and promotes amyloid fibril disaggregation [115, 116], thereby mitigating the neurotoxic environment. A similar function against AD pathology has been proposed for 5-HT and SSRIs, reducing amyloid load or tau pathology [107, 117].

Here, the notion that deficits in monoaminergic nuclei may drive AD-relevant neuroinflammation and tau pathology supports the clinical studies linking Aβ to microglia and astrocyte reactivity and tau across the AD spectrum [5–8]. Furthermore, it may answer the unresolved question of why a significant number of Aβ-positive individuals do not develop tau pathology and thus remain cognitively unimpaired [4].

Our findings suggest that dysfunction in midbrain monoaminergic nuclei—key components of the “isodendritic core”, known for their vulnerability in age‑related neurodegenerative disorders—may actively drive AD‑relevant astrocyte reactivity and downstream tau pathology [32, 118–120]. By targeting the VTA, SNpc and IPN—nuclei that collectively provide dopaminergic and serotonergic input to the hippocampus—we demonstrate that progressive midbrain degeneration triggers a cascade of neuroinflammatory, amyloidogenic and tau-related processes. These results extend the role of monoamines beyond behavioral modulation to the direct regulation of the mechanistic process determined by the glial and neuronal vulnerability. This underscores the therapeutic potential of preserving monoaminergic integrity as a strategy for early intervention in AD.

## Conclusions

Overall, our study proves the direct involvement of midbrain monoamines in controlling hippocampal glial homoeostasis. The loss of dopaminergic and serotoninergic input initiates NLRP3‑dependent microglia reactivity, downstream astroglia activation, and drives tauopathy—a cascade that can be attenuated by restoring DA or 5-HT signaling. These results provide a mechanistic insight into how early midbrain degeneration may accelerate AD pathology. Clinically, our data resonate with evidence that individuals exhibiting reduced midbrain volumes experience more rapid progression from MCI to dementia, likely reflecting the loss of monoaminergic anti‑inflammatory tone. These findings also highlight the importance of stratifying patients by midbrain integrity in clinical trials. More broadly, they support a precision‑medicine paradigm in which early monoamine‑restorative interventions could intercept neuroinflammation and glial dysregulation during the preclinical and/or prodromal AD phases, thereby slowing—or even halting—the manifestation/progression of cognitive symptoms. Such an approach holds considerable promise for restoring neuromodulatory control over vulnerable hippocampal circuits and for modifying disease trajectory.

## Supplementary Information

Supplementary Material 1.

## Data Availability

All data is available in the main text or the supplementary materials. Raw data can also be freely-available by the corresponding author upon request.

## Abbreviations

5-HT: Serotonin
6OHDA: 6-Hydroxy-Dopamine
AAV: Adeno-Associated Virus
Aβ: Amyloid-β
aCSF: Artificial Cerebro-Spinal Fluid
AD: Alzheimer’s Disease
APP: Aβ Precursor Protein
C3: Complement component 3
C3aR: Receptor for the Complement C3a
Casp3: Caspase-3
DA: Dopamine
DAT: DA transporter
DO: Displaced Objects
GSK3β: Glycogen Synthase Kinase 3β
HPLC: High Performance Liquid Chromatography
I/O: Input–Output
IL-18R: IL-18 Receptor
IL-1β: Interleukin-1β
IL-1β-Ra: IL-1β receptor antagonist
IPN: Interpeduncular Nucleus
LC: Locus Coeruleus
LTP: Long-Term Potentiation
MAP2: Microtubule-Associated Protein 2
MAPKs: Mitogen-Activated Protein Kinases
MCI: Mild Cognitive Impairment
NAc: Nucleus Accumbens
NDO: Non-Displaced Objects
NE: Norepinephrine
NET: NE Transporter
NFκB: Nuclear Factor kappa B
NLRP3: NOD-, LRR- and Pyrin domain-containing protein 3
NOR: Novel Object Recognition
PB: Phosphate buffer
PPR: Paired-Pulse Ratio
SERT: 5-HT Transporter
SNpc: Substantia Nigra *pars compacta*
SOR: Spatial Object Recognition
SSRI: 5-HT Reuptake Inhibitor
TH: Tyrosine-Hydroxylase
VTA: Ventral Tegmental Area
WT: Wild-Type

## References

[1] Jia J, Ning Y, Chen M, Wang S, Yang H, Li F, et al. Biomarker changes during 20 years preceding Alzheimer’s disease. N Engl J Med. 2024F;390(8):712–22. 38381674 10.1056/NEJMoa2310168

[2] Ossenkoppele R, Pichet Binette A, Groot C, Smith R, Strandberg O, Palmqvist S, et al. Amyloid and tau PET-positive cognitively unimpaired individuals are at high risk for future cognitive decline. Nat Med. 2022;28(11):2381–7. 36357681 10.1038/s41591-022-02049-x PMC9671808

[3] Hanseeuw BJ, Betensky RA, Jacobs HIL, Schultz AP, Sepulcre J, Becker JA, et al. Association of amyloid and tau with cognition in preclinical Alzheimer disease: a longitudinal study. JAMA Neurol. 2019A;76(8):915–24. 31157827 10.1001/jamaneurol.2019.1424 PMC6547132

[4] Josephs KA, Weigand SD, Whitwell JL. Characterizing Amyloid-Positive Individuals With Normal Tau PET Levels After 5 Years. Neurology. 2022May 31;98(22):e2282–92. 35314506 10.1212/WNL.0000000000200287 PMC9162162

[5] Ising C, Venegas C, Zhang S, Scheiblich H, Schmidt SV, Vieira-Saecker A, et al. Nlrp3 inflammasome activation drives tau pathology. Nature. 2019;575(7784):669–73. 31748742 10.1038/s41586-019-1769-z PMC7324015

[6] Pascoal TA, Benedet AL, Ashton NJ, Kang MS, Therriault J, Chamoun M, et al. Microglial activation and tau propagate jointly across Braak stages. Nat Med. 2021;27(9):1592–9. 34446931 10.1038/s41591-021-01456-w

[7] Bellaver B, Povala G, Ferreira PCL, Ferrari-Souza JP, Leffa DT, Lussier FZ, et al. Astrocyte reactivity influences amyloid-β effects on tau pathology in preclinical Alzheimer’s disease. Nat Med. 2023;29(7):1775–81. 37248300 10.1038/s41591-023-02380-x PMC10353939

[8] Appleton J, Finn Q, Zanotti-Fregonara P, Yu M, Faridar A, Nakawah MO, et al. Brain inflammation co-localizes highly with tau in mild cognitive impairment due to early-onset Alzheimer’s disease. Brain. 2025;148:119–32. 10.1093/brain/awae234 PMC11706285 39013020

[9] Dubois B, Epelbaum S, Nyasse F, Bakardjian H, Gagliardi G, Uspenskaya O, et al. Cognitive and neuroimaging features and brain β-amyloidosis in individuals at risk of Alzheimer’s disease (INSIGHT-preAD): a longitudinal observational study. The Lancet Neurology. 2018Apr 1;17(4):335–46. 29500152 10.1016/S1474-4422(18)30029-2

[10] Shi FD, Yong VW. Neuroinflammation across neurological diseases. Science. 2025;388(eadx0043):0043. 10.1126/science.adx0043 40536983

[11] Peretti DE, Boccalini C, Ribaldi F, Scheffler M, Marizzoni M, Ashton NJ, et al. Association of glial fibrillary acid protein, Alzheimer’s disease pathology and cognitive decline. Brain. 2024 Jun 28;awae211. 10.1093/brain/awae211 PMC11629700 38940331

[12] Milà-Alomà M, Ashton NJ, Shekari M, Salvadó G, Ortiz-Romero P, Montoliu-Gaya L, et al. Plasma p-tau231 and p-tau217 as state markers of amyloid-β pathology in preclinical Alzheimer’s disease. Nat Med. 2022Sep;28(9):1797–801. 35953717 10.1038/s41591-022-01925-w PMC9499867

[13] Schaffer Aguzzoli C, Ferreira PCL, Povala G, Ferrari-Souza JP, Bellaver B, Soares Katz C, et al. Neuropsychiatric symptoms and microglial activation in patients with Alzheimer disease. JAMA Netw Open. 2023N;6(11):e2345175. 38010651 10.1001/jamanetworkopen.2023.45175 PMC10682836

[14] Shao W, Zhang Szhen, Tang M, Zhang Xhua, Zhou Z, Yin Yqing, et al. Suppression of neuroinflammation by astrocytic dopamine D2 receptors via αB-crystallin. Nature. 2013;494(7435):90–4. 23242137 10.1038/nature11748

[15] Turkin A, Tuchina O, Klempin F. Microglia function on precursor cells in the adult Hippocampus and their responsiveness to serotonin signaling. Front Cell Dev Biol. 2021;9:665739. 34109176 10.3389/fcell.2021.665739 PMC8182052

[16] Yan Y, Jiang W, Liu L, Wang X, Ding C, Tian Z, et al. Dopamine controls systemic inflammation through inhibition of NLRP3 inflammasome. Cell. 2015Jan 15;160(1–2):62–73. 25594175 10.1016/j.cell.2014.11.047

[17] Zhu J, Hu Z, Han X, Wang D, Jiang Q, Ding J, et al. Dopamine D2 receptor restricts astrocytic NLRP3 inflammasome activation via enhancing the interaction of β-arrestin2 and NLRP3. Cell Death Differ. 2018;25(11):2037–49. 29786071 10.1038/s41418-018-0127-2 PMC6219479

[18] Heneka MT, Nadrigny F, Regen T, Martinez-Hernandez A, Dumitrescu-Ozimek L, Terwel D, et al. Locus ceruleus controls Alzheimer’s disease pathology by modulating microglial functions through norepinephrine. Proc Natl Acad Sci U S A. 2010Mar 30;107(13):6058–63. 20231476 10.1073/pnas.0909586107 PMC2851853

[19] Possemato E, La Barbera L, Nobili A, Krashia P, D’Amelio M. The role of dopamine in NLRP3 inflammasome inhibition: implications for neurodegenerative diseases. Ageing Res Rev. 2023;87:101907. 36893920 10.1016/j.arr.2023.101907

[20] D’Amelio M, Serra L, Bozzali M. Ventral tegmental area in prodromal Alzheimer’s disease: bridging the gap between mice and humans. J Alzheimers Dis. 2018;63(1):181–3. 29630556 10.3233/JAD-180094

[21] De Marco M, Venneri A. Volume and connectivity of the ventral tegmental area are linked to neurocognitive signatures of Alzheimer’s disease in humans. J Alzheimers Dis. 2018;63(1):167–80. 29578486 10.3233/JAD-171018

[22] Iaccarino L, Sala A, Caminiti SP, Presotto L, Perani D, Alzheimer’s Disease Neuroimaging Initiative. In vivo MRI structural and PET metabolic connectivity study of dopamine pathways in Alzheimer’s disease. J Alzheimers Dis. 2020;75(3):1003–16. 32390614 10.3233/JAD-190954

[23] Krashia P, Nobili A, D’Amelio M. Unifying Hypothesis of Dopamine Neuron Loss in Neurodegenerative Diseases: Focusing on Alzheimer’s Disease. Front Mol Neurosci. 2019May;17:12. 10.3389/fnmol.2019.00123 PMC6534044 31156387

[24] Manca R, Valera-Bermejo JM, Venneri A, Alzheimer’s Disease Neuroimaging Initiative. Accelerated atrophy in dopaminergic targets and medial temporo-parietal regions precedes the onset of delusions in patients with Alzheimer’s disease. Eur Arch Psychiatry Clin Neurosci. 2023;273(1):229–41. 35554669 10.1007/s00406-022-01417-5 PMC9958148

[25] Sala A, Caminiti SP, Presotto L, Pilotto A, Liguori C, Chiaravalloti A, et al. In vivo human molecular neuroimaging of dopaminergic vulnerability along the Alzheimer’s disease phases. Alzheimers Res Ther. 2021Nov 12;13(1):187. 34772450 10.1186/s13195-021-00925-1 PMC8588696

[26] Serra L, D’Amelio M, Di Domenico C, Dipasquale O, Marra C, Mercuri NB, et al. In vivo mapping of brainstem nuclei functional connectivity disruption in Alzheimer’s disease. Neurobiol Aging. 2018Dec;72:72–82. 30237073 10.1016/j.neurobiolaging.2018.08.012

[27] Venneri A, De Marco M. Reduced monoaminergic nuclei MRI signal detectable in pre-symptomatic older adults with future memory decline. Sci Rep. 2020Oct 30;10(1):18707. 33127923 10.1038/s41598-020-71368-1 PMC7603335

[28] Tondo G, Boccalini C, Caminiti SP, Presotto L, Filippi M, Magnani G, et al. Brain metabolism and microglia activation in mild cognitive impairment: a combined [18F]FDG and [11C]-(R)-PK11195 PET study. J Alzheimers Dis. 2021J;80(1):433–45. 33579848 10.3233/JAD-201351

[29] Dutt S, Li Y, Mather M, Nation DA, for the Alzheimer’s Disease Neuroimaging Initiative. Brainstem substructures and cognition in prodromal Alzheimer’s disease. Brain Imaging Behav. 2021;15(5):2572–82. 33646514 10.1007/s11682-021-00459-y PMC8500899

[30] De Marco M, Venneri A. Volume and connectivity of the ventral tegmental area are linked to neurocognitive signatures of Alzheimer’s disease in humans. J Alzheimers Dis. 2018;63(1):167–80. 29578486 10.3233/JAD-171018

[31] Serra L, D’Amelio M, Esposito S, Di Domenico C, Koch G, Marra C, et al. Ventral tegmental area disconnection contributes two years early to correctly classify patients converted to Alzheimer’s disease: implications for treatment. J Alzheimers Dis. 2021;82(3):985–1000. 34120905 10.3233/JAD-210171

[32] Manca R, De Marco M, Soininen H, Ruffini L, Venneri A. Changes in neurotransmitter-related functional connectivity along the Alzheimer’s disease continuum. Brain Commun. 2025;7(1):fcaf008. 39980737 10.1093/braincomms/fcaf008 PMC11840171

[33] Pilotto A, Galli A, Sala A, Caminiti SP, Presotto L, Liguori C, et al. Dopaminergic deficits along the spectrum of Alzheimer’s disease. Mol Psychiatry. 2025;30:3069–76. 10.1038/s41380-025-02913-5 39890920

[34] Zhang Y, Liang Y, Gu Y. The dopaminergic system and Alzheimer’s disease. Neural Regen Res. 2025Sep 1;20(9):2495–512. 39314145 10.4103/NRR.NRR-D-24-00230 PMC11801300

[35] Krashia P, Spoleti E, D’Amelio M. The VTA dopaminergic system as diagnostic and therapeutical target for Alzheimer’s disease. Front Psychiatry. 2022;13:1039725. 36325523 10.3389/fpsyt.2022.1039725 PMC9618946

[36] McNamara CG, Tejero-Cantero Á, Trouche S, Campo-Urriza N, Dupret D. Dopaminergic neurons promote hippocampal reactivation and spatial memory persistence. Nat Neurosci. 2014Dec;17(12):1658–60. 25326690 10.1038/nn.3843 PMC4241115

[37] Rossato JI, Bevilaqua LRM, Izquierdo I, Medina JH, Cammarota M. Dopamine controls persistence of long-term memory storage. Science. 2009Aug 21;325(5943):1017–20. 19696353 10.1126/science.1172545

[38] Tsetsenis T, Badyna JK, Wilson JA, Zhang X, Krizman EN, Subramaniyan M, et al. Midbrain dopaminergic innervation of the hippocampus is sufficient to modulate formation of aversive memories. Proc Natl Acad Sci U S A. 2021Oct 5;118(40):e2111069118. 34580198 10.1073/pnas.2111069118 PMC8501778

[39] Groenewegen HJ, Steinbusch HW. Serotonergic and non-serotonergic projections from the interpeduncular nucleus to the ventral hippocampus in the rat. Neurosci Lett. 1984Sep 28;51(1):19–24. 6096770 10.1016/0304-3940(84)90256-8

[40] Khatami L, Khodagholi F, Motamedi F. Reversible inactivation of interpeduncular nucleus impairs memory consolidation and retrieval but not learning in rats: A behavioral and molecular study. Behav Brain Res. 2018Apr;16(342):79–88. 10.1016/j.bbr.2018.01.012 29355671

[41] Hsiao K, Chapman P, Nilsen S, Eckman C, Harigaya Y, Younkin S, et al. Correlative memory deficits, Abeta elevation, and amyloid plaques in transgenic mice. Science. 1996Oct 4;274(5284):99–102. 8810256 10.1126/science.274.5284.99

[42] Yang CF, Chiang MC, Gray DC, Prabhakaran M, Alvarado M, Juntti SA, et al. Sexually dimorphic neurons in the ventromedial hypothalamus govern mating in both sexes and aggression in males. Cell. 2013M;153(4):896–909. 23663785 10.1016/j.cell.2013.04.017 PMC3767768

[43] Paxinos G, Franklin KBJ. Paxinos and Franklin’s the Mouse Brain in Stereotaxic Coordinates. Academic Press; 2019. 357 p.

[44] Cordella A, Krashia P, Nobili A, Pignataro A, La Barbera L, Viscomi MT, et al. Dopamine loss alters the hippocampus-nucleus accumbens synaptic transmission in the Tg2576 mouse model of Alzheimer’s disease. Neurobiol Dis. 2018;116:142–54. 29778899 10.1016/j.nbd.2018.05.006

[45] Spoleti E, Krashia P, La Barbera L, Nobili A, Lupascu CA, Giacalone E, et al. Early derailment of firing properties in CA1 pyramidal cells of the ventral hippocampus in an Alzheimer’s disease mouse model. Exp Neurol. 2021Dec;31(350):113969. 10.1016/j.expneurol.2021.113969 34973962

[46] De Paolis ML, Loffredo G, Krashia P, La Barbera L, Nobili A, Cauzzi E, et al. Repetitive prefrontal tDCS activates VTA dopaminergic neurons, resulting in attenuation of Alzheimer’s Disease-like deficits in Tg2576 mice. Alzheimers Res Ther. 2025Apr 29;17(1):94. 40301905 10.1186/s13195-025-01736-4 PMC12039073

[47] Holmes BB, Furman JL, Mahan TE, Yamasaki TR, Mirbaha H, Eades WC, et al. Proteopathic tau seeding predicts tauopathy in vivo. Proc Natl Acad Sci. 2014Oct 14;111(41):E4376–85. 25261551 10.1073/pnas.1411649111 PMC4205609

[48] Krashia P, Cordella A, Nobili A, La Barbera L, Federici M, Leuti A, et al. Blunting neuroinflammation with resolvin D1 prevents early pathology in a rat model of Parkinson’s disease. Nat Commun. 2019Sep 2;10(1):3945. 31477726 10.1038/s41467-019-11928-w PMC6718379

[49] Tortolani D, Decandia D, Giacovazzo G, Scipioni L, Panuccio A, Ciaramellano F, et al. Chronic palmitoylethanolamide administration via slow-release subcutaneous pellets promotes neuroprotection and mitigates neuroinflammation in the Tg2576 mouse model of Alzheimer’s disease. Front Cell Neurosci. 2025;19:1571428. 40313591 10.3389/fncel.2025.1571428 PMC12043567

[50] La Barbera L, Nobili A, Cauzzi E, Paoletti I, Federici M, Saba L, et al. Upregulation of Ca2+-binding proteins contributes to VTA dopamine neuron survival in the early phases of Alzheimer’s disease in Tg2576 mice. Mol Neurodegener. 2022Nov 25;17(1):76. 36434727 10.1186/s13024-022-00580-6 PMC9700939

[51] Martin M. Cutadapt removes adapter sequences from high-throughput sequencing reads. EMBnet.journal. 2011;17(1):10–2.

[52] Dobin A, Davis CA, Schlesinger F, Drenkow J, Zaleski C, Jha S, et al. STAR: ultrafast universal RNA-seq aligner. Bioinformatics. 2013;29(1):15–21. 23104886 10.1093/bioinformatics/bts635 PMC3530905

[53] Pertea M, Pertea GM, Antonescu CM, Chang TC, Mendell JT, Salzberg SL. Stringtie enables improved reconstruction of a transcriptome from RNA-seq reads. Nat Biotechnol. 2015;33(3):290–5. 25690850 10.1038/nbt.3122 PMC4643835

[54] Love MI, Huber W, Anders S. Moderated estimation of fold change and dispersion for RNA-seq data with DESeq2. Genome Biol. 2014;15(12):550. 25516281 10.1186/s13059-014-0550-8 PMC4302049

[55] Romoli M, Krashia P, Sen A, Franciotta D, Gastaldi M, Nobili A, et al. Hippocampal epileptogenesis in autoimmune encephalitis. Ann Clin Transl Neurol. 2019Oct 15;6(11):2261–9. 31617317 10.1002/acn3.50919 PMC6856617

[56] Nobili A, Latagliata EC, Viscomi MT, Cavallucci V, Cutuli D, Giacovazzo G, et al. Dopamine neuronal loss contributes to memory and reward dysfunction in a model of Alzheimer’s disease. Nat Commun. 2017;8:14727. 28367951 10.1038/ncomms14727 PMC5382255

[57] Nobili A, Krashia P, Cordella A, La Barbera L, Dell’Acqua MC, Caruso A, et al. Ambra1 shapes hippocampal inhibition/excitation balance: role in neurodevelopmental disorders. Mol Neurobiol. 2018;55(10):7921–40. 29488136 10.1007/s12035-018-0911-5 PMC6132777

[58] D’Amelio M, Cavallucci V, Middei S, Marchetti C, Pacioni S, Ferri A, et al. Caspase-3 triggers early synaptic dysfunction in a mouse model of Alzheimer’s disease. Nat Neurosci. 2011Jan;14(1):69–76. 21151119 10.1038/nn.2709

[59] Wang F, Tian ZC, Ding H, Yang XJ, Wang FD, Ji RX, et al. A sensory-motor-sensory circuit underlies antinociception ignited by primary motor cortex in mice. Neuron. 2025Jun 18;113(12):1947-1968.e7. 40239652 10.1016/j.neuron.2025.03.027

[60] La Barbera L, Vedele F, Nobili A, Krashia P, Spoleti E, Latagliata EC, et al. Nilotinib restores memory function by preventing dopaminergic neuron degeneration in a mouse model of Alzheimer’s Disease. Prog Neurobiol. 2021Jul;1(202):102031. 10.1016/j.pneurobio.2021.102031 33684513

[61] Cabib S, Pascucci T, Ventura R, Romano V, Puglisi-Allegra S. The behavioral profile of severe mental retardation in a genetic mouse model of phenylketonuria. Behav Genet. 2003M;33(3):301–10. 12837019 10.1023/a:1023498508987

[62] Nardecchia F, Orlando R, Iacovelli L, Colamartino M, Fiori E, Leuzzi V, et al. Targeting mGlu5 metabotropic glutamate receptors in the treatment of cognitive dysfunction in a mouse model of phenylketonuria. Front Neurosci. 2018;12:154. 29615849 10.3389/fnins.2018.00154 PMC5864888

[63] Sciamanna G, Ponterio G, Vanni V, Laricchiuta D, Martella G, Bonsi P, et al. Optogenetic Activation of Striatopallidal Neurons Reveals Altered HCN Gating in DYT1 Dystonia. Cell Rep. 2020May 19;31(7):107644. 32433955 10.1016/j.celrep.2020.107644

[64] Anders S, Huber W. Differential expression analysis for sequence count data. Genome Biol. 2010;11(10):R106. 20979621 10.1186/gb-2010-11-10-r106 PMC3218662

[65] Montone KT, Fass B, Hamill GS. Serotonergic and nonserotonergic projections from the rat interpeduncular nucleus to the septum, hippocampal formation and raphe: a combined immunocytochemical and fluorescent retrograde labelling study of neurons in the apical subnucleus. Brain Res Bull. 1988;20(2):233–40. 2836039 10.1016/0361-9230(88)90183-9

[66] Wirtshafter D, Asin KE, Lorens SA. Serotonin-immunoreactive projections to the hippocampus from the interpeduncular nucleus in the rat. Neurosci Lett. 1986Mar 14;64(3):259–62. 2421209 10.1016/0304-3940(86)90338-1

[67] Gasbarri A, Verney C, Innocenzi R, Campana E, Pacitti C. Mesolimbic dopaminergic neurons innervating the hippocampal formation in the rat: a combined retrograde tracing and immunohistochemical study. Brain Res. 1994Dec 30;668(1–2):71–9. 7704620 10.1016/0006-8993(94)90512-6

[68] Kempadoo KA, Mosharov EV, Choi SJ, Sulzer D, Kandel ER. Dopamine release from the locus coeruleus to the dorsal hippocampus promotes spatial learning and memory. Proc Natl Acad Sci U S A. 2016;113(51):14835–40. 27930324 10.1073/pnas.1616515114 PMC5187750

[69] Lammel S, Steinberg EE, Földy C, Wall NR, Beier K, Luo L, et al. Diversity of transgenic mouse models for selective targeting of midbrain dopamine neurons. Neuron. 2015Jan 21;85(2):429–38. 25611513 10.1016/j.neuron.2014.12.036 PMC5037114

[70] Lindeberg J, Usoskin D, Bengtsson H, Gustafsson A, Kylberg A, Söderström S, et al. Transgenic expression of Cre recombinase from the tyrosine hydroxylase locus. Genesis. 2004;40(2):67–73. 15452869 10.1002/gene.20065

[71] D’Amelio M, Cavallucci V, Cecconi F. Neuronal caspase-3 signaling: not only cell death. Cell Death Differ. 2010;17(7):1104–14. 19960023 10.1038/cdd.2009.180

[72] Gompf HS, Budygin EA, Fuller PM, Bass CE. Targeted genetic manipulations of neuronal subtypes using promoter-specific combinatorial AAVs in wild-type animals. Front Behav Neurosci. 2015;9:152. 10.3389/fnbeh.2015.00152 PMC4488755 26190981

[73] Broussard JI, Yang K, Levine AT, Tsetsenis T, Jenson D, Cao F, et al. Dopamine regulates aversive contextual learning and associated in vivo synaptic plasticity in the hippocampus. Cell Rep. 2016;14(8):1930–9. 26904943 10.1016/j.celrep.2016.01.070 PMC4772154

[74] Teixeira CM, Rosen ZB, Suri D, Sun Q, Hersh M, Sargin D, et al. Hippocampal 5-HT Input Regulates Memory Formation and Schaffer Collateral Excitation. Neuron. 2018Jun 6;98(5):992-1004.e4. 29754752 10.1016/j.neuron.2018.04.030 PMC6383566

[75] Heneka MT, Kummer MP, Stutz A, Delekate A, Schwartz S, Vieira-Saecker A, et al. NLRP3 is activated in Alzheimer’s disease and contributes to pathology in APP/PS1 mice. Nature. 2013Jan 31;493(7434):674–8. 23254930 10.1038/nature11729 PMC3812809

[76] Venegas C, Heneka MT. Inflammasome-mediated innate immunity in Alzheimer’s disease. FASEB J. 2019Dec;33(12):13075–84. 31702392 10.1096/fj.201900439

[77] Nisticò R, Mango D, Mandolesi G, Piccinin S, Berretta N, Pignatelli M, et al. Inflammation subverts hippocampal synaptic plasticity in experimental multiple sclerosis. PLoS ONE. 2013J;8(1):e54666. 23355887 10.1371/journal.pone.0054666 PMC3552964

[78] Lima LB, Bueno D, Leite F, Souza S, Gonçalves L, Furigo IC, et al. Afferent and efferent connections of the interpeduncular nucleus with special reference to circuits involving the habenula and raphe nuclei. J Comp Neurol. 2017;525(10):2411–42. 28340505 10.1002/cne.24217

[79] Chae S, Lee Hju, Lee HE, Kim J, Jeong YJ, Lin Y, et al. The dopamine analogue CA140 alleviates AD pathology, neuroinflammation, and rescues synaptic/cognitive functions by modulating DRD1 signaling or directly binding to Abeta. J Neuroinflammation. 2024;21(1):200. 39129007 10.1186/s12974-024-03180-x PMC11317008

[80] Pike AF, Longhena F, Faustini G, van Eik JM, Gombert I, Herrebout MAC, et al. Dopamine signaling modulates microglial NLRP3 inflammasome activation: implications for Parkinson’s disease. J Neuroinflammation. 2022;19:50. 10.1186/s12974-022-02410-4 PMC8848816 35172843

[81] Spoleti E, La Barbera L, Cauzzi E, De Paolis ML, Saba L, Marino R, et al. Dopamine neuron degeneration in the ventral tegmental area causes hippocampal hyperexcitability in experimental Alzheimer’s disease. Mol Psychiatry. 2024;29:1265–80. 38228889 10.1038/s41380-024-02408-9 PMC11189820

[82] Lian H, Yang L, Cole A, Sun L, Chiang ACA, Fowler SW, et al. NFκB-activated astroglial release of complement C3 compromises neuronal morphology and function associated with Alzheimer’s disease. Neuron. 2015Jan 7;85(1):101–15. 25533482 10.1016/j.neuron.2014.11.018 PMC4289109

[83] Lian H, Litvinchuk A, Chiang ACA, Aithmitti N, Jankowsky JL, Zheng H. Astrocyte-Microglia Cross Talk through Complement Activation Modulates Amyloid Pathology in Mouse Models of Alzheimer’s Disease. J Neurosci. 2016Jan 13;36(2):577–89. 26758846 10.1523/JNEUROSCI.2117-15.2016 PMC4710776

[84] Litvinchuk A, Wan YW, Swartzlander DB, Chen F, Cole A, Propson NE, et al. Complement C3aR inactivation attenuates tau pathology and reverses an immune network deregulated in tauopathy models and Alzheimer’s disease. Neuron. 2018Dec 19;100(6):1337-1353.e5. 30415998 10.1016/j.neuron.2018.10.031 PMC6309202

[85] Liddelow SA, Guttenplan KA, Clarke LE, Bennett FC, Bohlen CJ, Schirmer L, et al. Neurotoxic reactive astrocytes are induced by activated microglia. Nature. 2017;541(7638):481–7. 28099414 10.1038/nature21029 PMC5404890

[86] Wu T, Dejanovic B, Gandham VD, Gogineni A, Edmonds R, Schauer S, et al. Complement C3 Is Activated in Human AD Brain and Is Required for Neurodegeneration in Mouse Models of Amyloidosis and Tauopathy. Cell Rep. 2019Aug 20;28(8):2111-2123.e6. 31433986 10.1016/j.celrep.2019.07.060

[87] Xu X, Zhang A, Zhu Y, He W, Di W, Fang Y, et al. MFG-e8 reverses microglial-induced neurotoxic astrocyte (A1) via NF-κB and PI3K-Akt pathways. J Cell Physiol. 2019;234(1):904–14. 10.1002/jcp.26918 30076715

[88] Chi X, Yin S, Sun Y, Kou L, Zou W, Wang Y, et al. Astrocyte-neuron communication through the complement C3–C3aR pathway in Parkinson’s disease. Brain Behav Immun. 2025Jan;1(123):229–43. 10.1016/j.bbi.2024.09.022 39288893

[89] Ito S, Hashimoto H, Yamakawa H, Kusumoto D, Akiba Y, Nakamura T, et al. The complement C3-complement factor D-C3a receptor signalling axis regulates cardiac remodelling in right ventricular failure. Nat Commun. 2022Sep 15;13(1):5409. 36109509 10.1038/s41467-022-33152-9 PMC9478115

[90] Lovestone S, Reynolds CH, Latimer D, Davis DR, Anderton BH, Gallo JM, et al. Alzheimer’s disease-like phosphorylation of the microtubule-associated protein tau by glycogen synthase kinase-3 in transfected mammalian cells. Curr Biol. 1994Dec 1;4(12):1077–86. 7704571 10.1016/s0960-9822(00)00246-3

[91] Rankin CA, Sun Q, Gamblin TC. Tau phosphorylation by GSK-3β promotes tangle-like filament morphology. Mol Neurodegener. 2007Jun 28;2(1):12. 17598919 10.1186/1750-1326-2-12 PMC1936422

[92] Sánchez C, Pérez M, Avila J. GSK3β-mediated phosphorylation of the microtubule-associated protein 2C (MAP2C) prevents microtubule bundling. Eur J Cell Biol. 2000;79(4):252–60 Apr 1. 10826493 10.1078/s0171-9335(04)70028-x

[93] Ly PTT, Wu Y, Zou H, Wang R, Zhou W, Kinoshita A, et al. Inhibition of GSK3β-mediated BACE1 expression reduces Alzheimer-associated phenotypes. J Clin Invest. 2013Jan 2;123(1):224–35. 23202730 10.1172/JCI64516 PMC3533290

[94] Aplin AE, Gibb GM, Jacobsen JS, Gallo JM, Anderton BH. In vitro phosphorylation of the cytoplasmic domain of the amyloid precursor protein by glycogen synthase kinase-3beta. J Neurochem. 1996;67(2):699–707. 8764598 10.1046/j.1471-4159.1996.67020699.x

[95] Gloria Y, Ceyzériat K, Tsartsalis S, Millet P, Tournier BB. Dopaminergic dysfunction in the 3xTg-AD mice model of Alzheimer’s disease. Sci Rep. 2021Sep 30;11(1):19412. 34593951 10.1038/s41598-021-99025-1 PMC8484608

[96] Apelt J, Schliebs R. Beta-amyloid-induced glial expression of both pro- and anti-inflammatory cytokines in cerebral cortex of aged transgenic Tg2576 mice with Alzheimer plaque pathology. Brain Res. 2001;894(1):21–30. 11245811 10.1016/s0006-8993(00)03176-0

[97] Delizannis AT, Nonneman A, Tsering W, De Bondt A, Van den Wyngaert I, Zhang B, et al. Effects of microglial depletion and TREM2 deficiency on Aβ plaque burden and neuritic plaque tau pathology in 5XFAD mice. Acta Neuropathol Commun. 2021Sep 9;9(1):150. 34503586 10.1186/s40478-021-01251-1 PMC8428059

[98] Frautschy SA, Yang F, Irrizarry M, Hyman B, Saido TC, Hsiao K, et al. Microglial response to amyloid plaques in APPsw transgenic mice. Am J Pathol. 1998Jan;152(1):307–17. 9422548 PMC1858113

[99] Rodríguez JJ, Witton J, Olabarria M, Noristani HN, Verkhratsky A. Increase in the density of resting microglia precedes neuritic plaque formation and microglial activation in a transgenic model of Alzheimer’s disease. Cell Death Dis. 2010;1(1):e1. 21364611 10.1038/cddis.2009.2 PMC3032511

[100] Vorobyov V, Bakharev B, Medvinskaya N, Nesterova I, Samokhin A, Deev A, et al. Loss of midbrain dopamine neurons and altered apomorphine EEG effects in the 5xFAD mouse model of Alzheimer’s disease. J Alzheimers Dis. 2019;70(1):241–56. 31177214 10.3233/JAD-181246

[101] Diaz A, Limon D, Chávez R, Zenteno E, Guevara J. Aβ25-35 injection into the temporal cortex induces chronic inflammation that contributes to neurodegeneration and spatial memory impairment in rats. J Alzheimers Dis. 2012;30(3):505–22. 22430532 10.3233/JAD-2012-111979

[102] Ramírez E, Sánchez-Maldonado C, Mayoral MA, Mendieta L, Alatriste V, Patricio-Martínez A, et al. Neuroinflammation induced by the peptide amyloid-β (25–35) increase the presence of galectin-3 in astrocytes and microglia and impairs spatial memory. Neuropeptides. 2019;74:11–23. 30795916 10.1016/j.npep.2019.02.001

[103] Zhu H, Guan A, Liu J, Peng L, Zhang Z, Wang S. Noteworthy perspectives on microglia in neuropsychiatric disorders. J Neuroinflammation. 2023Oct 4;20(1):223. 37794488 10.1186/s12974-023-02901-y PMC10548593

[104] Leng F, Edison P. Neuroinflammation and microglial activation in Alzheimer disease: where do we go from here? Nat Rev Neurol. 2021Mar;17(3):157–72. 33318676 10.1038/s41582-020-00435-y

[105] Malpetti M, Kievit RA, Passamonti L, Jones PS, Tsvetanov KA, Rittman T, et al. Microglial activation and tau burden predict cognitive decline in Alzheimer’s disease. Brain. 2020May 1;143(5):1588–602. 32380523 10.1093/brain/awaa088 PMC7241955

[106] Madsen LS, Ismail R, Parbo P, Kjeldsen PL, Schaldemose JL, Hansen KV, et al. Microglial responses partially mediate the effect of Aβ on cognition in Alzheimer’s disease. Alzheimers Dement. 2024Nov;20(11):8028–37. 39392185 10.1002/alz.14298 PMC11567839

[107] Cirrito JR, Disabato BM, Restivo JL, Verges DK, Goebel WD, Sathyan A, et al. Serotonin signaling is associated with lower amyloid-β levels and plaques in transgenic mice and humans. Proc Natl Acad Sci U S A. 2011Sep 6;108(36):14968–73. 21873225 10.1073/pnas.1107411108 PMC3169155

[108] Du RH, Tan J, Sun XY, Lu M, Ding JH, Hu G. Fluoxetine inhibits NLRP3 inflammasome activation: implication in depression. Int J Neuropsychopharmacol. 2016;19(9):pyw037. 27207922 10.1093/ijnp/pyw037 PMC5043644

[109] Zabot GC, Medeiros EB, Marques AO, Macarini BMN, Vargas A, Colombo ACN, et al. The involvement of inflammation in the effect of the administration of donepezil, galantamine, memantine and/or fluoxetine in an animal model of depression and dementia. Alzheimer’s & Dementia. 2021;17(S3):e056472.

[110] Sánchez-Juan P, Valeriano-Lorenzo E, Ruiz-González A, Pastor AB, Rodrigo Lara H, López-González F, et al. Serum GFAP levels correlate with astrocyte reactivity, post-mortem brain atrophy and neurofibrillary tangles. Brain. 2024May 1;147(5):1667–79. 38634687 10.1093/brain/awae035 PMC11068326

[111] Sárkány Z, Damásio J, Macedo-Ribeiro S, Martins PM. Association between the use of levodopa/carbidopa, Alzheimer’s disease biomarkers, and cognitive decline among participants in the National Alzheimer’s Coordinating Center Uniform Data Set. Alzheimers Dement. 2025May;21(5):e70213. 40356023 10.1002/alz.70213 PMC12069009

[112] Nobili A, D’Amelio M. Reconsidering dopaminergic modulation in Alzheimer’s disease: A case for levodopa/carbidopa as a disease-modifying agent. Alzheimers Dement. 2025Jul;21(7):e70532. 40696850 10.1002/alz.70532 PMC12284314

[113] Svenningsson P, Tzavara ET, Carruthers R, Rachleff I, Wattler S, Nehls M, et al. Diverse psychotomimetics act through a common signaling pathway. Science. 2003Nov 21;302(5649):1412–5. 14631045 10.1126/science.1089681

[114] Watamura N, Kakiya N, Fujioka R, Kamano N, Takahashi M, Nilsson P, et al. The dopaminergic system promotes neprilysin-mediated degradation of amyloid-β in the brain. Sci Signal. 2024;17(848):eadk1822. 39106321 10.1126/scisignal.adk1822

[115] Cataldi R, Chia S, Pisani K, Ruggeri FS, Xu CK, Šneideris T, et al. A dopamine metabolite stabilizes neurotoxic amyloid-β oligomers. Commun Biol. 2021Jan 4;4(1):1–10. 33398040 10.1038/s42003-020-01490-3 PMC7782527

[116] Li J, Zhu M, Manning-Bog AB, Di Monte DA, Fink AL. Dopamine and L-dopa disaggregate amyloid fibrils: implications for Parkinson’s and Alzheimer’s disease. FASEB J. 2004;18(9):962–4. 15059976 10.1096/fj.03-0770fje

[117] Terstege DJ, Jabeen S, Initiative ADN, Galea LAM, Epp JR, Sargin D. Ssris reduce plasma tau and restore dorsal raphe metabolism in Alzheimer’s disease. Alzheimers Dement. 2025;21(2):e14579. 39935329 10.1002/alz.14579 PMC11814539

[118] Wearn A, Tremblay SA, Tardif CL, Leppert IR, Gauthier CJ, Baracchini G, et al. Neuromodulatory subcortical nucleus integrity is associated with white matter microstructure, tauopathy and APOE status. Nat Commun. 2024Jun 3;15(1):4706. 38830849 10.1038/s41467-024-48490-z PMC11148077

[119] Theofilas P, Dunlop S, Heinsen H, Grinberg LT. Turning on the Light Within: Subcortical Nuclei of the Isodentritic Core and their Role in Alzheimer’s Disease Pathogenesis. Journal of Alzheimer’s Disease. 2015Jan 1;46(1):17–34. 25720408 10.3233/JAD-142682 PMC4550582

[120] Ehrenberg AJ, Kelberman MA, Liu KY, Dahl MJ, Weinshenker D, Falgàs N, et al. Priorities for research on neuromodulatory subcortical systems in Alzheimer’s disease: position paper from the NSS PIA of ISTAART. Alzheimers Dement. 2023;19(5):2182–96. 36642985 10.1002/alz.12937 PMC10182252

