# Supplementary material for: Midbrain degeneration triggers astrocyte reactivity and tau pathology in experimental Alzheimer’s Disease
Source: Mol Neurodegener. 2025 Oct 13;20:105. doi: 10.1186/s13024-025-00893-2 (PMC12516900; doi:10.1186/s13024-025-00893-2)
Supplement: Supplementary file 1 — Supplementary Material 1. [file 13024_2025_893_MOESM1_ESM.docx]

**Supplemental Material**

**Content:**

**Supplemental Figures 1-12**

**Supplemental Tables 1-3**


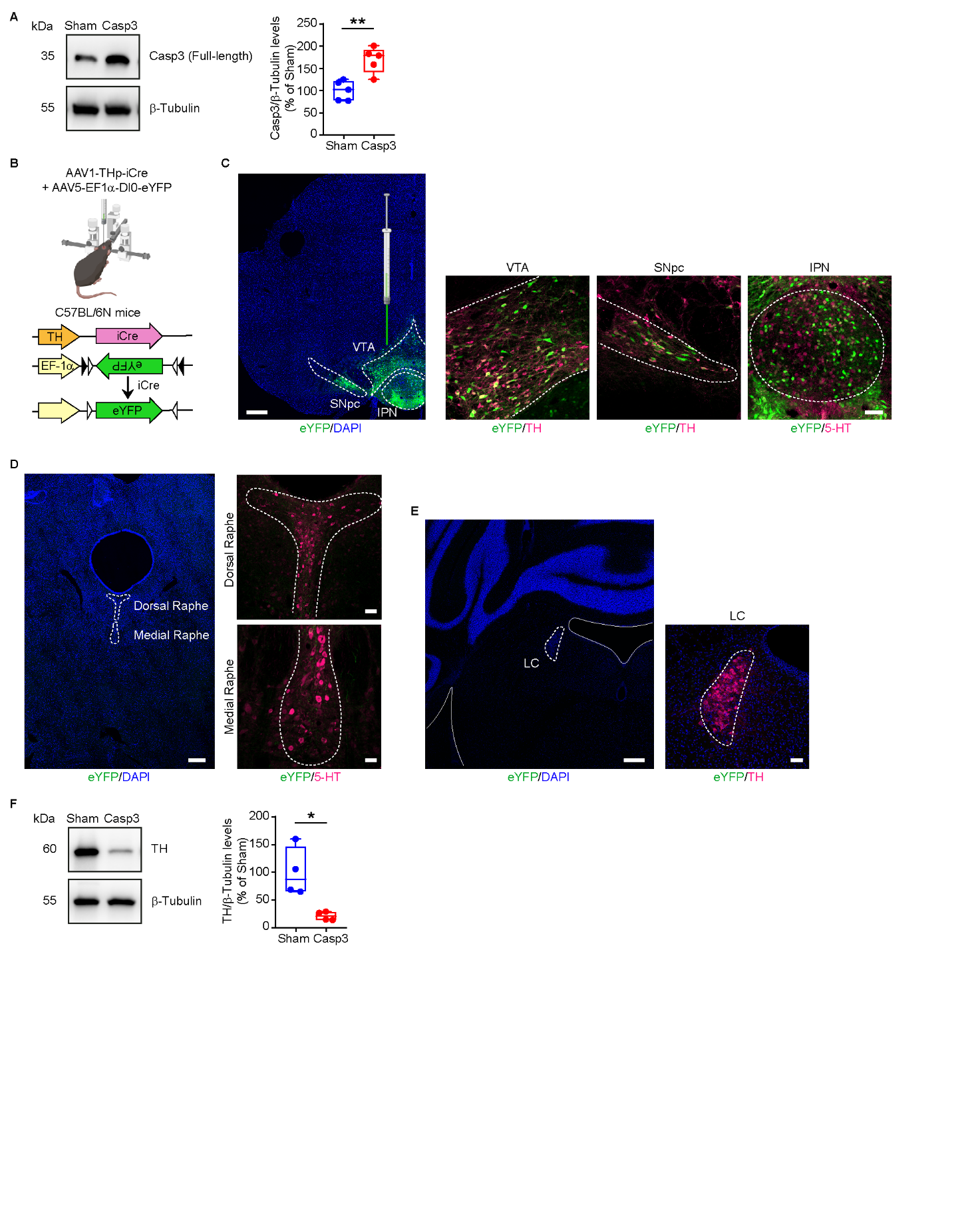
Supplemental Figure 1: Additional analysis in the midbrain of Sham and Casp3 mice, and analysis of ectopic expression of the TH promoter.

**A)** Representative western blots from the left midbrain of Sham and Casp3 mice, and plot showing the levels (expressed as % of Sham) of full-length Casp3 analysed 72 h following lesion (n = 5 mice / group; Unpaired *t*-test **p = 0.0028). β-Tubulin was used as loading control. **B)** C57BL/6N mice were unilaterally infused in the midbrain with a dual-AAV approach. The Cre-dependent reporter virus AAV5-EF1α-DIO-eYFP, driven by the EF-1α promoter, comprises the inverted eYFP reporter gene. Expression of iCre-recombinase under the TH promoter (AAV1-THp-iCre) determines recombination resulting in the expression of the eYFP reporter fluorophore. **C)** Representative images of eYFP expression in the midbrain (*left*; scale: 250 µm), and higher magnification images showing co-labelling of eYFP with TH^+^ neurons in the VTA and SNpc and with 5-HT^+^ neurons in the IPN (scale: 50 μm). **D)** Confocal images of dRaphe and mRaphe (scale: 250 µm; nuclei were counterstained with DAPI) and higher magnification images of 5-HT^+^ neurons (scale: 25 μm) from mice infused in the midbrain with the AAV5-EF1α-DIO-eYFP + AAV1-THp-iCre mix. **E)** Same as in D, but showing negative eYFP staining in TH^+^ neurons of the LC (scale: *left*, 250 µm; *right*, 50 μm). **F)** Representative western blots from the midbrain of Sham and Casp3 mice, and plot showing the levels (expressed as % of Sham) of total TH protein analysed 1-month following lesion (n = 4 mice / group; Welch’s t-test: *p = 0.035). β-Tubulin was used as loading control.


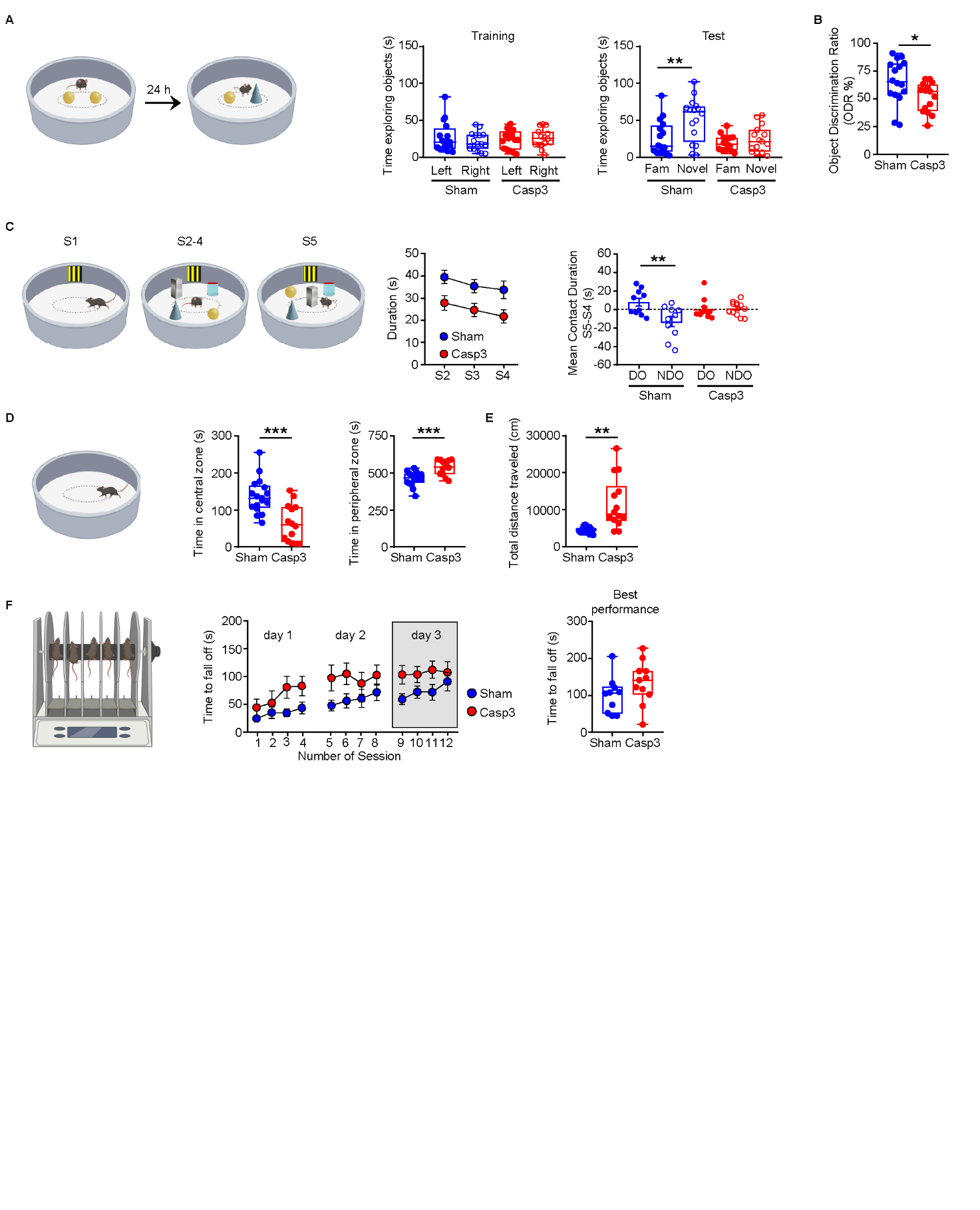


Supplemental Figure 2: Behavioral evaluation in Sham and Casp3 mice.

**A)** The plots show the exploration time spent by Sham and Casp3 mice with the novel and/or familiar object during the NOR training (*left*) and test session 24 h after training (*right*; Sham: n = 16; Casp3: n = 15 mice. Two-way RM-ANOVA: *Training:* interaction F_1,58_ = 1.064, p = 0.3065; object category F_1,58_ = 0.2731, p = 0.6032; lesion F_1,58_ = 0.01358, p = 0.9076; *Test:* interaction F_1,58_ = 3.331, p = 0.0732; object category F_1,58_ = 7.526, p = 0.0081; lesion F_1,58_ = 9.465, p = 0.0032; Sham Familiar Object *vs* Sham Novel Object: **p = 0.0092, with Tukey’s multiple comparisons test), and (**B**) the Object Discrimination Ratio (Unpaired *t*-test: *p = 0.0272). **C)** The plots show (*left*) the mean duration of exploration (± s.e.m) across all objects during sessions 2, 3, and 4 (Sham: n = 10; Casp3: n = 11 mice. Two-way RM-ANOVA: interaction F_2,57_ = 0.0181, p = 0.982; sessions F_2,57_ = 1.710, p = 0.1899; lesion F_1,57_ = 19.34, p < 0.0001) and (*right*) the mean time (± s.e.m) spent exploring Displaced (DO) or Non-Displaced Objects (NDO) during S5 minus the time spent exploring the same object category during the final familiarization session (S4), in Sham and Casp3 mice (Two-way RM-ANOVA: interaction F_1,38_ = 7.242, p = 0.0105; object category F_1,38_ = 6.904, p = 0.0123; lesion F_1,38_ = 0.6922, p = 0.4106; Sham DO *vs* Sham NDO: **p = 0.0039, with Tukey’s multiple comparisons test). **D)** The plots show the time spent in the central (*left*) and peripheral (*right*) zones of the open field arena (Sham: n = 17; Casp3: n = 14 mice. *Time in Center*: Unpaired *t*-test: ***p = 0.0002; *Time in Periphery*: Unpaired *t*-test: ***p = 0.0002), and **(E)** the locomotor activity in the whole arena of Sham and Casp3 mice during the open field test (*Total distance traveled*: Welch’s *t*-test: **p = 0.0020). **F)** The plots show the motor coordination (*left*) in Sham and Casp3 mice during the rotarod test, assessed as mean latency to fall (± s.e.m) over 4 sessions/day across 3 consecutive days (Sham: n = 10; Casp3: n = 11 mice. Two-way RM-ANOVA: interaction F_11,2092_ = 0.9112, p = 0.5304; sessions F_11,209_ = 9.573, p < 0.0001; lesion F_1,19_ = 3.314, p = 0.0845), and their best performance (*right*) on the final test day (Day 3; Unpaired *t*-test: p = 0.1562).

[Figures created using BioRender.com].


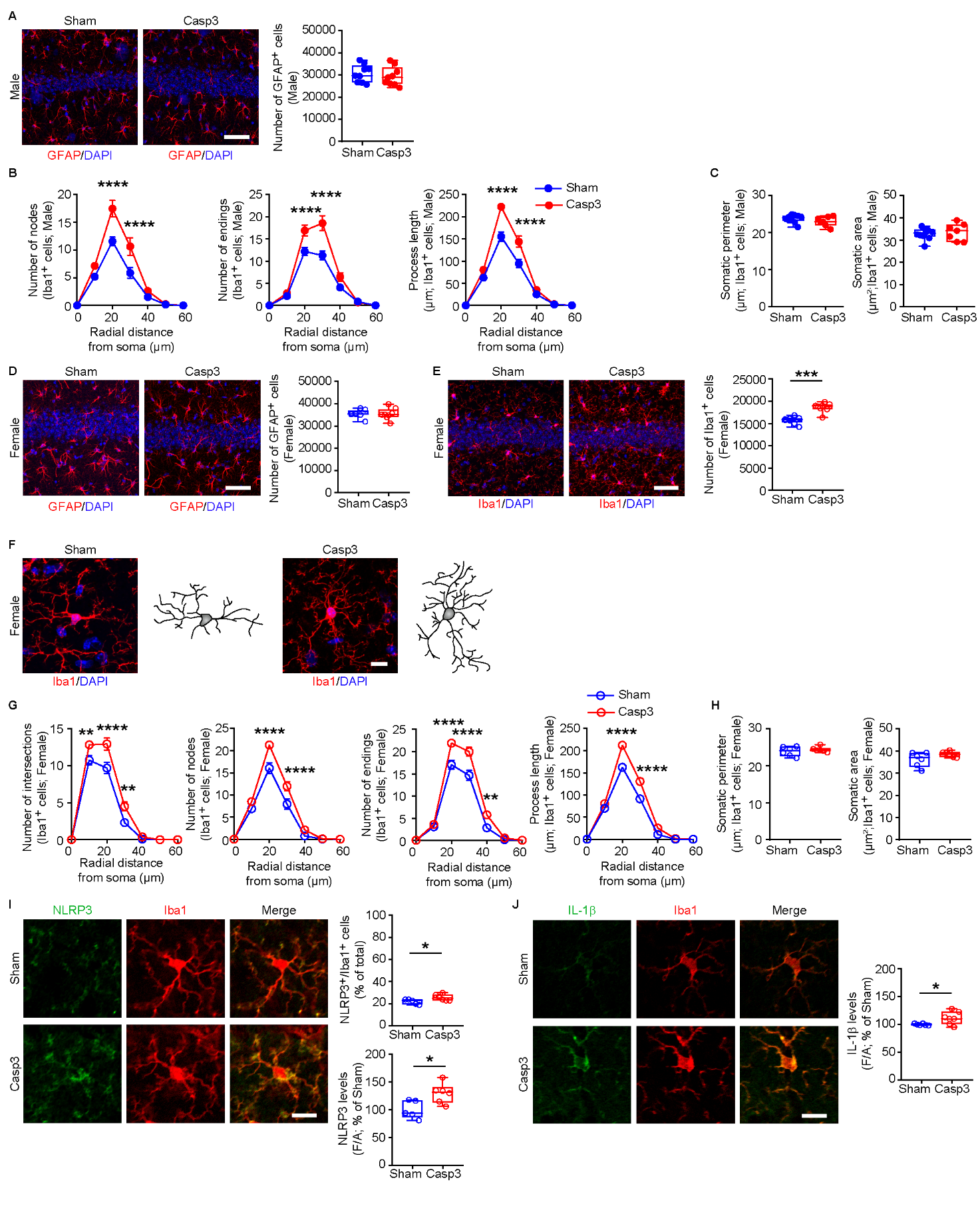


**Supplemental Figure 3: Hippocampal neuroinflammation in male and female Sham and Casp3 mice.**

**A)** Representative confocal images and plot showing stereological cell count of GFAP^+^ cells in the hippocampus of male Sham and Casp3 mice (n = 9 mice / group). Nuclei are counterstained with DAPI (scale: 50 µm). **B-C)** The graphs show parameter of Sholl analysis of microglia cells in male mice, depicting number of nodes, endings and process length at different radial distance from soma (**B**; Sham: n = 10; Casp3: n = 7 mice; Two-way RM-ANOVA: *Nodes*: interaction F_6,90_ = 9.348, p < 0.0001; distance F_6,90_ = 192.4, p < 0.0001; lesion F_1,15_ = 11.57, p = 0.004; ****p < 0.0001 at 20-30 µm; *Endings*: interaction F_6,90_ = 12.39, p < 0 .0001; distance F_6,90_ = 261.4, p < 0.0001; lesion F_1,15_ = 10.65, p = 0.005; ****p < 0.0001 at 20-30 µm; *Length*: interaction F_6,90_ = 14.29, p < 0.0001; distance F_6,90_ = 416.9, p < 0.0001; lesion F_1,15_ = 14.62, p = 0.002; ****p < 0.0001 at 20-30 µm; all with Sidak’s multiple comparisons test), and the somatic perimeter and area (**C**) of Iba1^+^ cells in the hippocampus of male Sham and Casp3 mice (Sham: n = 10; Casp3: n = 7 mice). **D)** Representative confocal images and plot showing stereological cell count of GFAP^+^ cells in the hippocampus of female Sham and Casp3 mice (n = 8 mice / group). Nuclei are counterstained with DAPI (scale: 50 µm).  **E)** Images and plot showing hippocampal Iba1^+^ cell count in female Sham and Casp3 mice (n = 8 mice / group; Mann-Whitney test: ***p = 0.0003). Nuclei are counterstained with DAPI (scale: 50 µm). **F-H)** Representative images and 3D-reconstruction of microglia (**F**; scale: 10 μm) in female mice and Sholl analysis parameters, depicting number of intersections, nodes, endings and process length at different radial distance from soma (**G**; n = 6 mice / group; Two-way RM-ANOVA: *Intersections*: interaction F_6,60_ = 6.002, p < 0.0001; distance F_6,60_ = 380.6, p < 0.0001; lesion F_1,10_ = 14.58, p = 0.003; **p = 0.006 at 10 µm, ****p < 0.0001 at 20 µm, **p = 0.003 at 30 µm; *Nodes*: interaction F_6,60_ = 6.222, p < 0.0001; distance F_6,60_ = 287.9, p < 0.0001; lesion F_1,10_ = 23.55, p = 0.0007; ****p < 0.0001 at 20-30 µm; *Endings*: interaction F_6,60_ = 7.968, p < 0.0001; distance F_6,60_ = 407.7, p < 0.0001; lesion F_1,10_ = 26.20, p = 0.0005; ****p < 0.0001 at 20-30 µm, **p = 0.009 at 40 µm; *Length*: interaction F_6,60_ = 10.69, p < 0.0001; distance F_6,60_ = 549.9, p < 0.0001; lesion F_1,10_ = 24.33, p = 0.0006; ****p < 0.0001 at 20-30 µm; all with Sidak’s multiple comparisons test) and the somatic perimeter and area (**H**) of Iba1^+^ cells in the hippocampus of female Sham and Casp3 mice (n = 6 mice / group). **I)** Confocal images of NLRP3 and Iba1 immunostaining (scale: 15 µm) in female mice. The plots show: % of NLRP3^+^/Iba1^+^ cells (Sham: n = 5; Casp3: n = 6 mice. Unpaired *t*-test: *p = 0.048) and NLRP3 levels (Unpaired *t*-test: *p = 0.018). **J)** Confocal images and plot of hippocampal IL-1β levels (scale: 15 µm) in microglia from female mice (Sham: n = 6; Casp3: n = 7 mice; Welch’s *t*-test: *p = 0.039). Overall, open symbols refer to females, filled symbols refer to males.

**
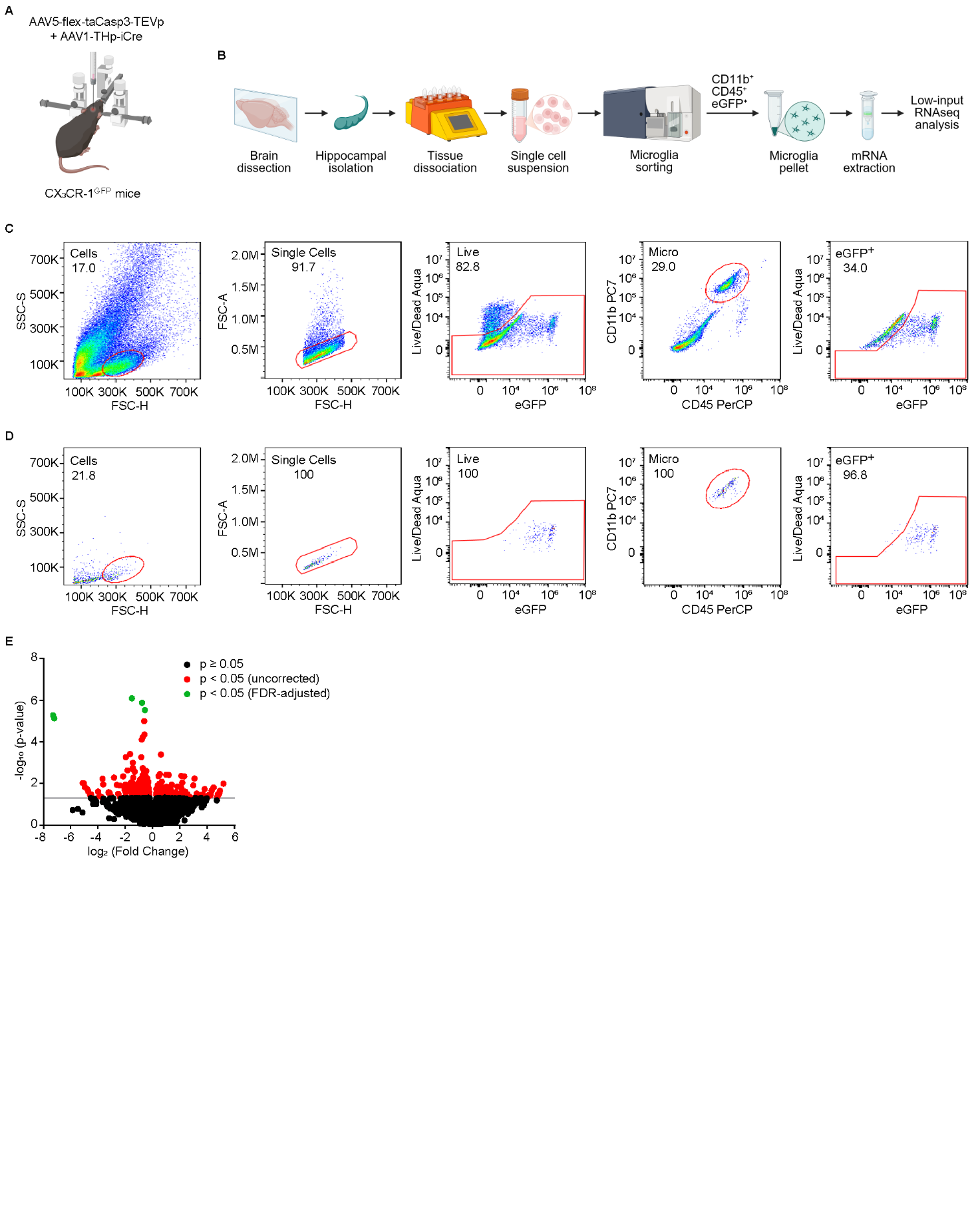
**

**Supplemental Figure 4: Additional analysis of RNA sequencing in hippocampal microglia from Sham and Casp3 CX_3_CR-1^GFP^ mice.**

**A)** CX_3_CR-1^GFP^ mice expressing the GFP protein in microglia were unilaterally infused in the midbrain with the AAV5-flex-taCasp3-TEVp + AAV1-THp-iCre mix and analysed 1-month post-lesion. **B)** Scheme of the experimental procedure used for the isolation of hippocampal microglia using the CD11b^+^-CD45^+^-eGFP^+^ gating strategy. **C-D)** Flow-cytometry gating strategy for microglia isolation (**C**) and purity check (**D**). From left to right: the gating on the general cell population was based on size (FSC-H) and granularity (SSC-S); FSC-A *vs* FSC-H “Single Cells” Isolating single cells by removing doublets or clumps; eGFP *vs* live dead Aqua: “Live” Identifying live cells using a viability dye (live dead Aqua); CD45 *vs* CD11b “Micro” Identifying microglia or myeloid cells (CD11b^high^, CD45^mid^); eGFP *vs* live dead Aqua: “eGFP^+^” Identifying cells expressing microglia eGFP^+^. **E**) Volcano plots illustrating the magnitude (as log_2_ fold change) and statistical significance (as -log_10_ p-values) of changes in the expression of individual genes in hippocampal microglia of lesioned relative to Sham mice. [Figure created using BioRender.com].

**
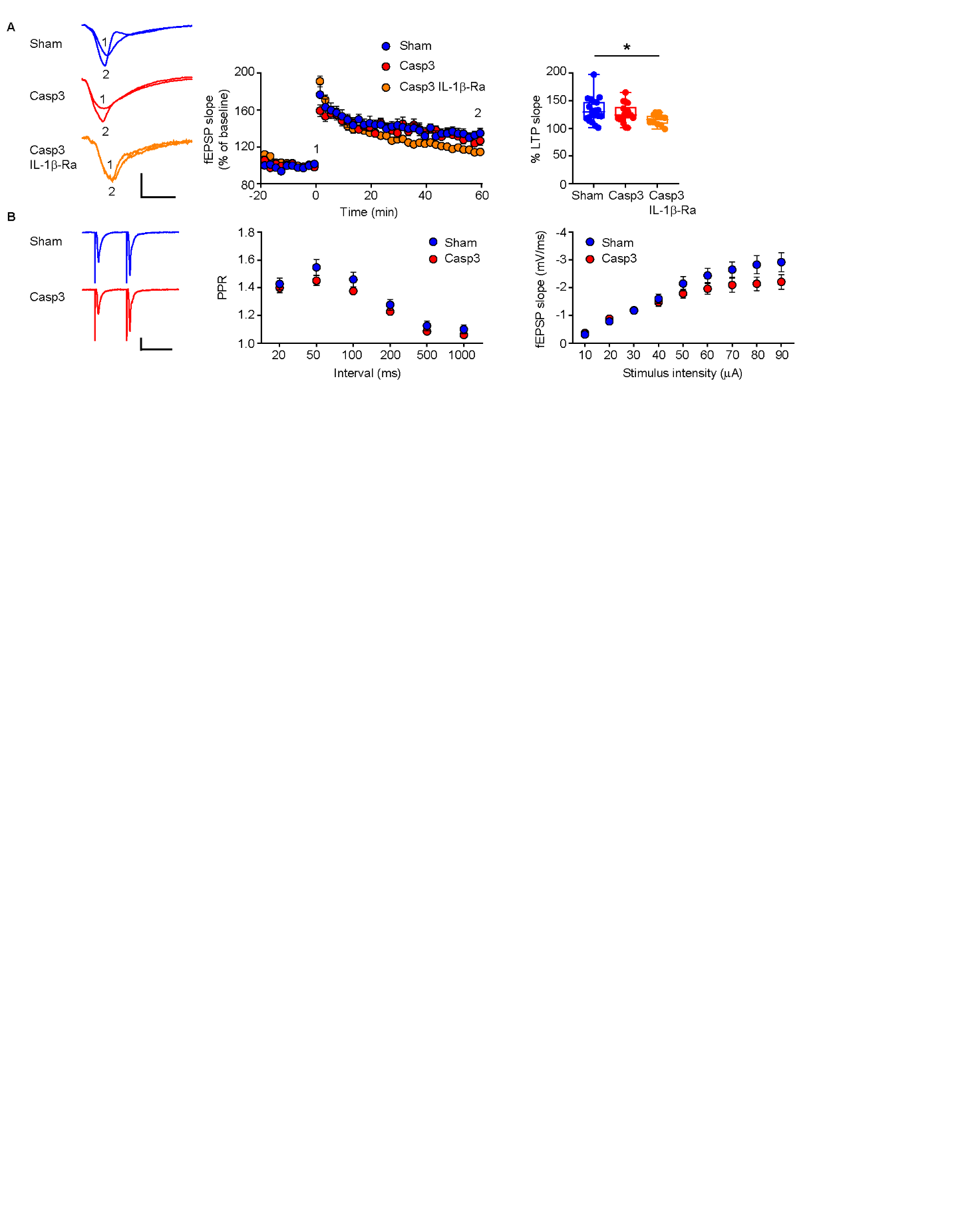
**

**Supplemental Figure 5: Analysis of synaptic plasticity in Sham and Casp3 mice.**

**A)** Representative traces showing *f*EPSPs at half maximal stimulation during baseline and 1 h after the conditioning train (scale: 5 ms; 1 mV) and time-course (mean ± s.e.m.) of normalized, average *f*EPSP slope from Sham, Casp3 and Casp3 slices treated with IL-1β-Ra. The plot shows pooled data for *f*EPSP normalized slope (Sham: n = 20 slices / 10 mice; Casp3: n = 18 slices / 9 mice; Casp3 IL-1β-Ra: n = 12 slices / 5 mice. Kruskal-Wallis: p = 0.038; Sham *vs* Casp3 IL-1β-Ra: *p = 0.023, with Dunn’s multiple comparisons test). **B)** Representative *f*EPSP traces of paired responses to half maximal stimulation of Shaffer collateral fibers from Sham and Casp3 slices at 50 ms interval (scale: 50 ms; 1 mV). The plots show (*left*) the PPR at different stimulus intervals (Sham: n = 19 slices / 6 mice; Casp3: n = 17 slices / 5 mice. Two-way RM-ANOVA interaction F_5,170_ = 0.7433, p = 0.592; interval F_2648,90_ = 128.7, p < 0.0001; lesion F_1,34_ = 1.636, p = 0.210) and (*right*) the I/O curves at different stimulation intensities (Sham: n = 21 slices / 12 mice; Casp3: n = 18 slices / 8 mice. Two-way RM-ANOVA interaction F_8,296_ = 3.575, p = 0.0006; interval F_1430,53_ = 86.65, p < 0.0001; lesion F_1,37_ = 1.529, p = 0.224).

**
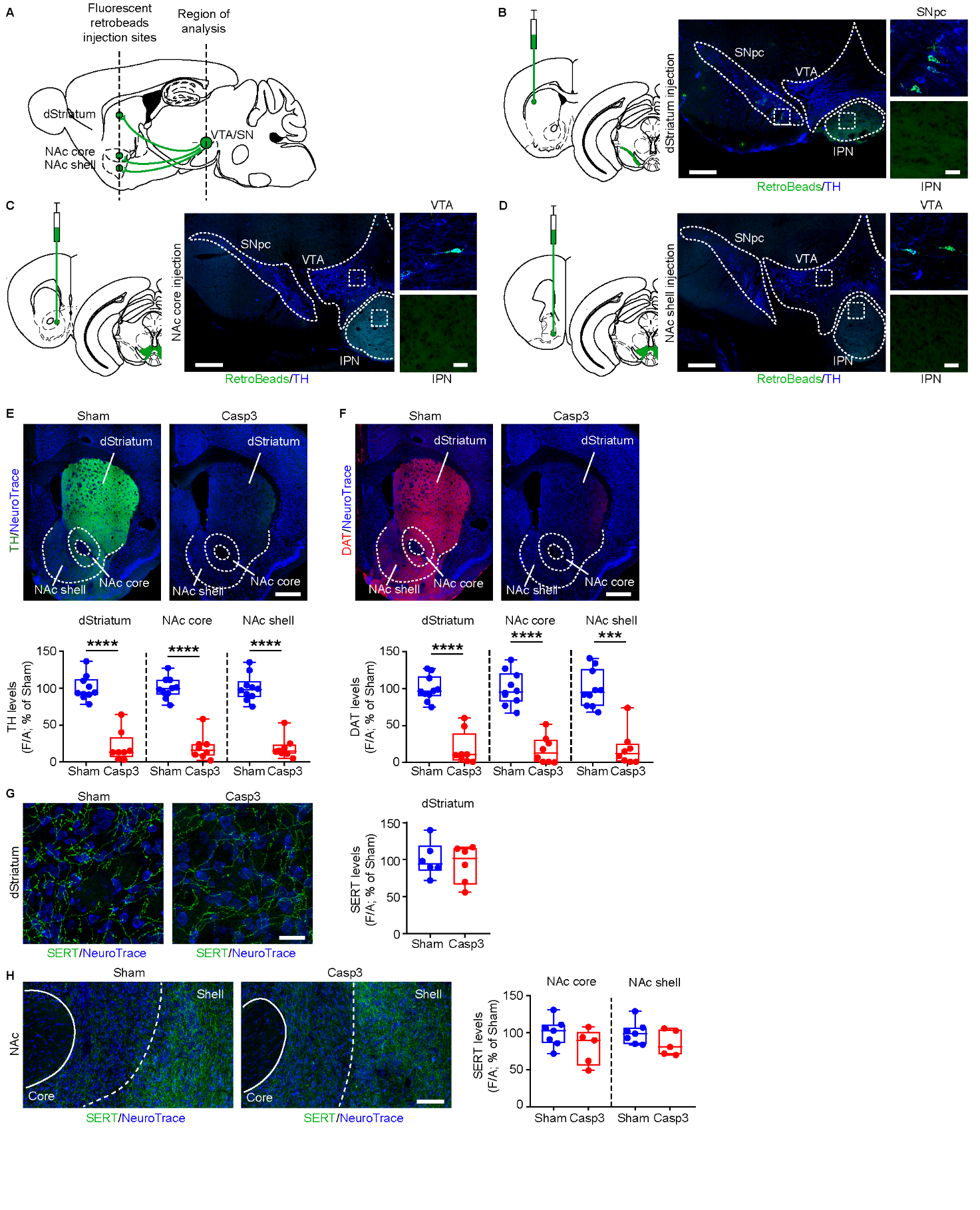
**

**Supplemental Figure 6: Evaluation of dopaminergic and serotonergic innervation in the dorsal striatum and NAc of Sham and Casp3 mice.**

**A)** Sagittal brain map of the Retrobead-injection sites and region of analyses. **B-D)** Brain map and confocal images of retrogradely labeled midbrain neurons from coronal sections containing VTA, SNpc and IPN (TH; scale: 250 μm). The small panels show Z-stack image of TH^+^ cells in the SNpc, VTA and IPN labelled with Retrobeads from dorsal striatum (**B**), NAc core (**C**) and NAc shell (**D**; scale: 25 μm). **E-H)** Representative confocal images and densitometric analysis (expressed as % of Sham levels) of **E)** TH (Sham: n = 10, Casp3: n = 8 mice. *Dorsal striatum*: Mann-Whitney test: ****p < 0.0001; *NAc core*: Unpaired *t*-test: ****p < 0.0001; *NAc shell*: Mann-Whitney test: ****p < 0.0001), **F)** DAT (Sham: n = 10, Casp3: n = 8 mice. *Dorsal striatum*: Mann-Whitney test: ****p < 0.0001; *NAc core*: Unpaired *t*-test: ****p < 0.0001; *NAc shell*: Mann-Whitney test: ***p < 0.0001), and **G-H)** SERT (*Dorsal striatum*: n = 6 mice / group. *NAc core* and *NAc shell*: Sham: n = 7, Casp3: n = 5 mice) fibers in the dorsal striatum (**G**) and NAc core and shell (**H**) of Sham and Casp3 mice (neurons are counterstained with NeuroTrace; scale: **E-F** 500 µm; **G** 25 µm; **H** 100 µm).

**
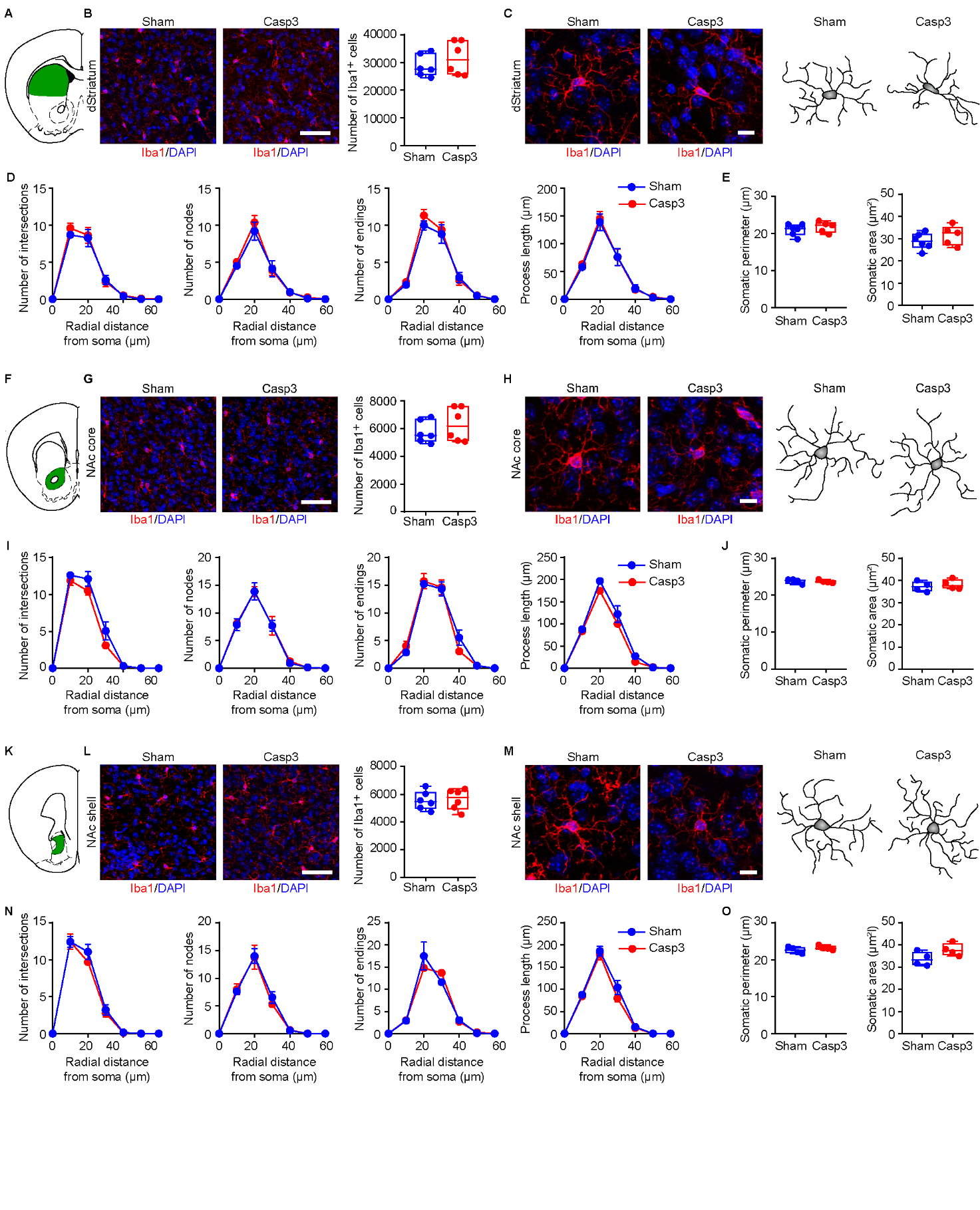
**

**Supplemental Figure 7: Analysis of microglia in the dorsal and ventral striatum of Sham and Casp3 mice.**

**A)** Schematic representation of the dorsal striatum in coronal sections. **B)** Representative confocal images and plot showing stereological cell count of Iba1^+^ cells in the dorsal striatum (scale: 50 µm) of Sham and Casp3 mice (Sham: n = 6, Casp3: n = 6 mice). Nuclei are counterstained with DAPI. **C-E)** Representative images and 3D-reconstruction of microglia (Iba1) in the dorsal striatum of Sham and Casp3 mice (**C**; scale: 10 μm). The graphs show the morphological complexity of microglia cells, depicted by number of intersections, nodes, endings and process length of microglia cell, as the radial distance from soma (**D**), and the somatic perimeter and area (**E**; Sham: n = 6; Casp3: n = 5 mice; Two-way RM-ANOVA *Intersection:* interaction F_6,54_ = 0.347, p = 0.909; distance F_6,54_ = 166.4, p < 0.0001; lesion F_1,9_ = 0.124, p = 0.733; *Nodes*: interaction F_6,54_ = 0.445, p = 0.845; distance F_6,54_ = 105.5, p < 0.0001; lesion F_1,9_ = 0.202, p = 0.664; *Endings*: interaction F_6,54_ = 0.564, p = 0.757; distance F_6,54_ = 147.9, p < 0.0001; lesion F_1,9_ = 0.285, p = 0.606; *Length*: interaction F_6,54_ = 0.108, p = 0.995; distance F_6,54_ = 121.1, p < 0.0001; lesion F_1,9_ = 0.034, p = 0.857). **F)** Schematic representation of the NAc core in coronal sections. **G)** Representative confocal images and relative plot showing stereological cell count of Iba1^+^ cells in the NAc core (scale: 50 µm) of Sham and Casp3 mice (Sham: n = 6, Casp3: n = 6 mice). Nuclei are counterstained with DAPI. **H-J)** Representative images and 3D-reconstruction of microglia (Iba1) in the NAc core of Sham and Casp3 mice (**H**; scale: 10 μm). The graphs show the morphological complexity of microglia cells, depicted by number of intersections, nodes, endings and process length of microglia cell, as the radial distance from soma (**I**), and the somatic perimeter and area (**J**; n = 4 mice / group; Two-way RM-ANOVA *Intersection:* interaction F_6,36_ = 1.610, p = 0.173; distance F_6,36_ = 273.0, p < 0.0001; lesion F_1,6_ = 2.950, p = 0.137; *Nodes*: interaction F_6,36_ = 0.019, p > 0.999; distance F_6,36_ = 109.0, p < 0.0001; lesion F_1,6_ = 0.015, p = 0.907; *Endings*: interaction F_6,36_ = 1.240, p = 0.310; distance F_6,36_ = 172.5, p < 0.0001; lesion F_1,6_ = 0.021, p = 0.889; *Length*: interaction F_6,36_ = 1.357, p = 0.258; distance F_6,36_ = 267.4, p < 0.0001; lesion F_1,6_ = 3.700, p = 0.103). **K)** Schematic representation of NAc shell in coronal sections. **L)** Representative confocal images and relative plot showing stereological cell count of Iba1^+^ cells in the NAc shell (scale: 50 µm) of Sham and Casp3 mice (Sham: n = 6, Casp3: n = 6 mice). Nuclei are counterstained with DAPI. **M-O)** Representative images and 3D-reconstruction of microglia (Iba1) in the NAc shell of Sham and Casp3 mice (**M**; scale: 10 μm). The graphs show the morphological complexity of microglia cells, depicted by number of intersections, nodes, endings and process length of microglia cell, as the radial distance from soma (**N**), and the somatic perimeter and area (**O**; n = 4 mice / group; Two-way RM-ANOVA *Intersection:* interaction F_6,36_ = 0.532, p = 0.780; distance F_6,36_ = 231.2, p < 0.0001; lesion F_1,6_ = 0.702, p = 0.434; *Nodes*: interaction F_6,36_ = 0.247, p = 0.957; distance F_6,36_ = 104.7, p < 0.0001; lesion F_1,6_ = 0.056, p = 0.801; *Endings*: interaction F_6,36_ = 0.557, p = 0.762; distance F_6,36_ = 87.87, p < 0.0001; lesion F_1,6_ = 0.010, p = 0.923; *Length*: interaction F_6,36_ = 0.876, p = 0.522; distance F_6,36_ = 216.1, p < 0.0001; lesion F_1,6_ = 1.299, p = 0.144).

**
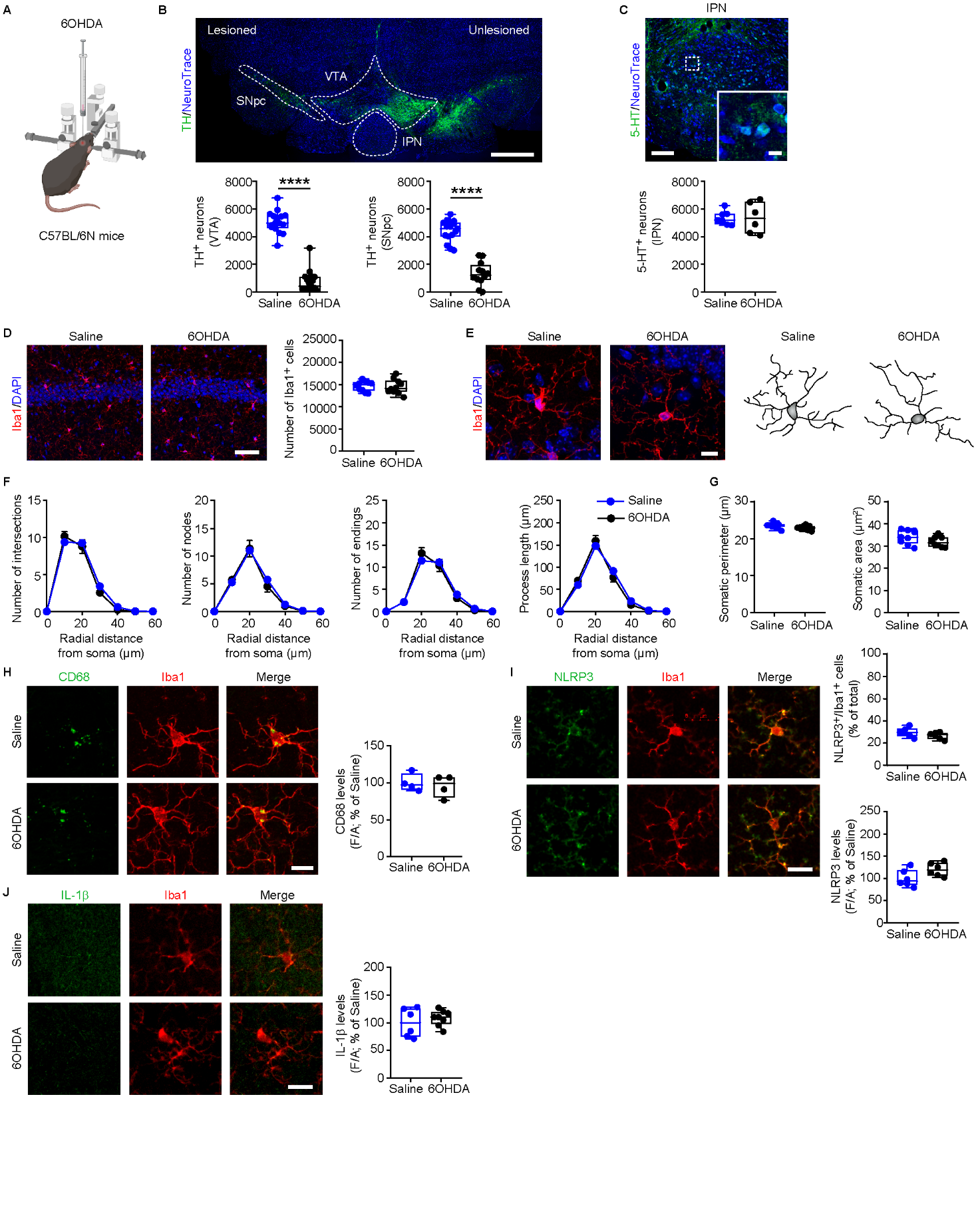
**

**Supplemental Figure 8: Validation of dopaminergic lesion and evaluation of hippocampal neuroinflammation in 6OHDA mice.**

**A)** Unilateral infusion of 6OHDA or saline into the left midbrain under stereotactic control in C57BL/6N mice. **B)** Representative immunofluorescence of TH^+^ neurons, counterstained with NeuroTrace, in the midbrain of 6OHDA mice (scale: 500 µm) and stereological cell count of TH^+^ neurons in the ipsilateral VTA (*left*; Saline: n = 16; 6OHDA: n = 17 mice. Mann-Whitney test: ****p < 0.0001) and SNpc (*right*; Saline: n = 15; 6OHDA: n = 12 mice. Unpaired *t*-test: ****p < 0.0001). **C)** Representative confocal images of IPN stained with 5-HT and NeuroTrace in 6OHDA mice (scale: 100 μm; inset, 10 µm), and stereological cell count of 5-HT^+^ neurons in 6OHDA mice (Saline: n = 7; 6OHDA: n = 6 mice). **D)** Confocal images and plot showing cell count of Iba1^+^ cells in Saline and 6OHDA hippocampi (n = 10 mice / group). Nuclei are counterstained with DAPI (scale: 50 µm). **E)** Representative confocal images and 3D-reconstruction of microglia (Iba1; scale: 10 μm). **F-G)** The graphs show Sholl analysis of microglia cells, depicting number of intersections, nodes, endings and length at different radial distances from soma (**F**; Saline: n = 9; 6OHDA: n = 8 mice; Two-way RM-ANOVA: *Intersections*: interaction F_6,90_ = 1.461, p = 0.201; distance F_6,90_ = 422.1, p < 0.0001; lesion F_1,15_ = 0.223, p = 0.644; *Nodes*: interaction F_6,90_ = 0.738, p = 0.621; distance F_6,90_ = 181.9, p < 0.0001; lesion F_1,15_ = 0.041, p = 0.843; *Endings*: interaction F_6,90_ = 1.651, p = 0.142; distance F_6,90_ = 260.5, p < 0.0001; lesion F_1,15_ = 0.002, p = 0.964; *Length*: interaction F_6,90_ = 2.522, p = 0.027; distance F_6,90_ = 412.6, p < 0.0001; lesion F_1,15_ = 0.008, p = 0.930) and somatic area and perimeter (**G**). **H)** Confocal images of CD68 and Iba1 (scale: 10 µm). The plot shows CD68 immunostaining intensity in microglia cell body (n = 4 mice / group). **I)** Confocal images of NLRP3 and Iba1 (scale: 15 µm). The plots show: (*top*) the % of NLRP3^+^/Iba1^+^ cells and (*bottom*) NLRP3 levels (n = 6 mice / group). **J)** Confocal images of IL-1β and Iba1 (scale: 15 µm) and plot of hippocampal IL-1β levels (Saline: n = 6; 6OHDA: n = 8 mice). [Figure created using BioRender.com].

**
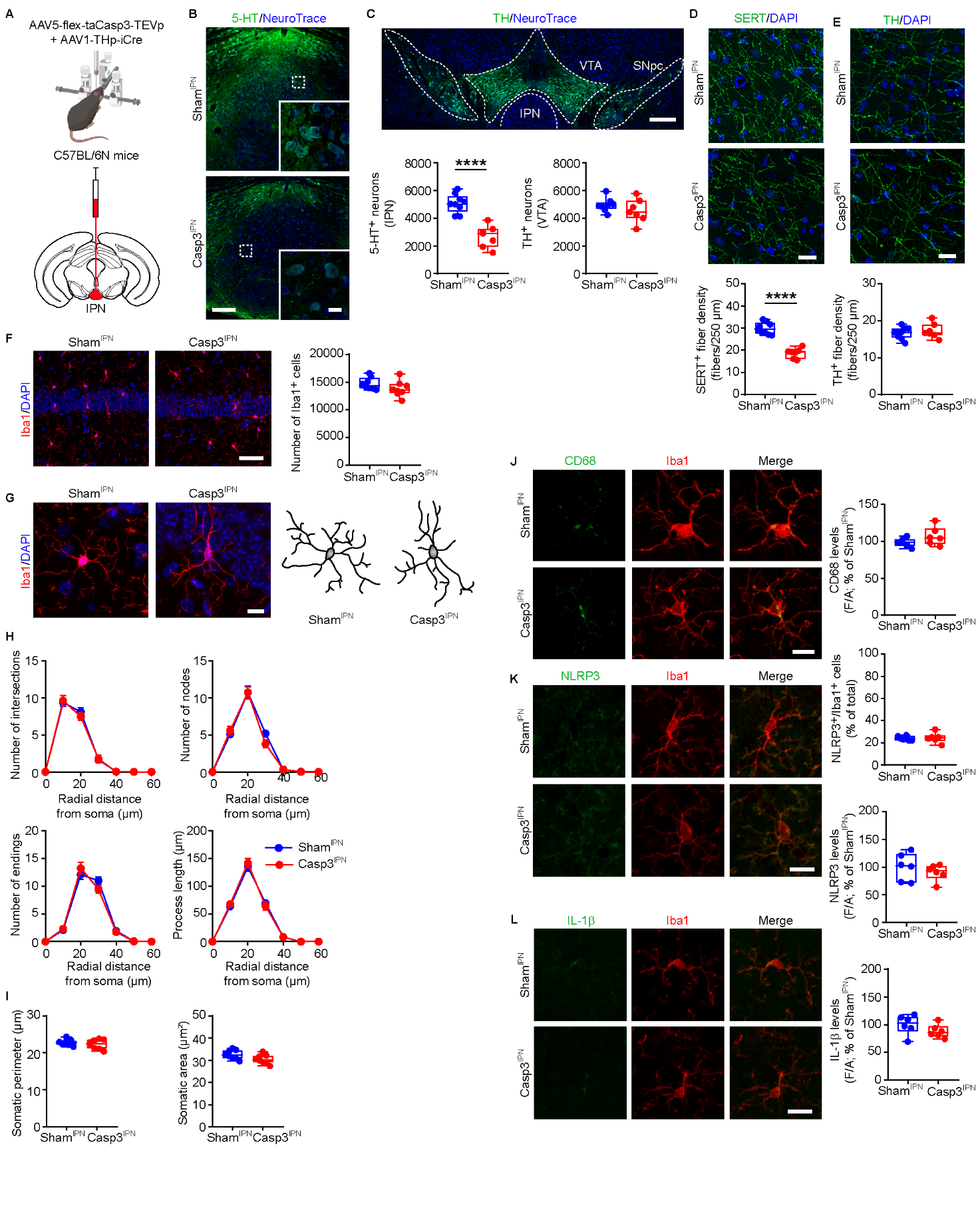
**

**Supplemental Figure 9: Validation of serotonergic lesion and evaluation of hippocampal neuroinflammation in Casp3^IPN^ mice.**

**A)** Unilateral infusion of AAV5-flex-taCasp3-TEVp and AAV1-THp-iCre into the IPN under stereotactic control in C57BL/6N mice. **B)** Representative confocal images of IPN stained with 5-HT and NeuroTrace in Sham^IPN^ and Casp3^IPN^ mice (scale: 100 μm; inset, 10 µm), and stereological cell count of 5-HT^+^ neurons in the total IPN (Sham^IPN^: n = 9; Casp3^IPN^: n = 7 mice. Unpaired *t*-test: ****p < 0.0001). **C)** Representative immunofluorescence of TH^+^ neurons, counterstained with NeuroTrace, in the midbrain of Casp3^IPN^ mice (scale: 250 µm) and stereological cell count of TH^+^ neurons in the ipsilateral VTA (Sham^IPN^: n = 9; Casp3^IPN^: n = 7 mice). **D-E)** Confocal images and plots showing **D)** SERT^+^ (Sham^IPN^: n = 9; Casp3^IPN^: n = 7 mice. Unpaired *t*-test: ****p < 0.0001) and **E)** TH^+^ (Sham^IPN^: n = 9; Casp3^IPN^: n = 7 mice) hippocampal fiber density (expressed as fibers / 250 μm; scale: 20 µm). Nuclei are counterstained with DAPI. **F)** Confocal images and plot showing cell count of Iba1^+^ cells in Sham^IPN^ and Casp3^IPN^ hippocampi (n = 8 mice / group). Nuclei are counterstained with DAPI (scale: 50 µm). **G-I)** Representative confocal images and 3D-reconstruction of microglia (scale: 10 μm), and graphs showing Sholl analysis of microglia cells, depicting number of intersections, nodes, endings and length at different radial distances from soma (**H**; n = 8 mice / group; Two-way RM-ANOVA: *Intersections*: interaction F_6,84_ = 0.312, p = 0.929; distance F_6,90_ = 296, p < 0.0001; lesion F_1,14_ = 0.041, p = 0.842; *Nodes*: interaction F_6,84_ = 1.1, p = 0.369; distance F_6,90_ = 216.6, p < 0.0001; lesion F_1,14_ = 0.041, p = 0.842; *Endings*: interaction F_6,84_ = 1.390, p = 0.228; distance F_6,90_ = 249.4, p < 0.0001; lesion F_1,14_ = 0.035, p = 0.853; *Length*: interaction F_6,84_ = 0.358, p = 0.904; distance F_6,90_ = 302.6, p < 0.0001; lesion F_1,14_ = 0.042, p = 0.841) and somatic area and perimeter (**I**). **J)** Confocal images of CD68 and Iba1 (scale: 10 µm). The plot shows CD68 immunostaining intensity in microglia cell body (n = 6 mice / group). **K)** Confocal images of NLRP3 and Iba1 (scale: 15 µm). The plots show: (*top*) the % of NLRP3^+^/Iba1^+^ cells and (*bottom*) NLRP3 levels (n = 6 mice / group). **L)** Confocal images of IL-1β and Iba1 (scale: 15 µm) and plot of hippocampal IL-1β levels (n = 6 mice / group). [Figure created using BioRender.com].

**
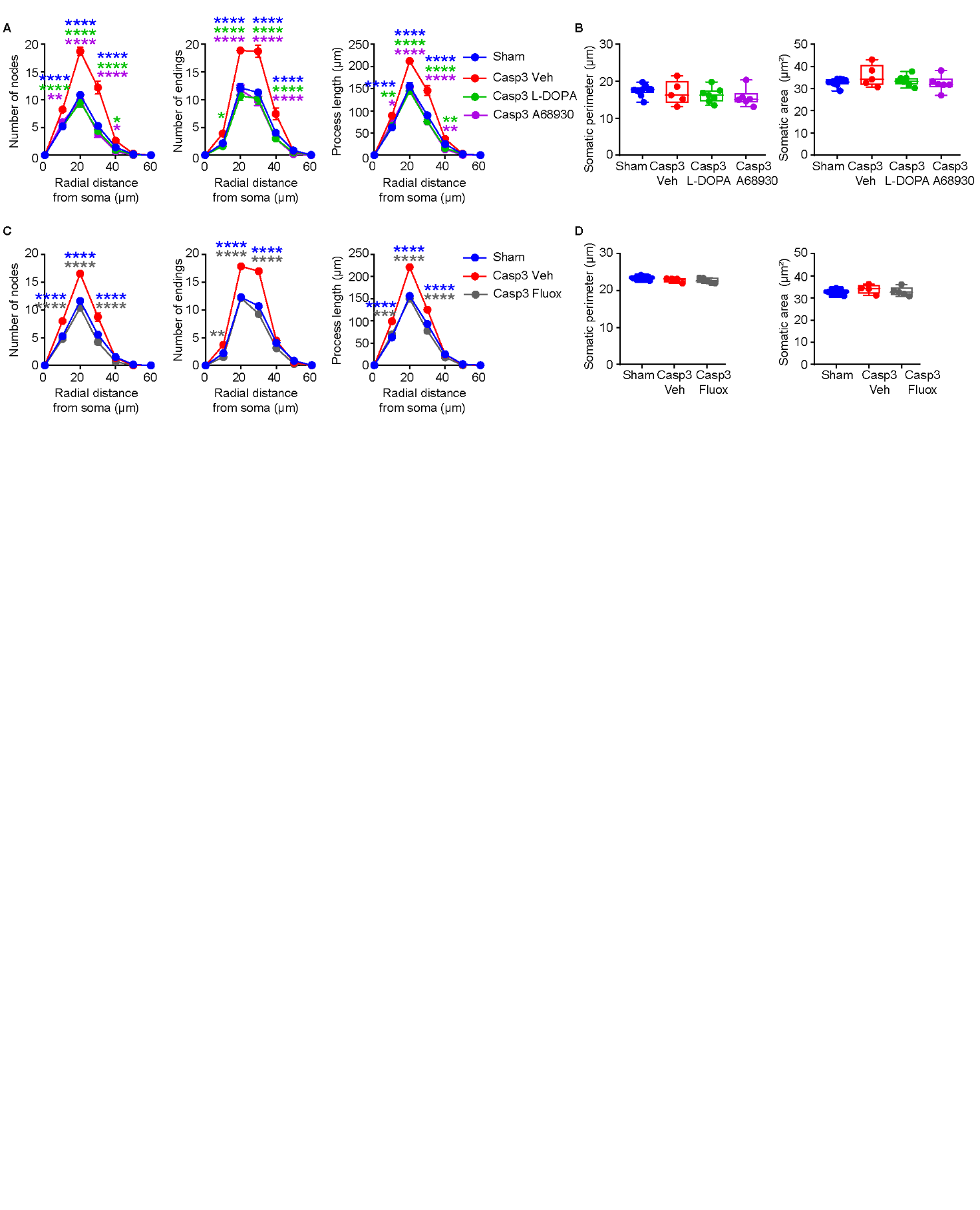
**

**Supplemental Figure 10:** **Additional analysis of neuroinflammation in the hippocampus of Casp3 mice following treatments.**

**A)** Graphs show Sholl analysis parameters at different radial distance from soma in Sham and Casp3 mice treated with Veh, L-DOPA or A68930 (Sham: n = 10; Casp3 Veh: n = 5, Casp3 L-DOPA: n = 7, Casp3 A68930: n = 6 mice. Two-way RM-ANOVA: *Nodes*: interaction F_18,144_ = 22.63, p < 0.0001; distance F_6,144_ = 690.4, p < 0.0001; treatment F_3,24_ = 27.65, p < 0.0001; Sham *vs* Casp3 Veh (blue): ****p < 0.0001 at 10-30 µm; Casp3 Veh *vs* Casp3 L-DOPA (green): ****p < 0.0001 at 10-30 µm, *p = 0.048 at 40 µm; Casp3 Veh *vs* Casp3 A68930 (purple): **p = 0.003 at 10 µm, ****p < 0.0001 at 20-30 µm, *p = 0.018 at 40 µm; *Endings*: interaction F_18,144_ = 13.33, p < 0.0001; distance F_6,144_ = 680.3, p < 0.0001; treatment F_3,24_ = 22.21, p < 0.0001; Sham *vs* Casp3 Veh (blue): ****p < 0.0001 at 20-40 µm; Casp3 Veh *vs* Casp3 L-DOPA (green): *p = 0.020 at 10 µm, ****p < 0.0001 at 20-40 µm; Casp3 Veh *vs* Casp3 A68930 (purple): ****p < 0.0001 at 20-40 µm; *Length*: interaction F_18,144_ = 13.10, p < 0.0001; distance F_6,144_ = 1200, p < 0.0001; treatment F_3,24_ = 18.41, p < 0.0001; Sham *vs* Casp3 Veh (blue): ****p < 0.0001 at 10-30 µm; Casp3 Veh *vs* Casp3 L-DOPA (green): **p = 0.001 at 10 µm, ****p < 0.0001 at 20-30 µm, **p = 0.008 at 40 µm; Casp3 Veh *vs* Casp3 A68930 (purple): *p = 0.049 at 10 µm, ****p < 0.0001 at 20-30 µm, **p = 0.003 at 40 µm; all with Tukey’s multiple comparisons test). **B)** Plots show somatic perimeter and area of microglia cells from Sham and Casp3 mice treated with vehicle, L-DOPA or A68930 (Sham: n = 10, Casp3 Veh: n = 5, Casp3 L-DOPA: n = 7, Casp3 A68930: n = 6 mice. *Perimeter*: One-Way ANOVA: F_3,24_ =1.032; p = 0.396; *Area*: One-Way ANOVA: F_3,24_ =1.425; p = 0.260, all with Tukey’s multiple comparisons test). **C)** Graphs show Sholl analysis parameters at different radial distance from soma in Sham and Casp3 mice treated with Veh or Fluox (Sham: n = 10, Casp3 Veh: n = 4, Casp3 Fluox: n = 5 mice. Two-way RM-ANOVA: *Nodes*: interaction F_12,96_ = 15.88, p < 0.0001; distance F_6,96_ = 733.4, p < 0.0001; treatment F_2,16_ = 26.84, p < 0.0001; Sham *vs* Casp3 Veh (blue): ****p < 0.0001 at 10-30 µm; Casp3 Veh *vs* Casp3 Fluox (grey): ****p < 0.0001 at 10-30 µm; *Endings*: interaction F_12,96_ = 24.16, p < 0.0001; distance F_6,96_ = 1026, p < 0.0001; treatment F_2,16_ = 31.80, p < 0.0001; Sham *vs* Casp3 Veh (blue): ****p < 0.0001 at 20-30 µm; Casp3 Veh *vs* Casp3 Fluox (grey): **p = 0.004 at 10 µm; ****p < 0.0001 at 20-30 µm; *Length*: interaction F_12,96_ = 17.13, p < 0.0001; distance F_6,96_ = 1060, p < 0.0001; treatment F_2,16_ = 18.25, p < 0.0001; Sham *vs* Casp3 Veh (blue): ****p < 0.0001 at 10-30 µm; Casp3 Veh *vs* Casp3 Fluox (grey): ***p = 0.0003 at 10-30 µm, ****p < 0.0001 at 20-30 µm; all with Sidak’s multiple comparisons test). **D)** Plots show somatic perimeter and area of microglia cells from Sham and Casp3 mice treated with Veh or Fluox (Sham: n = 10, Casp3 Veh: n = 4, Casp3 Fluox: n = 5 mice; *Perimeter*: One-Way ANOVA: F_2,16_ = 3.265; p = 0.065; *Area*: One-Way ANOVA: F_2,16_ = 0.844; p = 0.448, all with Tukey’s multiple comparisons test).

**
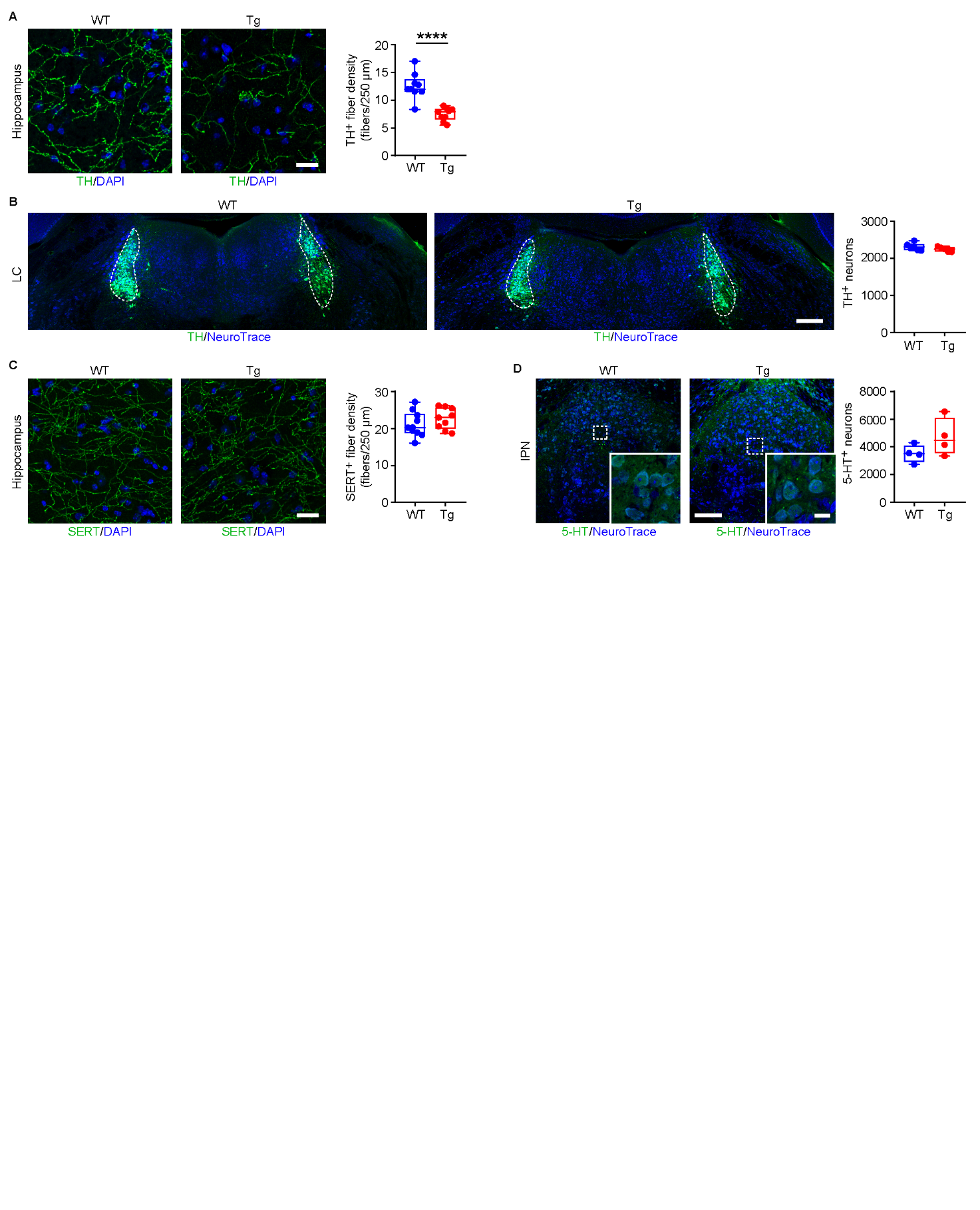
**

**Supplemental Figure 11: Characterization of hippocampal monoaminergic innervation in 7-month-old WT and Tg2576 mice.**

**A)** Representative confocal images and plot showing the density (expressed as fibers / 250 μm) of TH^+^ (n = 9 mice / group. Unpaired *t*-test: ****p < 0.0001) fibers in the dorsal hippocampus of WT and Tg2576 (Tg) mice (scale: 20 µm). Nuclei are counterstained with DAPI. **B)** Confocal images of TH^+^ neurons, counterstained with NeuroTrace, in the LC of WT and Tg mice (scale: 200 µm). The plot shows stereological cell count of LC TH^+^ neurons (WT: n = 6; Tg: n = 5 mice). **C)** Representative confocal images and plot showing fiber density (expressed as fibers / 250 μm) of serotonergic (SERT; WT: n = 10; Tg: n = 9 mice) fibers in the hippocampus of WT and Tg mice (scale: 20 µm). Nuclei are counterstained with DAPI. **D)** Representative confocal images of IPN stained with 5-HT and NeuroTrace (scale: 100 μm; inset, 10 µm) and plot showing stereological count of 5-HT^+^ neurons in the IPN from WT and Tg mice (n = 4 mice / group).

**
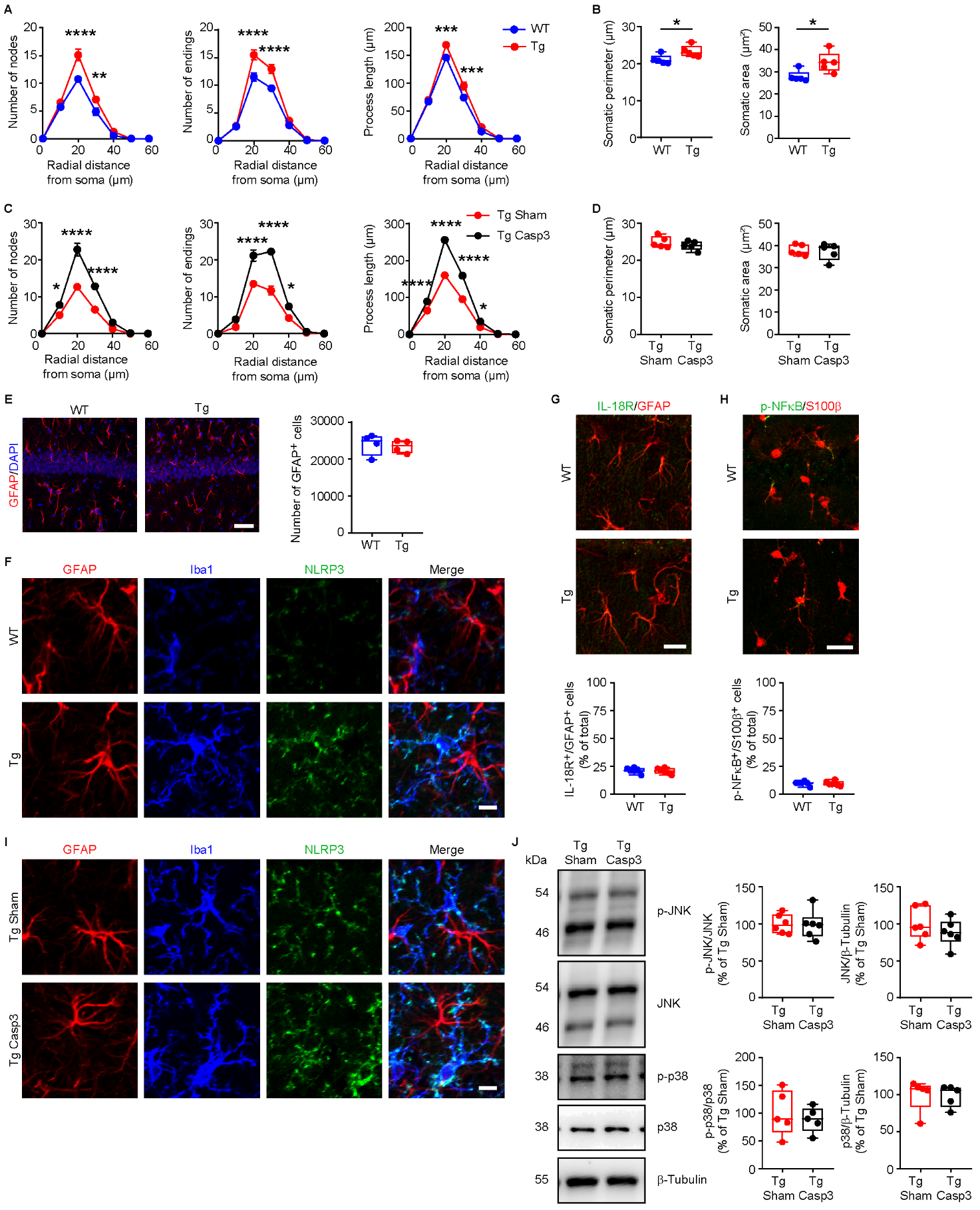
**

**Supplemental Figure 12. Additional analysis of neuroinflammation in WT, Tg2576 naïve, and in Tg Sham and Tg2576 Casp3 mice.**

**A-B)** Graphs show Sholl analysis parameters of Iba1^+^ cells at different radial distance from soma in WT and Tg mice (A; n = 5 mice / group. Two-way RM-ANOVA: *Nodes*: interaction F_6,48_ = 8.133, p < 0.0001; distance F_6,48_ = 298.2, p < 0.0001; genotype F_1,8_ = 21.22, p = 0.002; ****p < 0.0001 at 20 µm; **p = 0.003 at 30 µm; *Endings*: interaction F_6,48_ = 6.950, p < 0.0001; distance F_6,48_ = 295.0, p < 0.0001; genotype F_1,8_ = 38.40, p = 0.0004; ****p < 0.0001 at 20-30 µm; *Length*: interaction F_6,48_ = 4.165, p = 0.002; distance F_6,48_ = 587.7, p < 0.0001; genotype F_1,8_ = 14.82, p = 0.005; ***p = 0.0002 at 20 µm; ***p = 0.0006 at 30 µm; all with Sidak’s multiple comparisons test), and plots show somatic perimeter and area of microglia cells WT and Tg mice (**B**; *Perimeter*: Unpaired *t*-test: *p = 0.037; *Area*: Mann-Whitney test: *p = 0.032). **C-D)** Graphs show Sholl analysis parameters of Iba1^+^ cells at different radial distance from soma in Tg Sham and Tg Casp3 mice (**C**; n = 5 mice / group. Two-way RM-ANOVA: *Nodes*: interaction F_6,48_ = 21.93, p < 0.0001; distance F_6,48_ = 206.7, p < 0.0001; lesion F_1,8_ = 25.65, p = 0.001; *p = 0.048 at 10 µm; ****p < 0.0001 at 20-30 µm; *Endings*: interaction F_6,48_ = 21.48, p < 0.0001; distance F_6,48_ = 303.5, p < 0.0001; lesion F_1,8_ = 31.27, p = 0.0005; ****p < 0.0001 at 20-30 µm; *p = 0.023 at 40 µm; *Length*: interaction F_6,48_ = 69.42, p < 0.0001; distance F_6,48_ = 1286, p < 0.0001; lesion F_1,8_ = 138.6, p < 0.0001; ****p < 0.0001 at 10-30 µm; *p = 0.018 at 40 µm; all with Sidak’s multiple comparisons test), and plots show somatic perimeter and area of microglia cells (**D**) in Tg Sham and Tg Casp3 mice. **E)** Representative confocal images and plot showing stereological cell count of GFAP^+^ cells in the hippocampus of WT and Tg2576 mice (n = 4 mice / group; scale: 50 µm). Nuclei are counterstained with DAPI. **F)** Representative confocal images of GFAP^+^ cells, Iba1, and NLRP3 in the hippocampus of WT and Tg2576 mice (n = 3 mice / group; scale: 10 µm). **G)** Representative confocal images of GFAP and IL-18R, and plot showing the % of IL-18R^+^/GFAP^+^ cells in the hippocampus of WT and Tg2576 mice (n = 6 mice / group; scale: 20 µm). **H)** Representative confocal images of S100β and p-NFκB (scale bar: 25 µm), and plot showing the % of p-NFκB^+/^S100β^+^ cells in the hippocampus of WT and Tg2576 mice (n = 6 mice / group). **I)** Representative confocal images of GFAP^+^ cells, Iba1, and NLRP3 in the hippocampus of Tg Sham and Tg Casp3 mice (n = 3 mice / group; scale: 10 µm). **J)** Representative western blots from the hippocampus of Tg Sham and Tg Casp3 mice, and plot showing the levels (expressed as % of Tg Sham) of p-JNK/JNK and p-JNK/β-tubulin (*top*; n = 6 mice / group), p-p38/p38 and p38/β-tubulin (*bottom*; n = 5 mice / group). β-Tubulin was used as loading control.

**Supplemental Table 1. Analysis of hippocampal monoamine metabolites with HPLC.**

|  | **Sham (10)** | **Casp3 (11)** | **Statistic** |
| --- | --- | --- | --- |
| **DOPAC** | 2.71 ± 0.21 | 1.741 ± 0.24 | Unpaired *t*-test: **p = 0.007 |
| **HVA** | 10.71 ± 0.81 | 6.86 ± 0.80 | Unpaired *t*-test: **p = 0.003 |
| **5-HIAA** | 119.51 ± 9.60 | 63.66 ± 7.335 | Unpaired *t*-test: ***p = 0.0002 |
| **MOPEG** | 31.90 ± 2.75 | 29.46 ± 3.89 | Mann-Whitney test: p = 0.197 |

Data (mean ± s.e.m.) show the levels, expressed as pg per 20 μL of monoamine metabolites of DA (DOPAC and HVA), 5-HT (HIAA) and NE (MOPEG), analyzed by HPLC in the hippocampus of Sham and Casp3 mice. In parenthesis are the number of mice analyzed.

**Abbreviations:** DOPAC: [3,4-Dihydroxyphenylacetic acid;](https://en.wikipedia.org/wiki/3,4-Dihydroxyphenylacetic_acid) HVA: Homovanillic acid; 5-HIAA: 5-Hydroxy-indoleacetic acid; MOPEG: 3-methoxy-4-hydroxyphenylethyleneglycol.

**Supplemental Table 2. Differentially expressed genes in Casp3 vs Sham mice**

Differentially expressed genes (p value < 0.05) between Casp3 and Sham mice. Genes are indicated with Ensemble and Gene symbol IDs. The magnitude of difference is expressed by Log_2_ Fold Change. A Log_2_ Fold Change > 0 indicates gene up-regulation in Casp3 mice. A Log_2_ Fold Change < 0 indicates gene down-regulated in Casp3 mice**.**

| **Ensemble ID** | **Gene symbol ID** | **log_2_ Fold Change** | **p value** | **p adj** |
| --- | --- | --- | --- | --- |
| ENSMUSG00000091119 | Ccdc152 | -1,516464997 | 8,01E-07 | 0,012657811 |
| ENSMUSG00000035202 | Lars2 | -0,770003873 | 1,32E-06 | 0,012657811 |
| ENSMUSG00000101939 | Gm28438 | -0,568916317 | 2,92E-06 | 0,01869378 |
| ENSMUSG00000056054 | S100a8 | -7,28083894 | 5,23E-06 | 0,025050719 |
| ENSMUSG00000056071 | S100a9 | -7,192783147 | 7,32E-06 | 0,028077934 |
| ENSMUSG00000064337 | mt-Rnr1 | -0,617707026 | 9,94E-06 | 0,031783844 |
| ENSMUSG00000064339 | mt-Rnr2 | -0,607220406 | 4,45E-05 | 0,121858947 |
| ENSMUSG00000021666 | Gfm2 | -0,721262484 | 5,88E-05 | 0,140993549 |
| ENSMUSG00000083563 | Gm13340 | -0,800230247 | 7,64E-05 | 0,162703101 |
| ENSMUSG00000119459 |  | -1,652160662 | 0,00038571 | 0,707387783 |
| ENSMUSG00000027399 | Il1a | 0,594638614 | 0,000405781 | 0,707387783 |
| ENSMUSG00000096992 | Gm26788 | -1,967688202 | 0,000528958 | 0,790462236 |
| ENSMUSG00000069892 | 9930111J21Rik2 | -0,836099577 | 0,000535879 | 0,790462236 |
| ENSMUSG00000064390 | Rnu73b | -1,448760973 | 0,001004535 | 0,999933142 |
| ENSMUSG00002075795 | Gm54867 | -1,44989414 | 0,001737459 | 0,999933142 |
| ENSMUSG00000080365 | Gm25776 | -0,722328576 | 0,00180711 | 0,999933142 |
| ENSMUSG00000045932 | Ifit2 | -1,407249808 | 0,002165922 | 0,999933142 |
| ENSMUSG00000064542 | Gm22980 | -1,837933031 | 0,002285885 | 0,999933142 |
| ENSMUSG00000029076 | Sdf4 | -0,460161564 | 0,00247052 | 0,999933142 |
| ENSMUSG00000113647 | Gm47210 | -1,314345818 | 0,002721303 | 0,999933142 |
| ENSMUSG00000102070 | Gm28661 | -0,639156044 | 0,00275561 | 0,999933142 |
| ENSMUSG00000109279 | Gm45220 | 0,532737782 | 0,003460406 | 0,999933142 |
| ENSMUSG00000025006 | Sorbs1 | 0,995790725 | 0,003785654 | 0,999933142 |
| ENSMUSG00000005986 | Ankrd13d | 1,163285096 | 0,003868119 | 0,999933142 |
| ENSMUSG00000029455 | Aldh2 | -0,735235678 | 0,004054541 | 0,999933142 |
| ENSMUSG00000028461 | Ccdc107 | -0,524826299 | 0,004271054 | 0,999933142 |
| ENSMUSG00002076680 |  | 2,096940213 | 0,004311718 | 0,999933142 |
| ENSMUSG00000109005 | Gm45221 | 0,433688379 | 0,004384447 | 0,999933142 |
| ENSMUSG00000067199 | Frat1 | -2,15403409 | 0,004556274 | 0,999933142 |
| ENSMUSG00000041380 | Htr2c | 2,300059595 | 0,004573695 | 0,999933142 |
| ENSMUSG00002074853 |  | -2,818738706 | 0,005153352 | 0,999933142 |
| ENSMUSG00000079641 | Rpl39 | -0,45163995 | 0,005153474 | 0,999933142 |
| ENSMUSG00000098973 | Mir6236 | -1,420212822 | 0,005172567 | 0,999933142 |
| ENSMUSG00000110350 | Gm10252 | -0,511192801 | 0,005424634 | 0,999933142 |
| ENSMUSG00000028333 | Anp32b | -0,443067061 | 0,00554293 | 0,999933142 |
| ENSMUSG00000036887 | C1qa | -0,447201946 | 0,005588257 | 0,999933142 |
| ENSMUSG00000030786 | Itgam | -0,372733983 | 0,005650187 | 0,999933142 |
| ENSMUSG00000105175 | Gm43097 | 3,065581935 | 0,005731755 | 0,999933142 |
| ENSMUSG00000108677 | Gm44759 | -3,666703858 | 0,005836005 | 0,999933142 |
| ENSMUSG00000020153 | Ndufs7 | -0,91453175 | 0,006354575 | 0,999933142 |
| ENSMUSG00000019464 | Ptger1 | -0,900447463 | 0,007403927 | 0,999933142 |
| ENSMUSG00000062683 | Atp5g2 | -0,705405794 | 0,007438942 | 0,999933142 |
| ENSMUSG00000046330 | Rpl37a | -0,390405575 | 0,007638073 | 0,999933142 |
| ENSMUSG00000105842 | Gm43329 | 0,669691729 | 0,007976574 | 0,999933142 |
| ENSMUSG00000058558 | Rpl5 | -0,317183747 | 0,00821931 | 0,999933142 |
| ENSMUSG00000031897 | Psmb10 | -0,746615714 | 0,008363979 | 0,999933142 |
| ENSMUSG00000119227 | Gm25099 | -1,511942584 | 0,008424695 | 0,999933142 |
| ENSMUSG00000086324 | Gm15564 | -1,508435459 | 0,008792036 | 0,999933142 |
| ENSMUSG00000097817 | Gm26810 | -1,587820463 | 0,008904088 | 0,999933142 |
| ENSMUSG00000026109 | Tmeff2 | 2,166440155 | 0,008911894 | 0,999933142 |
| ENSMUSG00000053317 | Sec61b | -1,056079649 | 0,009314616 | 0,999933142 |
| ENSMUSG00000119770 | Gm25483 | -5,115828481 | 0,009424312 | 0,999933142 |
| ENSMUSG00000085527 | Gm15535 | -5,053515704 | 0,009646401 | 0,999933142 |
| ENSMUSG00000036905 | C1qb | -0,452226476 | 0,009688028 | 0,999933142 |
| ENSMUSG00000022216 | Psme1 | -0,510971537 | 0,010031762 | 0,999933142 |
| ENSMUSG00000038879 | Nipal2 | 5,1852716 | 0,010041803 | 0,999933142 |
| ENSMUSG00000027712 | Anxa5 | -0,605902661 | 0,010287482 | 0,999933142 |
| ENSMUSG00000102145 | Gm38056 | 0,472772437 | 0,010294941 | 0,999933142 |
| ENSMUSG00000102349 | Gm37376 | -0,553685038 | 0,010791771 | 0,999933142 |
| ENSMUSG00000028843 | Sh3bgrl3 | -0,585008688 | 0,010995952 | 0,999933142 |
| ENSMUSG00000032484 | Ngp | -3,61123788 | 0,011052631 | 0,999933142 |
| ENSMUSG00000100131 | Gm28439 | -0,594053201 | 0,011219201 | 0,999933142 |
| ENSMUSG00000037849 | Ifi206 | -2,000892049 | 0,011279213 | 0,999933142 |
| ENSMUSG00000055421 | Pcdh9 | 1,386664733 | 0,011378271 | 0,999933142 |
| ENSMUSG00000029417 | Cxcl9 | -2,622227912 | 0,011464135 | 0,999933142 |
| ENSMUSG00000060802 | B2m | -0,439894062 | 0,011467825 | 0,999933142 |
| ENSMUSG00000065110 | Snord61 | -1,508511894 | 0,011541721 | 0,999933142 |
| ENSMUSG00000003814 | Calr | -0,351468271 | 0,011726384 | 0,999933142 |
| ENSMUSG00000073491 | Ifi213 | -1,670902294 | 0,011853224 | 0,999933142 |
| ENSMUSG00000037071 | Scd1 | -0,836474303 | 0,011862882 | 0,999933142 |
| ENSMUSG00000065878 | Snord34 | -1,841268373 | 0,012094724 | 0,999933142 |
| ENSMUSG00000095026 | Gm3336 | 2,970095024 | 0,012285007 | 0,999933142 |
| ENSMUSG00000086801 | Gm15943 | -2,391399278 | 0,012517969 | 0,999933142 |
| ENSMUSG00000044471 | Lncpint | 0,353608766 | 0,01263173 | 0,999933142 |
| ENSMUSG00000059277 | R74862 | -0,526094758 | 0,013003949 | 0,999933142 |
| ENSMUSG00000057322 | Rpl38 | -0,472049716 | 0,013215852 | 0,999933142 |
| ENSMUSG00000007892 | Rplp1 | -0,412140361 | 0,013261228 | 0,999933142 |
| ENSMUSG00000025232 | Hexa | -0,360293973 | 0,013336703 | 0,999933142 |
| ENSMUSG00000035678 | Tnfsf9 | 1,347465342 | 0,013386887 | 0,999933142 |
| ENSMUSG00000020641 | Rsad2 | -1,796753761 | 0,013413401 | 0,999933142 |
| ENSMUSG00000065738 | Gm24494 | -1,317286385 | 0,013625735 | 0,999933142 |
| ENSMUSG00000000346 | Dazap2 | -0,329112263 | 0,013716484 | 0,999933142 |
| ENSMUSG00000064254 | Ethe1 | -1,566833332 | 0,01377199 | 0,999933142 |
| ENSMUSG00000071753 | C230004F18Rik | 0,629087422 | 0,013789127 | 0,999933142 |
| ENSMUSG00000015672 | Mrpl32 | -0,864258988 | 0,01382242 | 0,999933142 |
| ENSMUSG00000020681 | Ace | 1,810334521 | 0,014008748 | 0,999933142 |
| ENSMUSG00000025508 | Rplp2 | -0,393750045 | 0,014104503 | 0,999933142 |
| ENSMUSG00000038811 | Gngt2 | -0,452141725 | 0,014231993 | 0,999933142 |
| ENSMUSG00000112637 | Gm48225 | 0,999563728 | 0,014373479 | 0,999933142 |
| ENSMUSG00000048012 | Zfp473 | -4,952816695 | 0,014476934 | 0,999933142 |
| ENSMUSG00000083863 | Gm13341 | -1,442759644 | 0,01477435 | 0,999933142 |
| ENSMUSG00000027346 | Gpcpd1 | 0,374707484 | 0,014927458 | 0,999933142 |
| ENSMUSG00000090207 | 4930524O07Rik | -1,869329395 | 0,01526339 | 0,999933142 |
| ENSMUSG00000065191 | Gm23458 | 4,458995362 | 0,015332402 | 0,999933142 |
| ENSMUSG00000106251 | Gm42658 | 0,837338386 | 0,015867277 | 0,999933142 |
| ENSMUSG00000060126 | Tpt1 | -0,292178332 | 0,016015405 | 0,999933142 |
| ENSMUSG00000025212 | Sfxn3 | -0,628506103 | 0,016153363 | 0,999933142 |
| ENSMUSG00002075283 |  | 2,106543834 | 0,016299928 | 0,999933142 |
| ENSMUSG00000048076 | Arf1 | -0,321611606 | 0,016409435 | 0,999933142 |
| ENSMUSG00000056076 | Eif3b | -0,424497685 | 0,016453633 | 0,999933142 |
| ENSMUSG00000038274 | Fau | -0,423288692 | 0,016498113 | 0,999933142 |
| ENSMUSG00000100862 | Gm10925 | -0,559421396 | 0,016519001 | 0,999933142 |
| ENSMUSG00000121280 |  | 0,529452606 | 0,01658469 | 0,999933142 |
| ENSMUSG00000075010 | AW112010 | -1,231215376 | 0,01679211 | 0,999933142 |
| ENSMUSG00000109429 | Gm45223 | 0,322657695 | 0,016998234 | 0,999933142 |
| ENSMUSG00000030122 | Ptms | -0,312888492 | 0,017149738 | 0,999933142 |
| ENSMUSG00000028957 | Per3 | 0,584826684 | 0,017356332 | 0,999933142 |
| ENSMUSG00000064039 | Ccr1l1 | 0,862355068 | 0,017406804 | 0,999933142 |
| ENSMUSG00000022048 | Dpysl2 | -0,288563649 | 0,017691188 | 0,999933142 |
| ENSMUSG00000052387 | Trpm3 | 0,981330237 | 0,017941219 | 0,999933142 |
| ENSMUSG00000026222 | Sp100 | -0,657398468 | 0,018026746 | 0,999933142 |
| ENSMUSG00000071415 | Rpl23 | -0,361795356 | 0,018177281 | 0,999933142 |
| ENSMUSG00000120081 |  | 0,617201322 | 0,018306244 | 0,999933142 |
| ENSMUSG00000056116 | H2-T22 | -0,815540836 | 0,018385426 | 0,999933142 |
| ENSMUSG00000061167 | Rpl15-ps3 | -4,789868351 | 0,018438546 | 0,999933142 |
| ENSMUSG00000105771 | 2900064K03Rik | 3,798176711 | 0,018520532 | 0,999933142 |
| ENSMUSG00000029810 | Tmem176b | -0,346376242 | 0,018524545 | 0,999933142 |
| ENSMUSG00000030691 | Fchsd2 | 0,275504276 | 0,018542683 | 0,999933142 |
| ENSMUSG00000038642 | Ctss | -0,312533958 | 0,018692406 | 0,999933142 |
| ENSMUSG00000006315 | Tmem147 | -0,711091071 | 0,018706931 | 0,999933142 |
| ENSMUSG00000018293 | Pfn1 | -0,455021656 | 0,018805502 | 0,999933142 |
| ENSMUSG00002075094 |  | -2,063751667 | 0,018868864 | 0,999933142 |
| ENSMUSG00000058799 | Nap1l1 | -0,309204824 | 0,01891574 | 0,999933142 |
| ENSMUSG00000009927 | Rps25 | -0,340827157 | 0,019243863 | 0,999933142 |
| ENSMUSG00000055850 | Rnf181 | -0,652868778 | 0,019352876 | 0,999933142 |
| ENSMUSG00000107624 | Gm44005 | 1,038857392 | 0,019390845 | 0,999933142 |
| ENSMUSG00000108500 | Gm45033 | 1,174441741 | 0,019749787 | 0,999933142 |
| ENSMUSG00000018593 | Sparc | -0,287009656 | 0,019803518 | 0,999933142 |
| ENSMUSG00000119403 |  | -1,577041325 | 0,019977889 | 0,999933142 |
| ENSMUSG00000028132 | Tmem56 | -1,931089163 | 0,020103891 | 0,999933142 |
| ENSMUSG00000041697 | Cox6a1 | -0,512243175 | 0,020489358 | 0,999933142 |
| ENSMUSG00000052707 | Tnrc6a | 0,341105099 | 0,020556582 | 0,999933142 |
| ENSMUSG00000028416 | Bag1 | -0,380684706 | 0,020583782 | 0,999933142 |
| ENSMUSG00000083992 | Gm11478 | -0,968700624 | 0,020607292 | 0,999933142 |
| ENSMUSG00000020460 | Rps27a | -0,298316814 | 0,020743151 | 0,999933142 |
| ENSMUSG00000003435 | Supt5 | -0,361937559 | 0,021145802 | 0,999933142 |
| ENSMUSG00000120777 | Gm56626 | 1,342645451 | 0,021299553 | 0,999933142 |
| ENSMUSG00000111131 | 9930024M15Rik | 1,164107633 | 0,021530825 | 0,999933142 |
| ENSMUSG00000026641 | Usf1 | -0,591448961 | 0,021578871 | 0,999933142 |
| ENSMUSG00000086496 | Gm14204 | 4,323393431 | 0,021608697 | 0,999933142 |
| ENSMUSG00000073434 | Wdr90 | 1,226186701 | 0,021621554 | 0,999933142 |
| ENSMUSG00000030579 | Tyrobp | -0,395706143 | 0,021878122 | 0,999933142 |
| ENSMUSG00000098720 | Gm27239 | 4,923031951 | 0,021929994 | 0,999933142 |
| ENSMUSG00000027447 | Cst3 | -0,343563021 | 0,022263974 | 0,999933142 |
| ENSMUSG00000108701 | Gm44616 | -0,571810635 | 0,022547393 | 0,999933142 |
| ENSMUSG00000120205 |  | -1,562973847 | 0,022590059 | 0,999933142 |
| ENSMUSG00000065208 | Gm24616 | -1,596025444 | 0,022652675 | 0,999933142 |
| ENSMUSG00000025374 | Nabp2 | -0,764044949 | 0,022801989 | 0,999933142 |
| ENSMUSG00000022425 | Enpp2 | 0,628618968 | 0,02281407 | 0,999933142 |
| ENSMUSG00000110914 | Gm48611 | 0,481246534 | 0,022814137 | 0,999933142 |
| ENSMUSG00002075501 |  | -0,973170873 | 0,022846761 | 0,999933142 |
| ENSMUSG00000087185 | Gm13872 | -1,838176299 | 0,022912232 | 0,999933142 |
| ENSMUSG00000005142 | Man2b1 | -0,315332602 | 0,023140498 | 0,999933142 |
| ENSMUSG00000025270 | Alas2 | -4,71516973 | 0,023228116 | 0,999933142 |
| ENSMUSG00000089093 | Snord11 | -1,763663155 | 0,02334999 | 0,999933142 |
| ENSMUSG00000119142 |  | -0,720963701 | 0,023413209 | 0,999933142 |
| ENSMUSG00000036779 | Papd5 | 0,409171378 | 0,023427165 | 0,999933142 |
| ENSMUSG00000034656 | Cacna1a | 0,526695541 | 0,023457454 | 0,999933142 |
| ENSMUSG00000028134 | Ptbp2 | 0,380660155 | 0,023704083 | 0,999933142 |
| ENSMUSG00000063457 | Rps15 | -0,380832922 | 0,023734951 | 0,999933142 |
| ENSMUSG00000023473 | Celsr3 | 1,325198535 | 0,023745754 | 0,999933142 |
| ENSMUSG00000077222 | Gm22270 | -0,840390701 | 0,024058012 | 0,999933142 |
| ENSMUSG00000104591 | Gm43145 | 0,593608838 | 0,024131371 | 0,999933142 |
| ENSMUSG00000026458 | Ppfia4 | -0,285520152 | 0,024175196 | 0,999933142 |
| ENSMUSG00000120800 |  | 0,508634905 | 0,024195115 | 0,999933142 |
| ENSMUSG00000039057 | Myo16 | 2,395987404 | 0,024230878 | 0,999933142 |
| ENSMUSG00000089774 | Slc5a3 | 0,465904242 | 0,024233706 | 0,999933142 |
| ENSMUSG00000098230 | 1700095B10Rik | -3,995149903 | 0,024280133 | 0,999933142 |
| ENSMUSG00000074896 | Ifit3 | -1,162072951 | 0,024323828 | 0,999933142 |
| ENSMUSG00000007812 | Zfp655 | 0,405969468 | 0,024450369 | 0,999933142 |
| ENSMUSG00000119892 |  | 0,819050964 | 0,024510622 | 0,999933142 |
| ENSMUSG00000044667 | Plppr4 | -1,073506697 | 0,024688848 | 0,999933142 |
| ENSMUSG00002075637 |  | -1,985309005 | 0,024858895 | 0,999933142 |
| ENSMUSG00000029343 | Crybb1 | -0,50079309 | 0,025132784 | 0,999933142 |
| ENSMUSG00000015312 | Gadd45b | 0,987606561 | 0,025211299 | 0,999933142 |
| ENSMUSG00000031428 | Zcchc18 | 1,693035636 | 0,025751814 | 0,999933142 |
| ENSMUSG00000032911 | Cspg4 | 1,500831205 | 0,025793843 | 0,999933142 |
| ENSMUSG00000042708 | Shcbp1l | -3,228357081 | 0,026087206 | 0,999933142 |
| ENSMUSG00000064373 | Selenop | -0,258674485 | 0,026099721 | 0,999933142 |
| ENSMUSG00000103948 | 4930594C11Rik | 0,520194129 | 0,026263561 | 0,999933142 |
| ENSMUSG00000049775 | Tmsb4x | -0,291896012 | 0,026317547 | 0,999933142 |
| ENSMUSG00000026238 | Ptma | -0,265186012 | 0,026323461 | 0,999933142 |
| ENSMUSG00000045328 | Cenpe | -1,971100486 | 0,026459042 | 0,999933142 |
| ENSMUSG00000119286 |  | -1,228400703 | 0,026489988 | 0,999933142 |
| ENSMUSG00000106001 | Gm42826 | -1,56128224 | 0,026541035 | 0,999933142 |
| ENSMUSG00000037868 | Egr2 | 0,560743944 | 0,02664593 | 0,999933142 |
| ENSMUSG00000029413 | Naaa | -0,417071919 | 0,026686654 | 0,999933142 |
| ENSMUSG00000011837 | Snapc2 | -0,928174573 | 0,026945528 | 0,999933142 |
| ENSMUSG00000049517 | Rps23 | -0,304250541 | 0,026964911 | 0,999933142 |
| ENSMUSG00000111340 | Gm47171 | 4,882452112 | 0,026980856 | 0,999933142 |
| ENSMUSG00000028639 | Ybx1 | -0,320743445 | 0,027002721 | 0,999933142 |
| ENSMUSG00000106211 | Gm42842 | 1,036983384 | 0,027458494 | 0,999933142 |
| ENSMUSG00000084950 | Gm5577 | -1,104116502 | 0,027462915 | 0,999933142 |
| ENSMUSG00000032551 | 1110059G10Rik | -0,627677213 | 0,027582583 | 0,999933142 |
| ENSMUSG00000109585 | Gm45358 | 0,995999427 | 0,02773375 | 0,999933142 |
| ENSMUSG00000080058 | Gm11175 | -0,491067732 | 0,028025193 | 0,999933142 |
| ENSMUSG00000115624 | AC154640.4 | 0,549002266 | 0,028253398 | 0,999933142 |
| ENSMUSG00000022340 | Sybu | 2,100928093 | 0,02858355 | 0,999933142 |
| ENSMUSG00000037646 | Vps13b | 0,281858244 | 0,028588226 | 0,999933142 |
| ENSMUSG00000109709 | Gm45290 | -3,203665386 | 0,028666232 | 0,999933142 |
| ENSMUSG00000115276 | AC149588.1 | 0,550960409 | 0,028843047 | 0,999933142 |
| ENSMUSG00000120982 |  | -3,144126525 | 0,028877534 | 0,999933142 |
| ENSMUSG00000119765 |  | -1,04217251 | 0,029006701 | 0,999933142 |
| ENSMUSG00000032294 | Pkm | -0,296499526 | 0,029015829 | 0,999933142 |
| ENSMUSG00000087222 | E030042O20Rik | 0,86862214 | 0,029405516 | 0,999933142 |
| ENSMUSG00000021520 | Uqcrb | -0,559560773 | 0,029778299 | 0,999933142 |
| ENSMUSG00000026153 | Fam135a | 0,45405306 | 0,029808405 | 0,999933142 |
| ENSMUSG00000037206 | Islr | 4,26059084 | 0,029863984 | 0,999933142 |
| ENSMUSG00000062353 | Gm15772 | -0,608035863 | 0,029937912 | 0,999933142 |
| ENSMUSG00000062328 | Rpl17 | -0,365102349 | 0,02995787 | 0,999933142 |
| ENSMUSG00000101111 | Gm28437 | -0,603154924 | 0,029982739 | 0,999933142 |
| ENSMUSG00000023992 | Trem2 | -0,308933642 | 0,030010265 | 0,999933142 |
| ENSMUSG00000056501 | Cebpb | 0,472126837 | 0,0300264 | 0,999933142 |
| ENSMUSG00000104145 | D130019J16Rik | 0,617298794 | 0,030066608 | 0,999933142 |
| ENSMUSG00000031125 | 3830403N18Rik | -0,967052246 | 0,030226626 | 0,999933142 |
| ENSMUSG00000026223 | Itm2c | -0,315011918 | 0,03028819 | 0,999933142 |
| ENSMUSG00000060988 | Galnt13 | 3,222384408 | 0,030500141 | 0,999933142 |
| ENSMUSG00000037486 | Asxl2 | 0,306987937 | 0,030577223 | 0,999933142 |
| ENSMUSG00000024997 | Prdx3 | -0,44476747 | 0,030610306 | 0,999933142 |
| ENSMUSG00000097559 | D430018E03Rik | 1,891472818 | 0,030663192 | 0,999933142 |
| ENSMUSG00000107000 | Gm43481 | 0,529241319 | 0,030752699 | 0,999933142 |
| ENSMUSG00000118521 | Gm52948 | -2,515871441 | 0,030889439 | 0,999933142 |
| ENSMUSG00000039001 | Rps21 | -0,349415365 | 0,03139463 | 0,999933142 |
| ENSMUSG00000031791 | Tmem38a | 1,511581878 | 0,031455002 | 0,999933142 |
| ENSMUSG00000029621 | Arpc1a | -0,463657809 | 0,03158475 | 0,999933142 |
| ENSMUSG00000000982 | Ccl3 | 0,394035755 | 0,031847828 | 0,999933142 |
| ENSMUSG00000086717 | Gm15655 | -0,932654851 | 0,031853454 | 0,999933142 |
| ENSMUSG00000119920 |  | -1,366147978 | 0,031901354 | 0,999933142 |
| ENSMUSG00000110320 | Gm31152 | 4,861303763 | 0,032044625 | 0,999933142 |
| ENSMUSG00000025393 | Atp5b | -0,29298958 | 0,032157611 | 0,999933142 |
| ENSMUSG00000079017 | Ifi27l2a | -1,448955847 | 0,032310649 | 0,999933142 |
| ENSMUSG00000065728 | Gm26175 | -0,906348408 | 0,032395647 | 0,999933142 |
| ENSMUSG00000047496 | Rnf152 | 2,54531455 | 0,032452846 | 0,999933142 |
| ENSMUSG00000065258 | Gm23969 | -1,516041219 | 0,032659283 | 0,999933142 |
| ENSMUSG00000090389 | Cdv3-ps | -4,51877711 | 0,032689639 | 0,999933142 |
| ENSMUSG00000081255 | Gm5380 | -4,590658483 | 0,032748541 | 0,999933142 |
| ENSMUSG00000033730 | Egr3 | 0,447285527 | 0,03277437 | 0,999933142 |
| ENSMUSG00000093413 | Snora15 | -0,724801534 | 0,032802063 | 0,999933142 |
| ENSMUSG00000090086 | AI480526 | 0,553299658 | 0,032945859 | 0,999933142 |
| ENSMUSG00000055102 | Zfp819 | 4,771523126 | 0,032992813 | 0,999933142 |
| ENSMUSG00000090142 | Gm15795 | -4,525515824 | 0,033023567 | 0,999933142 |
| ENSMUSG00000117313 | Gm49838 | -1,049728362 | 0,033566616 | 0,999933142 |
| ENSMUSG00002075706 |  | -4,582182451 | 0,033584843 | 0,999933142 |
| ENSMUSG00000103804 | Gm37062 | 1,513654551 | 0,033594772 | 0,999933142 |
| ENSMUSG00000059355 | Wdr83os | -0,494759338 | 0,033631639 | 0,999933142 |
| ENSMUSG00000103214 | Gm38286 | 3,658266522 | 0,033757508 | 0,999933142 |
| ENSMUSG00000027667 | Zfp639 | 0,534855855 | 0,034212368 | 0,999933142 |
| ENSMUSG00000086368 | Gm13830 | 3,566762054 | 0,034276896 | 0,999933142 |
| ENSMUSG00000038764 | Ptpn3 | 2,512546083 | 0,03429662 | 0,999933142 |
| ENSMUSG00000025494 | Sigirr | 4,133514811 | 0,034472512 | 0,999933142 |
| ENSMUSG00000111686 | Rab7-ps1 | -3,112208602 | 0,034717195 | 0,999933142 |
| ENSMUSG00000064330 | Pde6h | -0,845395311 | 0,035004361 | 0,999933142 |
| ENSMUSG00000046714 | Foxc2 | 4,243825877 | 0,035094356 | 0,999933142 |
| ENSMUSG00000030432 | Rpl28 | -0,378648529 | 0,035147796 | 0,999933142 |
| ENSMUSG00000039826 | Trub2 | 0,65249094 | 0,035185482 | 0,999933142 |
| ENSMUSG00000029649 | Pomp | -0,394766139 | 0,035232195 | 0,999933142 |
| ENSMUSG00000001270 | Ckb | -0,290034711 | 0,03527103 | 0,999933142 |
| ENSMUSG00000115210 | AC120375.1 | 1,823884472 | 0,035293909 | 0,999933142 |
| ENSMUSG00000068314 | Gm6899 | 3,5334031 | 0,035296941 | 0,999933142 |
| ENSMUSG00000024308 | Tapbp | -0,351744378 | 0,035329619 | 0,999933142 |
| ENSMUSG00000023092 | Fhl1 | 2,0794893 | 0,035426311 | 0,999933142 |
| ENSMUSG00000028923 | Necap2 | -0,390742602 | 0,03558136 | 0,999933142 |
| ENSMUSG00000057378 | Ryr3 | 1,143458506 | 0,035621645 | 0,999933142 |
| ENSMUSG00000035835 | Plppr3 | -0,981256916 | 0,035641097 | 0,999933142 |
| ENSMUSG00000075700 | Selenot | -0,266997612 | 0,035698374 | 0,999933142 |
| ENSMUSG00000043263 | Ifi209 | -0,802464064 | 0,035774027 | 0,999933142 |
| ENSMUSG00000028698 | Pik3r3 | 0,981876163 | 0,036284195 | 0,999933142 |
| ENSMUSG00000109498 | Gm45222 | 0,268954181 | 0,036360011 | 0,999933142 |
| ENSMUSG00000052760 | A630001G21Rik | 0,443052505 | 0,036436514 | 0,999933142 |
| ENSMUSG00000025795 | Rassf3 | -0,394643861 | 0,036488871 | 0,999933142 |
| ENSMUSG00000056313 | Tcim | -0,766031551 | 0,036530547 | 0,999933142 |
| ENSMUSG00000019437 | Tlcd1 | -0,497304972 | 0,036677076 | 0,999933142 |
| ENSMUSG00000121063 | Gm56614 | 0,506183727 | 0,036698373 | 0,999933142 |
| ENSMUSG00000028876 | Epha10 | 2,178968915 | 0,036714007 | 0,999933142 |
| ENSMUSG00000101731 | 3830432H09Rik | 4,749108799 | 0,036725536 | 0,999933142 |
| ENSMUSG00000001123 | Lgals9 | -0,354070681 | 0,037360217 | 0,999933142 |
| ENSMUSG00000101599 | Gm20342 | 0,567571413 | 0,037697728 | 0,999933142 |
| ENSMUSG00000055401 | Fbxo6 | -0,775102291 | 0,037748061 | 0,999933142 |
| ENSMUSG00000021939 | Ctsb | -0,355203968 | 0,038141072 | 0,999933142 |
| ENSMUSG00000108543 | Gm44735 | 1,406137646 | 0,038171253 | 0,999933142 |
| ENSMUSG00000056201 | Cfl1 | -0,316613655 | 0,038409106 | 0,999933142 |
| ENSMUSG00000021866 | Anxa11 | -0,651443932 | 0,038612062 | 0,999933142 |
| ENSMUSG00000060143 | Gm10076 | -0,583075682 | 0,038695646 | 0,999933142 |
| ENSMUSG00000000420 | Galnt1 | -0,276666398 | 0,038735191 | 0,999933142 |
| ENSMUSG00000045822 | Zswim3 | -1,176502053 | 0,038780736 | 0,999933142 |
| ENSMUSG00000020572 | Nampt | -0,404022538 | 0,038910477 | 0,999933142 |
| ENSMUSG00000064540 | Snord42a | -3,9032906 | 0,039243222 | 0,999933142 |
| ENSMUSG00000028729 | Ebna1bp2 | -0,629575474 | 0,039380668 | 0,999933142 |
| ENSMUSG00000070003 | Ssbp4 | -0,462573936 | 0,039406779 | 0,999933142 |
| ENSMUSG00000032562 | Gnai2 | -0,283857786 | 0,039416296 | 0,999933142 |
| ENSMUSG00000021242 | Npc2 | -0,352859439 | 0,03950032 | 0,999933142 |
| ENSMUSG00000038429 | Usp5 | -0,408608479 | 0,039754919 | 0,999933142 |
| ENSMUSG00000030220 | Arhgdib | -0,318158228 | 0,039767812 | 0,999933142 |
| ENSMUSG00000021936 | Mapk8 | 0,430197945 | 0,039988255 | 0,999933142 |
| ENSMUSG00000117814 | Gm50431 | 1,420013362 | 0,040299789 | 0,999933142 |
| ENSMUSG00000063317 | Usp31 | 0,441468274 | 0,040463621 | 0,999933142 |
| ENSMUSG00000020451 | Limk2 | 0,623791304 | 0,040741036 | 0,999933142 |
| ENSMUSG00000103685 | Gm37074 | 2,023179745 | 0,040790204 | 0,999933142 |
| ENSMUSG00000057666 | Gapdh | -0,296697887 | 0,040834113 | 0,999933142 |
| ENSMUSG00000030738 | Eif3c | -0,295408312 | 0,040906721 | 0,999933142 |
| ENSMUSG00000022972 | 1110004E09Rik | -1,483488941 | 0,041165755 | 0,999933142 |
| ENSMUSG00000038650 | Rnh1 | -0,430680324 | 0,041170428 | 0,999933142 |
| ENSMUSG00000045215 | Asxl3 | -3,050355095 | 0,041227438 | 0,999933142 |
| ENSMUSG00000033220 | Rac2 | -0,390195312 | 0,0413165 | 0,999933142 |
| ENSMUSG00000097004 | 4731419I09Rik | -0,694330274 | 0,041393483 | 0,999933142 |
| ENSMUSG00000052310 | Slc39a1 | -0,364993208 | 0,041479572 | 0,999933142 |
| ENSMUSG00000092482 | Gm20531 | -0,93072159 | 0,041531265 | 0,999933142 |
| ENSMUSG00000024397 | Aif1 | -0,29341765 | 0,04154441 | 0,999933142 |
| ENSMUSG00000023010 | Tmbim6 | -0,27973803 | 0,041703501 | 0,999933142 |
| ENSMUSG00000020372 | Rack1 | -0,278060161 | 0,04198929 | 0,999933142 |
| ENSMUSG00000049299 | Trappc1 | -0,577770077 | 0,042122848 | 0,999933142 |
| ENSMUSG00000045968 | Teddm2 | -0,721063957 | 0,042175731 | 0,999933142 |
| ENSMUSG00000073102 | Drc1 | 4,062449163 | 0,042226434 | 0,999933142 |
| ENSMUSG00000026112 | Coa5 | -0,30397341 | 0,042304622 | 0,999933142 |
| ENSMUSG00000107054 | C730045M19Rik | 0,860707928 | 0,042452841 | 0,999933142 |
| ENSMUSG00000030695 | Aldoa | -0,334445211 | 0,042512551 | 0,999933142 |
| ENSMUSG00000029815 | Malsu1 | 0,598331322 | 0,042651829 | 0,999933142 |
| ENSMUSG00000063882 | Uqcrh | -0,416118723 | 0,042669929 | 0,999933142 |
| ENSMUSG00000110282 | B930086L07Rik | 0,548445836 | 0,042774963 | 0,999933142 |
| ENSMUSG00000117943 |  | 2,680924538 | 0,042901583 | 0,999933142 |
| ENSMUSG00000085448 | Gm13963 | 4,72600443 | 0,042939458 | 0,999933142 |
| ENSMUSG00000106048 | Gm42444 | 4,72600443 | 0,042939458 | 0,999933142 |
| ENSMUSG00000107643 | Gm43885 | 4,72600443 | 0,042939458 | 0,999933142 |
| ENSMUSG00000004296 | Il12b | 4,72600443 | 0,042939458 | 0,999933142 |
| ENSMUSG00000092300 | Cdk3-ps | 4,72600443 | 0,042939458 | 0,999933142 |
| ENSMUSG00000105561 | Gm43462 | 0,456826228 | 0,043274511 | 0,999933142 |
| ENSMUSG00000087367 | Gm15491 | -1,994262504 | 0,043286109 | 0,999933142 |
| ENSMUSG00000029580 | Actb | -0,26272833 | 0,043717447 | 0,999933142 |
| ENSMUSG00000021022 | Ppp2r3c | -0,470389294 | 0,043737082 | 0,999933142 |
| ENSMUSG00000028029 | Aimp1 | -0,488526708 | 0,043900167 | 0,999933142 |
| ENSMUSG00000032549 | Rab6b | -0,300462916 | 0,043929885 | 0,999933142 |
| ENSMUSG00000014592 | Camta1 | 0,52739879 | 0,044011979 | 0,999933142 |
| ENSMUSG00000021196 | Pfkp | 1,008524118 | 0,044075926 | 0,999933142 |
| ENSMUSG00000001627 | Ifrd1 | 0,372028076 | 0,04461454 | 0,999933142 |
| ENSMUSG00000006740 | Kif5b | -0,249957426 | 0,044665408 | 0,999933142 |
| ENSMUSG00000103216 | Gm37248 | 2,633210512 | 0,044766655 | 0,999933142 |
| ENSMUSG00000120618 | Gm9360 | 4,037735553 | 0,04492455 | 0,999933142 |
| ENSMUSG00000018585 | Atox1 | -0,586416362 | 0,044986115 | 0,999933142 |
| ENSMUSG00000103103 | 4833445I07Rik | 0,850359942 | 0,045010359 | 0,999933142 |
| ENSMUSG00000042942 | Greb1l | 2,480256605 | 0,045026977 | 0,999933142 |
| ENSMUSG00000061062 | Gm10093 | -3,724611898 | 0,045305617 | 0,999933142 |
| ENSMUSG00000073557 | Ppp1r12b | 0,425754603 | 0,045337474 | 0,999933142 |
| ENSMUSG00000113386 | Gm47357 | 1,029931822 | 0,045420066 | 0,999933142 |
| ENSMUSG00000103309 | BC037039 | -3,729345574 | 0,045434404 | 0,999933142 |
| ENSMUSG00000028394 | Pole3 | -0,655792104 | 0,045836128 | 0,999933142 |
| ENSMUSG00000040710 | St8sia4 | 0,410105432 | 0,046025304 | 0,999933142 |
| ENSMUSG00000062461 | Gm5453 | -2,369777256 | 0,046226837 | 0,999933142 |
| ENSMUSG00002075524 |  | -0,978157594 | 0,046267751 | 0,999933142 |
| ENSMUSG00000033186 | Mzt1 | -0,484995579 | 0,046276511 | 0,999933142 |
| ENSMUSG00000117839 | Gm50251 | -1,47673433 | 0,046491235 | 0,999933142 |
| ENSMUSG00000041560 | Nop53 | -0,346271919 | 0,046766953 | 0,999933142 |
| ENSMUSG00000045136 | Tubb2b | -0,48187741 | 0,046836549 | 0,999933142 |
| ENSMUSG00000044712 | Slc38a6 | 0,51284398 | 0,047191781 | 0,999933142 |
| ENSMUSG00000102733 | Gm38262 | 0,498174578 | 0,047195366 | 0,999933142 |
| ENSMUSG00000065563 | Mir103-2 | 3,06537261 | 0,04723114 | 0,999933142 |
| ENSMUSG00000025132 | Arhgdia | -0,313715777 | 0,047242327 | 0,999933142 |
| ENSMUSG00000023951 | Vegfa | 0,893324618 | 0,047553777 | 0,999933142 |
| ENSMUSG00000002871 | Tpra1 | 0,507807363 | 0,047862353 | 0,999933142 |
| ENSMUSG00000048058 | Ldlrad3 | -1,526184112 | 0,047894095 | 0,999933142 |
| ENSMUSG00000022948 | Setd4 | 1,188479 | 0,047934549 | 0,999933142 |
| ENSMUSG00000035042 | Ccl5 | -1,87022847 | 0,048250502 | 0,999933142 |
| ENSMUSG00000029247 | Paics | -0,345110226 | 0,048292002 | 0,999933142 |
| ENSMUSG00000091649 | Phf11b | -0,643391191 | 0,048306962 | 0,999933142 |
| ENSMUSG00000020167 | Tcf3 | -0,459770993 | 0,048422456 | 0,999933142 |
| ENSMUSG00000043811 | Rtn4r | 3,124333644 | 0,048461342 | 0,999933142 |
| ENSMUSG00000034424 | Gcsh | -0,713810569 | 0,048496775 | 0,999933142 |
| ENSMUSG00000001034 | Mapk7 | -0,593380092 | 0,048538674 | 0,999933142 |
| ENSMUSG00000110424 | 1700012D14Rik | 1,013702743 | 0,048658824 | 0,999933142 |
| ENSMUSG00000110187 | Gm45496 | 3,521779237 | 0,048818735 | 0,999933142 |
| ENSMUSG00000065057 | Gm25703 | -1,298273791 | 0,048819984 | 0,999933142 |
| ENSMUSG00000074923 | Pak6 | -2,148206562 | 0,049107824 | 0,999933142 |
| ENSMUSG00000106553 | 6720482G16Rik | 2,011990652 | 0,049133864 | 0,999933142 |
| ENSMUSG00000119584 | GRCm39 | -0,716474456 | 0,049200674 | 0,999933142 |
| ENSMUSG00000030281 | Il17rc | -4,524097297 | 0,049347474 | 0,999933142 |
| ENSMUSG00000096795 | Zfp433 | 1,636767937 | 0,049423904 | 0,999933142 |
| ENSMUSG00000056476 | Med12l | 0,345618811 | 0,049492438 | 0,999933142 |
| ENSMUSG00000031391 | L1cam | 1,412431103 | 0,049506803 | 0,999933142 |
| ENSMUSG00000000740 | Rpl13 | -0,326406484 | 0,049603646 | 0,999933142 |
| ENSMUSG00000042097 | Zfp239 | -2,906978922 | 0,049737277 | 0,999933142 |
| ENSMUSG00000025470 | Zfp511 | -0,990956245 | 0,049813554 | 0,999933142 |
| ENSMUSG00000065701 | Rny1 | -1,101793448 | 0,049857783 | 0,999933142 |
| ENSMUSG00000103251 | Gm37519 | 0,943521959 | 0,049861714 | 0,999933142 |

**Supplemental Table 3. Significant enriched GO Biological processes in hippocampal microglia**

| **Category** | **ID** | **Name** | **q-value FDR B&H** | **q-value FDR B&Y** |
| --- | --- | --- | --- | --- |
| GO: Biological Process | GO:0002181 | cytoplasmic translation | 1.56E-12 | 1.39E-11 |
| GO: Biological Process | GO:0006412 | translation | 1.63E-08 | 1.46E-07 |
| GO: Biological Process | GO:0140236 | translation at presynapse | 3.07E-06 | 2.74E-05 |
| GO: Biological Process | GO:0140241 | translation at synapse | 3.07E-06 | 2.74E-05 |
| GO: Biological Process | GO:0140242 | translation at postsynapse | 3.07E-06 | 2.74E-05 |
| GO: Biological Process | GO:0042981 | regulation of apoptotic process | 5.68E-04 | 5.08E-03 |
| GO: Biological Process | GO:0042255 | ribosome assembly | 9.24E-04 | 8.27E-03 |
| GO: Biological Process | GO:0043067 | regulation of programmed cell death | 9.24E-04 | 8.27E-03 |
| GO: Biological Process | GO:0001774 | microglial cell activation | 1.84E-03 | 1.65E-02 |
| GO: Biological Process | GO:0150062 | complement-mediated synapse pruning | 2.28E-03 | 2.04E-02 |
| GO: Biological Process | GO:0002269 | leukocyte activation involved in inflammatory response | 2.36E-03 | 2.11E-02 |
| GO: Biological Process | GO:0045672 | positive regulation of osteoclast differentiation | 3.05E-03 | 2.73E-02 |
| GO: Biological Process | GO:0051235 | maintenance of location | 3.07E-03 | 2.75E-02 |
| GO: Biological Process | GO:0061900 | glial cell activation | 3.76E-03 | 3.36E-02 |
| GO: Biological Process | GO:1903708 | positive regulation of hemopoiesis | 4.37E-03 | 3.91E-02 |
| GO: Biological Process | GO:1902107 | positive regulation of leukocyte differentiation | 4.37E-03 | 3.91E-02 |
| GO: Biological Process | GO:0010720 | positive regulation of cell development | 4.37E-03 | 3.91E-02 |
| GO: Biological Process | GO:0006935 | chemotaxis | 4.44E-03 | 3.98E-02 |
| GO: Biological Process | GO:0042330 | taxis | 4.44E-03 | 3.98E-02 |
| GO: Biological Process | GO:0048878 | chemical homeostasis | 4.44E-03 | 3.98E-02 |
| GO: Biological Process | GO:0043066 | negative regulation of apoptotic process | 4.58E-03 | 4.10E-02 |
| GO: Biological Process | GO:0002397 | MHC class I protein complex assembly | 4.76E-03 | 4.26E-02 |
| GO: Biological Process | GO:0002502 | peptide antigen assembly with MHC class I protein complex | 4.76E-03 | 4.26E-02 |
| GO: Biological Process | GO:0071826 | protein-RNA complex organization | 4.76E-03 | 4.26E-02 |
| GO: Biological Process | GO:0065003 | protein-containing complex assembly | 4.76E-03 | 4.26E-02 |
| GO: Biological Process | GO:0019080 | viral gene expression | 4.76E-03 | 4.26E-02 |
| GO: Biological Process | GO:0040011 | locomotion | 4.83E-03 | 4.32E-02 |
| GO: Biological Process | GO:0060284 | regulation of cell development | 4.83E-03 | 4.32E-02 |
| GO: Biological Process | GO:0051651 | maintenance of location in cell | 4.83E-03 | 4.32E-02 |
| GO: Biological Process | GO:0019725 | cellular homeostasis | 4.83E-03 | 4.32E-02 |
| GO: Biological Process | GO:0055082 | intracellular chemical homeostasis | 4.83E-03 | 4.32E-02 |
| GO: Biological Process | GO:0002763 | positive regulation of myeloid leukocyte differentiation | 5.31E-03 | 4.75E-02 |
| GO: Biological Process | GO:0043069 | negative regulation of programmed cell death | 5.31E-03 | 4.75E-02 |
| GO: Biological Process | GO:0019083 | viral transcription | 5.42E-03 | 4.85E-02 |
| GO: Biological Process | GO:0098883 | synapse pruning | 6.09E-03 | 5.45E-02 |
| GO: Biological Process | GO:0060326 | cell chemotaxis | 6.70E-03 | 5.99E-02 |
| GO: Biological Process | GO:2000147 | positive regulation of cell motility | 6.85E-03 | 6.13E-02 |
| GO: Biological Process | GO:0006959 | humoral immune response | 7.16E-03 | 6.40E-02 |
| GO: Biological Process | GO:0044419 | biological process involved in interspecies interaction between organisms | 7.70E-03 | 6.89E-02 |
| GO: Biological Process | GO:0006735 | NADH regeneration | 7.70E-03 | 6.89E-02 |
| GO: Biological Process | GO:0061621 | canonical glycolysis | 7.70E-03 | 6.89E-02 |
| GO: Biological Process | GO:0061718 | glucose catabolic process to pyruvate | 7.70E-03 | 6.89E-02 |
| GO: Biological Process | GO:0040017 | positive regulation of locomotion | 8.13E-03 | 7.27E-02 |
| GO: Biological Process | GO:0006812 | monoatomic cation transport | 8.13E-03 | 7.27E-02 |
| GO: Biological Process | GO:0030335 | positive regulation of cell migration | 8.30E-03 | 7.43E-02 |
| GO: Biological Process | GO:0051707 | response to other organism | 8.30E-03 | 7.43E-02 |
| GO: Biological Process | GO:0022618 | protein-RNA complex assembly | 8.30E-03 | 7.43E-02 |
| GO: Biological Process | GO:0043207 | response to external biotic stimulus | 8.32E-03 | 7.45E-02 |
| GO: Biological Process | GO:0050921 | positive regulation of chemotaxis | 8.61E-03 | 7.71E-02 |
| GO: Biological Process | GO:0042254 | ribosome biogenesis | 8.61E-03 | 7.71E-02 |
| GO: Biological Process | GO:0060753 | regulation of mast cell chemotaxis | 9.01E-03 | 8.06E-02 |
| GO: Biological Process | GO:0035606 | peptidyl-cysteine S-trans-nitrosylation | 1.01E-02 | 9.02E-02 |
| GO: Biological Process | GO:0061844 | antimicrobial humoral immune response mediated by antimicrobial peptide | 1.01E-02 | 9.03E-02 |
| GO: Biological Process | GO:0000028 | ribosomal small subunit assembly | 1.01E-02 | 9.03E-02 |
| GO: Biological Process | GO:0006417 | regulation of translation | 1.01E-02 | 9.03E-02 |
| GO: Biological Process | GO:0150076 | neuroinflammatory response | 1.01E-05 | 9.03E-02 |
| GO: Biological Process | GO:0016477 | cell migration | 1.03E-02 | 9.21E-02 |
| GO: Biological Process | GO:0009607 | response to biotic stimulus | 1.10E-02 | 9.85E-02 |
| GO: Biological Process | GO:0019677 | NAD catabolic process | 1.10E-02 | 9.85E-02 |
| GO: Biological Process | GO:0061620 | glycolytic process through glucose-6-phosphate | 1.10E-02 | 9.85E-02 |
| GO: Biological Process | GO:0042116 | macrophage activation | 1.19E-02 | 1.07E-01 |
| GO: Biological Process | GO:0002687 | positive regulation of leukocyte migration | 1.22E-02 | 1.09E-01 |
| GO: Biological Process | GO:0097530 | granulocyte migration | 1.25E-02 | 1.12E-01 |
| GO: Biological Process | GO:0002521 | leukocyte differentiation | 1.28E-02 | 1.15E-01 |
| GO: Biological Process | GO:0048002 | antigen processing and presentation of peptide antigen | 1.30E-02 | 1.16E-01 |
| GO: Biological Process | GO:0030595 | leukocyte chemotaxis | 1.30E-02 | 1.17E-01 |
| GO: Biological Process | GO:0006873 | intracellular monoatomic ion homeostasis | 1.31E-02 | 1.17E-01 |
| GO: Biological Process | GO:0022613 | ribonucleoprotein complex biogenesis | 1.32E-02 | 1.18E-01 |
| GO: Biological Process | GO:0002690 | positive regulation of leukocyte chemotaxis | 1.32E-02 | 1.18E-01 |
| GO: Biological Process | GO:0071621 | granulocyte chemotaxis | 1.38E-02 | 1.24E-01 |
| GO: Biological Process | GO:0019730 | antimicrobial humoral response | 1.42E-02 | 1.27E-01 |
| GO: Biological Process | GO:0097529 | myeloid leukocyte migration | 1.59E-02 | 1.42E-01 |
| GO: Biological Process | GO:0002696 | positive regulation of leukocyte activation | 1.63E-02 | 1.46E-01 |
| GO: Biological Process | GO:0045670 | regulation of osteoclast differentiation | 1.80E-02 | 1.61E-01 |
| GO: Biological Process | GO:1901701 | cellular response to oxygen-containing compound | 1.80E-02 | 1.61E-01 |
| GO: Biological Process | GO:1902105 | regulation of leukocyte differentiation | 1.80E-02 | 1.61E-01 |
| GO: Biological Process | GO:0097028 | dendritic cell differentiation | 1.80E-02 | 1.61E-01 |
| GO: Biological Process | GO:1903706 | regulation of hemopoiesis | 1.95E-02 | 1.75E-01 |
| GO: Biological Process | GO:0061615 | glycolytic process through fructose-6-phosphate | 1.95E-02 | 1.75E-01 |
| GO: Biological Process | GO:0150064 | vertebrate eye-specific patterning | 1.95E-02 | 1.75E-01 |
| GO: Biological Process | GO:0050920 | regulation of chemotaxis | 2.01E-02 | 1.80E-01 |
| GO: Biological Process | GO:0050821 | protein stabilization | 2.05E-02 | 1.84E-01 |
| GO: Biological Process | GO:0051050 | positive regulation of transport | 2.09E-02 | 1.87E-01 |
| GO: Biological Process | GO:0006007 | glucose catabolic process | 2.10E-02 | 1.88E-01 |
| GO: Biological Process | GO:0098542 | defense response to other organism | 2.10E-02 | 1.88E-01 |
| GO: Biological Process | GO:0032755 | positive regulation of interleukin-6 production | 2.11E-02 | 1.88E-01 |
| GO: Biological Process | GO:1902806 | regulation of cell cycle G1/S phase transition | 2.13E-02 | 1.91E-01 |
| GO: Biological Process | GO:0032880 | regulation of protein localization | 2.14E-02 | 1.91E-01 |
| GO: Biological Process | GO:1901950 | dense core granule transport | 2.15E-02 | 1.92E-01 |
| GO: Biological Process | GO:0070661 | leukocyte proliferation | 2.19E-02 | 1.96E-01 |
| GO: Biological Process | GO:0150146 | cell junction disassembly | 2.19E-02 | 1.96E-01 |
| GO: Biological Process | GO:0071345 | cellular response to cytokine stimulus | 2.20E-05 | 1.97E-01 |
| GO: Biological Process | GO:0030003 | intracellular monoatomic cation homeostasis | 2.21E-02 | 1.98E-01 |
| GO: Biological Process | GO:0050867 | positive regulation of cell activation | 2.27E-02 | 2.03E-01 |
| GO: Biological Process | GO:0050900 | leukocyte migration | 2.27E-02 | 2.03E-01 |
| GO: Biological Process | GO:0030099 | myeloid cell differentiation | 2.27E-02 | 2.03E-01 |
| GO: Biological Process | GO:0043922 | negative regulation by host of viral transcription | 2.44E-02 | 2.19E-01 |
| GO: Biological Process | GO:1901990 | regulation of mitotic cell cycle phase transition | 2.47E-02 | 2.21E-01 |
| GO: Biological Process | GO:0003254 | regulation of membrane depolarization | 2.47E-02 | 2.21E-01 |
| GO: Biological Process | GO:0032103 | positive regulation of response to external stimulus | 2.49E-02 | 2.23E-01 |
| GO: Biological Process | GO:0098771 | inorganic ion homeostasis | 2.49E-02 | 2.23E-01 |
| GO: Biological Process | GO:0032675 | regulation of interleukin-6 production | 2.56E-02 | 2.29E-01 |
| GO: Biological Process | GO:0006811 | monoatomic ion transport | 2.56E-02 | 2.29E-01 |
| GO: Biological Process | GO:0032943 | mononuclear cell proliferation | 2.57E-02 | 2.30E-01 |
| GO: Biological Process | GO:2000045 | regulation of G1/S transition of mitotic cell cycle | 2.58E-02 | 2.31E-01 |
| GO: Biological Process | GO:0050808 | synapse organization | 2.60E-02 | 2.32E-01 |
| GO: Biological Process | GO:1901987 | regulation of cell cycle phase transition | 2.65E-02 | 2.37E-01 |
| GO: Biological Process | GO:2001198 | regulation of dendritic cell differentiation | 2.65E-02 | 2.37E-01 |
| GO: Biological Process | GO:0002551 | mast cell chemotaxis | 2.65E-02 | 2.37E-01 |
| GO: Biological Process | GO:0032496 | response to lipopolysaccharide | 2.71E-02 | 2.42E-01 |
| GO: Biological Process | GO:1904151 | positive regulation of microglial cell mediated cytotoxicity | 2.73E-02 | 2.44E-01 |
| GO: Biological Process | GO:0014040 | positive regulation of Schwann cell differentiation | 2.73E-02 | 2.44E-01 |
| GO: Biological Process | GO:1901699 | cellular response to nitrogen compound | 2.73E-02 | 2.44E-01 |
| GO: Biological Process | GO:0045944 | positive regulation of transcription by RNA polymerase II | 2.73E-02 | 2.44E-01 |
| GO: Biological Process | GO:0045639 | positive regulation of myeloid cell differentiation | 2.73E-02 | 2.44E-01 |
| GO: Biological Process | GO:0002573 | myeloid leukocyte differentiation | 2.73E-02 | 2.44E-01 |
| GO: Biological Process | GO:0051247 | positive regulation of protein metabolic process | 2.73E-02 | 2.44E-01 |
| GO: Biological Process | GO:0006611 | protein export from nucleus | 2.78E-02 | 2.48E-01 |
| GO: Biological Process | GO:0040012 | regulation of locomotion | 2.78E-02 | 2.48E-01 |
| GO: Biological Process | GO:0098655 | monoatomic cation transmembrane transport | 2.85E-02 | 2.55E-01 |
| GO: Biological Process | GO:1903131 | mononuclear cell differentiation | 2.85E-02 | 2.55E-01 |
| GO: Biological Process | GO:0018119 | peptidyl-cysteine S-nitrosylation | 2.86E-02 | 2.56E-01 |
| GO: Biological Process | GO:0032635 | interleukin-6 production | 2.87E-02 | 2.57E-01 |
| GO: Biological Process | GO:0031647 | regulation of protein stability | 2.96E-02 | 2.65E-01 |
| GO: Biological Process | GO:0140694 | membraneless organelle assembly | 2.98E-02 | 2.66E-01 |
| GO: Biological Process | GO:2000026 | regulation of multicellular organismal development | 2.98E-02 | 2.66E-01 |
| GO: Biological Process | GO:0045597 | positive regulation of cell differentiation | 2.98E-02 | 2.66E-01 |
| GO: Biological Process | GO:0002274 | myeloid leukocyte activation | 3.05E-02 | 2.73E-01 |
| GO: Biological Process | GO:0019882 | antigen processing and presentation | 3.05E-02 | 2.73E-01 |
| GO: Biological Process | GO:0060341 | regulation of cellular localization | 3.11E-02 | 2.79E-01 |
| GO: Biological Process | GO:0034097 | response to cytokine | 3.12E-02 | 2.79E-01 |
| GO: Biological Process | GO:2000145 | regulation of cell motility | 3.12E-02 | 2.79E-01 |
| GO: Biological Process | GO:0017014 | protein nitrosylation | 3.12E-02 | 2.79E-01 |
| GO: Biological Process | GO:0002761 | regulation of myeloid leukocyte differentiation | 3.13E-02 | 2.80E-01 |
| GO: Biological Process | GO:0034341 | response to type II interferon | 3.17E-02 | 2.84E-01 |
| GO: Biological Process | GO:0140546 | defense response to symbiont | 3.25E-02 | 2.91E-01 |
| GO: Biological Process | GO:0071677 | positive regulation of mononuclear cell migration | 3.25E-02 | 2.91E-01 |
| GO: Biological Process | GO:0050850 | positive regulation of calcium-mediated signaling | 3.25E-02 | 2.91E-01 |
| GO: Biological Process | GO:0002474 | antigen processing and presentation of peptide antigen via MHC class I | 3.25E-02 | 2.91E-01 |
| GO: Biological Process | GO:0030097 | hemopoiesis | 3.25E-02 | 2.91E-01 |
| GO: Biological Process | GO:0050801 | monoatomic ion homeostasis | 3.25E-02 | 2.91E-01 |
| GO: Biological Process | GO:0007159 | leukocyte cell-cell adhesion | 3.25E-02 | 2.91E-01 |
| GO: Biological Process | GO:0002688 | regulation of leukocyte chemotaxis | 3.25E-02 | 2.91E-01 |
| GO: Biological Process | GO:0097531 | mast cell migration | 3.29E-02 | 2.94E-01 |
| GO: Biological Process | GO:1901163 | regulation of trophoblast cell migration | 3.29E-02 | 2.94E-01 |
| GO: Biological Process | GO:0032091 | negative regulation of protein binding | 3.29E-02 | 2.94E-01 |
| GO: Biological Process | GO:0006734 | NADH metabolic process | 3.29E-02 | 2.94E-01 |
| GO: Biological Process | GO:1901652 | response to peptide | 3.29E-02 | 2.94E-01 |
| GO: Biological Process | GO:0002237 | response to molecule of bacterial origin | 3.29E-02 | 2.94E-01 |
| GO: Biological Process | GO:0002282 | microglial cell activation involved in immune response | 3.29E-02 | 2.94E-01 |
| GO: Biological Process | GO:1904149 | regulation of microglial cell mediated cytotoxicity | 3.29E-02 | 2.94E-01 |
| GO: Biological Process | GO:0014038 | regulation of Schwann cell differentiation | 3.29E-02 | 2.94E-01 |
| GO: Biological Process | GO:0045963 | negative regulation of dopamine metabolic process | 3.29E-02 | 2.94E-01 |
| GO: Biological Process | GO:0045113 | regulation of integrin biosynthetic process | 3.29E-02 | 2.94E-01 |
| GO: Biological Process | GO:0090634 | microglial cell mediated cytotoxicity | 3.29E-02 | 2.94E-01 |
| GO: Biological Process | GO:0034241 | positive regulation of macrophage fusion | 3.29E-02 | 2.94E-01 |
| GO: Biological Process | GO:1901224 | positive regulation of non-canonical NF-kappaB signal transduction | 3.32E-02 | 2.97E-01 |
| GO: Biological Process | GO:0051251 | positive regulation of lymphocyte activation | 3.42E-02 | 3.06E-01 |
| GO: Biological Process | GO:0042274 | ribosomal small subunit biogenesis | 3.47E-02 | 3.10E-01 |
| GO: Biological Process | GO:0042273 | ribosomal large subunit biogenesis | 3.47E-02 | 3.11E-01 |
| GO: Biological Process | GO:0006954 | inflammatory response | 3.48E-02 | 3.12E-01 |
| GO: Biological Process | GO:0061450 | trophoblast cell migration | 3.50E-02 | 3.13E-01 |
| GO: Biological Process | GO:0070665 | positive regulation of leukocyte proliferation | 3.50E-02 | 3.13E-01 |
| GO: Biological Process | GO:0002684 | positive regulation of immune system process | 3.50E-02 | 3.13E-01 |
| GO: Biological Process | GO:0050729 | positive regulation of inflammatory response | 3.70E-02 | 3.31E-01 |
| GO: Biological Process | GO:0098662 | inorganic cation transmembrane transport | 3.70E-02 | 3.31E-01 |
| GO: Biological Process | GO:0042098 | T cell proliferation | 3.70E-02 | 3.31E-01 |
| GO: Biological Process | GO:0019674 | NAD metabolic process | 3.73E-02 | 3.34E-01 |
| GO: Biological Process | GO:0006754 | ATP biosynthetic process | 3.76E-02 | 3.36E-01 |
| GO: Biological Process | GO:0051046 | regulation of secretion | 3.81E-02 | 3.41E-01 |
| GO: Biological Process | GO:1903530 | regulation of secretion by cell | 3.81E-02 | 3.41E-01 |
| GO: Biological Process | GO:0002396 | MHC protein complex assembly | 3.81E-02 | 3.41E-01 |
| GO: Biological Process | GO:0002501 | peptide antigen assembly with MHC protein complex | 3.81E-02 | 3.41E-01 |
| GO: Biological Process | GO:0034391 | regulation of smooth muscle cell apoptotic process | 4.09E-02 | 3.66E-01 |
| GO: Biological Process | GO:0046651 | lymphocyte proliferation | 4.09E-02 | 3.66E-01 |
| GO: Biological Process | GO:0033077 | T cell differentiation in thymus | 4.11E-02 | 3.68E-01 |
| GO: Biological Process | GO:0071346 | cellular response to type II interferon | 4.11E-02 | 3.68E-01 |
| GO: Biological Process | GO:0030001 | metal ion transport | 4.13E-02 | 3.69E-01 |
| GO: Biological Process | GO:0030162 | regulation of proteolysis | 4.13E-02 | 3.70E-01 |
| GO: Biological Process | GO:0045112 | integrin biosynthetic process | 4.19E-02 | 3.75E-01 |
| GO: Biological Process | GO:0034239 | regulation of macrophage fusion | 4.19E-02 | 3.75E-01 |
| GO: Biological Process | GO:0045914 | negative regulation of catecholamine metabolic process | 4.19E-02 | 3.75E-01 |
| GO: Biological Process | GO:0034314 | Arp2/3 complex-mediated actin nucleation | 4.19E-02 | 3.75E-01 |
| GO: Biological Process | GO:0034390 | smooth muscle cell apoptotic process | 4.19E-02 | 3.75E-01 |
| GO: Biological Process | GO:0031100 | animal organ regeneration | 4.25E-02 | 3.80E-01 |
| GO: Biological Process | GO:0051094 | positive regulation of developmental process | 4.25E-02 | 3.80E-01 |
| GO: Biological Process | GO:0010634 | positive regulation of epithelial cell migration | 4.34E-02 | 3.88E-01 |
| GO: Biological Process | GO:0045595 | regulation of cell differentiation | 4.34E-02 | 3.88E-01 |
| GO: Biological Process | GO:0050670 | regulation of lymphocyte proliferation | 4.49E-02 | 4.02E-01 |
| GO: Biological Process | GO:0043269 | regulation of monoatomic ion transport | 4.49E-02 | 4.02E-01 |
| GO: Biological Process | GO:0030334 | regulation of cell migration | 4.49E-02 | 4.02E-01 |
| GO: Biological Process | GO:0042053 | regulation of dopamine metabolic process | 4.50E-02 | 4.03E-01 |
| GO: Biological Process | GO:0045785 | positive regulation of cell adhesion | 4.59E-02 | 4.10E-01 |
| GO: Biological Process | GO:2000403 | positive regulation of lymphocyte migration | 4.64E-02 | 4.15E-01 |
| GO: Biological Process | GO:2001233 | regulation of apoptotic signaling pathway | 4.82E-02 | 4.31E-01 |
| GO: Biological Process | GO:0030316 | osteoclast differentiation | 4.85E-02 | 4.34E-01 |
| GO: Biological Process | GO:0032944 | regulation of mononuclear cell proliferation | 4.85E-02 | 4.34E-01 |
| GO: Biological Process | GO:0044843 | cell cycle G1/S phase transition | 4.85E-02 | 4.34E-01 |
| GO: Biological Process | GO:0055080 | monoatomic cation homeostasis | 4.85E-02 | 4.34E-01 |
| GO: Biological Process | GO:0018198 | peptidyl-cysteine modification | 4.85E-02 | 4.34E-01 |
| GO: Biological Process | GO:0050848 | regulation of calcium-mediated signaling | 4.87E-02 | 4.36E-01 |
| GO: Biological Process | GO:0051899 | membrane depolarization | 4.87E-02 | 4.36E-01 |
| GO: Biological Process | GO:0001819 | positive regulation of cytokine production | 4.89E-02 | 4.38E-01 |
| GO: Biological Process | GO:0046034 | ATP metabolic process | 4.90E-02 | 4.38E-01 |
| GO: Biological Process | GO:0045087 | innate immune response | 4.93E-02 | 4.41E-01 |

Complete list of significant (q-value FDR B&H < 0.05) enriched Gene Ontology Biological process. Functional annotations indicate predicted altered pathways in hippocampal microglia from Casp3 mice compared to Sham.
